# Detecting and Grouping In-Source Fragments with Low-Energy Stepped HCD, Together with MS^3^, Increases Identification Confidence in Untargeted LC–Orbitrap Metabolomics of *Plantago lanceolata* Leaves and *P. ovata* Husk

**DOI:** 10.3390/metabo16010042

**Published:** 2026-01-02

**Authors:** Vilmantas Pedišius, Tim Stratton, Lukas Taujenis, Valdas Jakštas, Vytautas Tamošiūnas

**Affiliations:** 1 Thermo Fisher Scientific Baltics, 03227 Vilnius, Lithuania; 2 Department of Pharmacognosy, Faculty of Pharmacy, Lithuanian University of Health Sciences, 50166 Kaunas, Lithuania; 3 Thermo Fisher Scientific, Austin, TX 78701, USA

**Keywords:** metabolomics, Plantago, mass spectrometry, UHPLC, compound identification, low-energy HCD, in-source fragmentation

## Abstract

**Background:** Comprehensive and accurate compound composition characterization in natural sources has high relevance in food and nutrition, health and medicine, environmental and agriculture research areas, though profiling of plant metabolites is a challenging task due to the structural complexity of natural products. This study delves into the identification and characterization of compounds within the Plantago genus, leveraging state-of-the-art analytical techniques. **Methods:** Utilizing an ultra-high-performance liquid chromatography (UHPLC) system in conjunction with Orbitrap™ IQ-X™ Tribrid™ mass spectrometer (MS), we employed a Phenyl-Hexyl HPLC column alongside optimized extraction protocols to analyze both husk and leaf samples. To maximize compound identification, we implemented data-dependent acquisition (DDA) methods including MS^2^ (ddMS2), MS^3^ (ddMS3), AcquireX™ deep scan, and real-time library search (RTLS). **Results:** Our results demonstrate a significant increase in the number of putatively yet confidently assigned compounds, with 472 matches in *P. lanceolata* leaves and 233 in *P. ovata* husk identified through combined acquisition methods. The inclusion of an additional fragmentation level (MS^3^) noticeably enhanced the confidence in compound annotation, facilitating the differentiation of isomeric compounds. Furthermore, the application of low-energy fragmentation (10 normalized collision energy (NCE) for higher-energy collisional dissociation (HCD)) improved the detection and grouping of MS^1^ fragments by 55% in positive mode and by 16% in negative mode, contributing to a more comprehensive analysis with minimal loss in compound identification. **Conclusions:** These advancements underscore the potential of our methodologies in expanding the chemical profile of plant materials, offering valuable insights into natural product analysis and dereplication of untargeted data.

## 1. Introduction

Comprehensive profiling is crucial both for understanding compositional properties and for planning phytotherapeutic formulations, as the lack of thorough compound identification hinders compositional knowledge and may impair therapeutic effect design or product formulation refinement [1,2]. Furthermore, broad profiling supports the elucidation of mechanisms of action and the evaluation of potential therapeutic targets or disease-modulating applications [3]. While comprehensive untargeted metabolomics has been applied to several Plantago species—most notably revealing 1216 features obtained in negative-ion ultra-high-performance liquid chromatography–electrospray ionization–high-resolution mass spectrometry (UHPLC-ESI-HRMS) and 4654 in positive mode from Plantago lanceolata seed extracts—the majority of detected features remained not fully grouped or resolved into putative identifications [4,5,6,7].

High-confidence metabolite identification, as defined by the Metabolomics Standards Initiative (MSI) Level 1, traditionally relies on the availability of standards or the purification of metabolites followed by nuclear magnetic resonance (NMR) confirmation [8,9,10]. However, these approaches are labor-intensive and might be impractical at certain stages of phytochemical development. Therefore, putative identifications (MSI Level 2) are frequently employed, relying on the comparison of several distinct analytical attributes, such as retention time, accurate mass, and tandem mass spectra, against entries in spectral libraries or databases acquired under identical experimental conditions [9,11,12,13,14,15]. Even with these strategies, the complexity of structural isomers and limited MS^2^ spectral databases leave some uncertainty in compound identification [8,10].

Fragmentation of precursor ions may be insufficient to differentiate between closely related isomers or reveal substructural details, especially in the case of complex plant metabolites [16,17]. In-source fragmentation (ISF) adds additional complexity to the MS^1^ data, creating more peaks that need to be processed and matched [18]. This increased complexity can lead to ambiguous matches where several candidate compounds may fit the observed spectral features, increasing the likelihood of a false positive. For example, in a study by Mahieu et al., only ~4% of 25,000 measured *m*/*z* features in *Escherichia coli* samples originated from true metabolites [19]. That emphasizes a need to detect and group MS^1^ fragments early, since many secondary—including glycosides, phenolics, and some alkaloids—are intrinsically fragile and prone to fragmentation.

In this work, an untargeted metabolomics approach for the fast, broad-scale, confidence-oriented characterization of *Plantago lanceolata* (ribwort plantain) leaves and *Plantago ovata* (blond plantain) husk using Tribrid™ instruments is applied [20]. By incorporating a low-energy MS^2^ step in a multi-energy stepped HCD MS^2^ fragment scan together with obligatory MS^3^ fragmentation [21,22], we improved structural confidence and increased average mzCloud best match (BM) scores by ~7%. Additional acquisition strategies such as real-time library search [23,24] and AcquireX™ [25], similar to IE-Omics [26], preserved broad coverage of identifications without reducing overall detection numbers. The methodology addresses the advantages of MS^3^-based workflows and shows how incorporating a low-energy normalized collision energy (NCE) step makes in-source fragments (ISFs) detectable in MS^2^, enabling them to be correctly grouped with their parent precursors.

## 2. Materials and Methods

### 2.1. Sample Preparation

Dried ribwort plantain (*Plantago lanceolata* L.) leaves (Lot No. Ž422301/4/1/2023/0/1, Lithuania) and blond plantain (*Plantago ovata* Forssk.) husk (Lot No. Ž192311/FG23070102, origin India) were obtained from UAB, Švenčionių vaistažolės (Švenčionys, Lithuania). Certificates of Analysis confirmed compliance with Ph. Eur. specifications for identity, purity, pesticide residues, and microbiological safety. The raw materials were milled using an MF 10 basic Microfine grinder drive (IKA, Staufen, Germany) fitted with a 0.25 mm sieve, and 1.00 g of each powdered sample was taken for extraction. Each sample was combined with 2 mL of Optima™ LC/MS-grade water (Fisher Chemical™, Waltham, MA, USA), thoroughly mixed, and followed by the addition of 3 mL Optima™ LC/MS-grade acetonitrile (Fisher Chemical™, Waltham, MA, USA). The samples were extracted for 4 h using a Grant PHMP-4 Thermoshaker for Microplates (Grant Instruments, Cambridge, UK) at 300 rpm and room temperature, then sonicated for 5 min in a Fisherbrand Ultrasonic Cleaner (Elmasonic P 120 H, Elma Schmidbauer GmbH, Singen, Germany). After centrifugation at 4000 rpm for 5 min using a Thermo Scientific Multifuge X4R Pro-MD (Thermo Fisher Scientific, Bremen, Germany), the supernatant was filtered through a 0.2 µm Titan3™ PVDF syringe filter (Rockwood, TN, USA) and stored at −70 °C in a TSX Series ultra-low freezer (Thermo Fisher Scientific, Asheville, NC, USA) prior to analysis.

### 2.2. Chromatographic Separation

Chromatographic separation was performed using an HPLC-HRMS system, comprising a Vanquish™ Horizon LC system, consisting of a binary pump H, split sampler FT with a 25 µL sample loop and a column compartment H. Separation was achieved on an Accucore™ Phenyl-hexyl column (100 × 2.1 mm, 2.6 µm) through binary gradient at a flow rate of 0.5 mL·min^−1^ with the mobile phase A consisting of water with 0.1% formic acid and mobile phase B consisting of acetonitrile (ACN) with 0.1% formic acid. The gradient elution was as follows: 0.00 min, 95% A; 1.00 min, 95% A; 14.50 min, 20% A; 14.51 min, 1% A; 15.50 min, 1% A; 15.51 min, 95% A. The gradient and injection volume were optimized using standards of hippuric acid, ferulic acid, catechin, and ajmalicine. These four compounds represent distinct phytochemical classes (aromatic acid, phenolic acid, flavonoid, and indole alkaloid, respectively) and span a wide range of pKa values—approximately 3.6, 4.6, 9, and 7.4, respectively. By choosing diverse standards, we ensured that analytes with different chemical properties (from highly acidic to weakly acidic phenolics and basic alkaloids) could all be retained and effectively separated under the chosen gradient.

### 2.3. Mass Spectrometry

Orbitrap IQ-X Tribrid mass spectrometer was utilized for analysis. Data acquired in both positive and negative ionization modes, with ionization conditions optimized based on prior research [27,28,29,30]. The H-ESI source was set and operated at following parameters: 3.6 kV (positive) and 2.6 kV (negative), with sheath gas 40 Arb, auxiliary gas 10 Arb, sweep gas 1 Arb, vaporizer 400 °C, and ion-transfer tube 350 °C. MS^1^ scans were acquired at 240,000 resolution and MS/MS scans at 30,000 resolution, at a full scan range of 80–1200 *m*/*z* [27,28,29,30]. For the master scan, quadrupole isolation was used with a radio frequency ion optics lens (RF lens) set to 70%. Automatic gain control (AGC) target and maximum injection time were set to standard and auto values, respectively, based on default method settings. Intensity filters were applied with an intensity threshold of 2.0 × 10^4^. Dynamic exclusion for isotopes was enabled after one occurrence (2.5 s exclusion duration, ±10 ppm tolerance). The ddMS2 OT HCD parameters were set as follows: quadrupole isolation mode, with an isolation window of 1.2 *m*/*z* and isolation offset set to off. Activation type was HCD, using stepped collision energy mode with normalized HCD collision energies of 10%, 50%, and 80%. The detector type was Orbitrap, with a resolution of 30,000. The scan range mode was set to automatic, AGC target to standard, and maximum injection time mode to automatic. Microscans were set to 1. In ddMS3 experiments (including real-time library search and AcquireX deep scan modes), an additional subsequent HCD fragmentation step was performed for MS^3^ analysis, acquired at 15,000 resolution and NCE 30% with a maximum injection time of 22 ms. The duty cycle differed from ddMS2 by using a smaller number of dependent scans: TopN = 4 for MS^2^ precursors and TopN = 3 for MS^3^ events. These MS^3^-based methods therefore differed primarily by their triggering logic, where ddMS3 employed standard data-dependent precursor selection, RTLS triggered MS^3^ only when library match confidence was below a defined threshold (Cosine Score ≥ 60), and AcquireX deep scan applied seven iterative precursor exclusion and inclusion lists across injections to increase metabolome coverage. Full details for individual parameters of ddMS3 and MS^3^ (ddMS3, RTLS and AcquireX deep scan) acquisition methods are presented in Figure S1.

### 2.4. Data Analysis

Data analysis was performed using Compound Discoverer™ 3.4 with an untargeted data analysis workflow (details of the workflow are provided in Figure S2). Prior to analysis, six different workflows were tested. The “maxID” workflow, which provided the highest number of compound identifications from a known natural product mixture injection, was selected for all subsequent sample analyses. Briefly, raw data were imported and full-scan features detected with a mass tolerance of 5 ppm, precursor mass tolerance of 0.025 Da, minimum peak intensity of 1.0 × 10^4^, chromatographic signal-to-noise threshold of 1.2, and a maximum MS tree depth of 3; isotope grouping was enabled for Cl and Br adducts. Detected features were then grouped using a retention-time tolerance of 0.2 min, minimum valley of 10%, alignment aggression of 0.3, area contribution of 3, and peak rating threshold of 4. Molecular formulas were assigned via accurate-mass and isotope fitting, and MS^2^ spectra searched against the mzCloud online library (cosine scoring; precursor tolerance 10 ppm; fragment tolerances 2.5 mmu high-accuracy/0.5 Da low-accuracy; activation energy tolerance 40; mass factor threshold 60). Exact-mass searches against user-defined mass lists and ChemSpider further captured formula-only annotations. Subsequent post-processing analysis and figure generation were performed in Python 3.11.3. The identities were determined based on specific criteria. A compound was accepted if it overlaps across all three MS^3^ methods (ddMS3, RTLS, and AcquireX Deep Scan) with at least one mzCloud BM score being above 80; it overlaps in at least two of the MS^3^ methods with at least one score being above 80; it overlaps across all four methods (ddMS2, ddMS3, RTLS, and AcquireX Deep Scan) with at least one score being above 90; it overlaps in at least three of the methods with at least one score being above 90; and it is identified by at least one of the MS^3^ methods with at least one score being above 95. The accepted set of compounds *z*, denoted as *Z*, is defined as follows:

(1) $$\begin{array}{cc} Z & =\left\{\left[z\in B\cap C\cap D\wedge \max\left\{s_{B}\left(z\right),s_{C}\left(z\right),s_{D}\left(z\right)\right\}\geq 0.80\right]\right.\\ & ˅\left[z\in \left(B\cap C\right)\cup \left(B\cap D\right)\cup \left(C\cap D\right)\wedge \max\left\{s_{B}\left(z\right),s_{C}\left(z\right),s_{D}\left(z\right)\right\}\geq 0.80\right]\\ & ˅\left[z\in A\cap B\cap C\cap D\wedge \max\left\{s_{A}\left(z\right),s_{B}\left(z\right),s_{C}\left(z\right),s_{D}\left(z\right)\right\}\geq 0.90\right]\\ & ˅\left[z\in U_{i<j<k}\left(M_{i}\cap M_{j}\cap M_{k}\right)\wedge \max\left\{s_{A}\left(z\right),s_{B}\left(z\right),s_{C}\left(z\right),s_{D}\left(z\right)\right\}\geq 0.90\right]\\ & \left.˅\left[z\in B\cup C\cup D\wedge \max\left\{s_{B}\left(z\right),s_{C}\left(z\right),s_{D}\left(z\right)\right\}\geq 0.95\right]\right\} \end{array}$$

where *A* = ddMS2, *B* = ddMS3, *C* = RTLS, *D* = AcquireX Deep Scan. The function *s_X_*(*z*) represents the mzCloud BM score of compound *z* in method *X*, and *max*{} denotes maximum score among the specified methods, e.g., *max*{*s_B_*(*z*),*s_C_*(*z*),*s_D_*(*z*)} means “at least one of the three MS^3^ methods gave *z* a match ≥ 0.80.” The term *U_i<j<k_*(*M_i_* ∩ *M_j_* ∩ *M_k_*) denotes all possible three-way intersections among the four acquisition modes. To evaluate the sensitivity of *Z* to the mzCloud BM thresholds, *Z* is recalculated under three alternative threshold triplets (70/80/90, 75/85/90, and 85/92/97). All main results in this study use the primary thresholds of 80/90/95.

## 3. Results

### 3.1. MS Parameter Optimization

**Figure 1:**
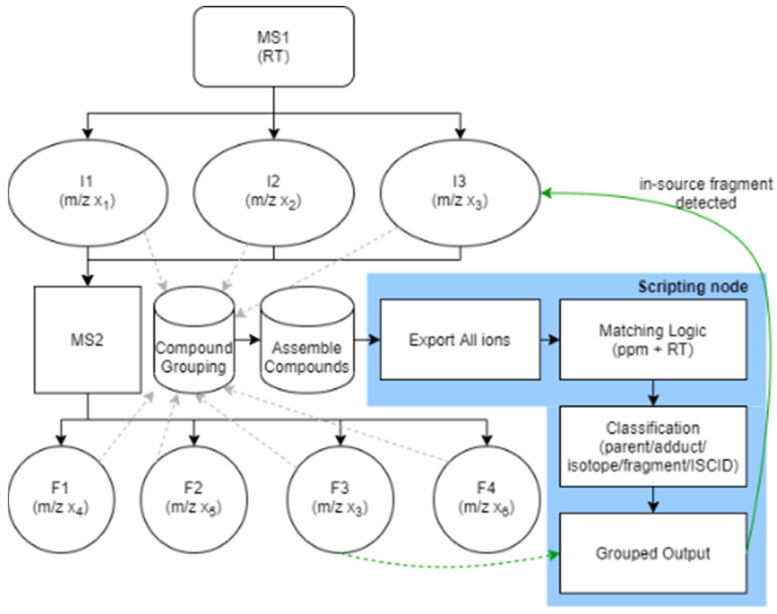
Conceptual schematic of in-source fragment detection and grouping in Compound Discoverer™. MS^1^ fragment detection and grouping occurs during compound assembly (Assemble Compounds node), while a post-processing routine of the scripting node exports the MS1 fragment data for further investigation. Solid arrows indicate standard data flow within Compound Discoverer™, dashed arrows indicate inferred grouping relationships, and green arrows highlight detected in-source fragments re-associated during post-processing. Abbreviations: RT—retention time; I—ion; F—fragment.

Proper tuning of interface parameters is essential for reducing ISF and improving annotation confidence [30,31]. Even with this adjustment, labile molecules in untargeted metabolomics analysis can be over-fragmented and later misannotated. Therefore, dedicated strategies to detect and group ISFs are needed to recover components that may otherwise be overlooked, which in part is compensated for by using stepped collision energies [32]. Incorporating a low-energy NCE step would preserve labile, over-fragmented ions that are otherwise completely dissociated at higher collision energies. These ions could then be detected and grouped with their parent precursors during downstream feature grouping and through Assemble Compounds node of the Compound Discoverer™, where the program algorithm integrates MS^1^ features with MS^2^ fragments and classifies ISFs alongside adducts and isotopes. The scripting node is then used to export these classifications (see Figure 1).

**Figure 2:**
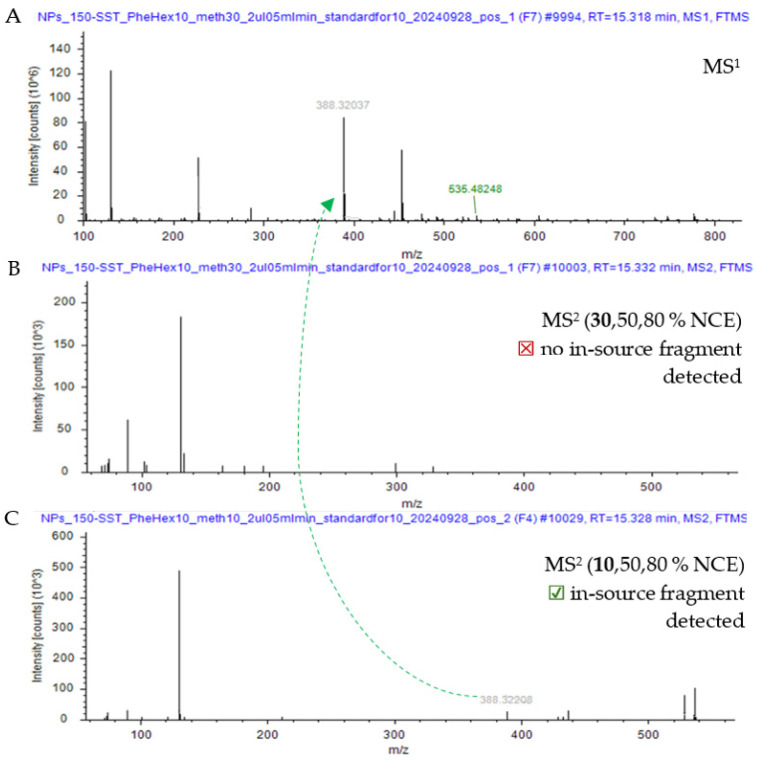
Steps to group MS^1^ fragments using MS^2^ spectra (Sample: *P. lanceolata* leaf extract; ddMS^2^, Top10). Full MS^1^ (+) at 15.32 min. for the detection of peak 535.4825 showing the signal for 388.3204 (**A**). HCD MS^2^ fragment spectra of 535.4825 using standard stepped 30/50/80% NCE conditions (**B**). HCD MS^2^ fragment spectra of 535.4825 using stepped 10/50/80% NCE showing signal for higher *m*/*z* ions including 388.3221 (**C**). The dashed arrow indicates the correspondence between the MS^1^ in-source fragment and its detection in the respective MS^2^ spectra under lowered stepped NCE conditions.

To test this hypothesis, a representative case shown in Figure 2 is taken for examination. The MS^1^ spectrum shows a precursor at *m*/*z* 535.4825 together with peaks at *m*/*z* 388.3204 (A). Under typical stepped HCD conditions (30/50/80% NCE), the precursor is fully fragmented, but no in-source fragment peaks are evident (B). By contrast, when a low-energy step (10% NCE) is introduced alongside 50% and 80% (C), the MS^2^ spectrum revealed a fragment at *m*/*z* 388.3221 corresponding to the in-source fragment originally observed in MS^1^. Extracted ion chromatograms confirming co-elution of *m*/*z* 535.4825 and 388.3204 are provided in Supplementary Figure S3.

**Figure 3:**
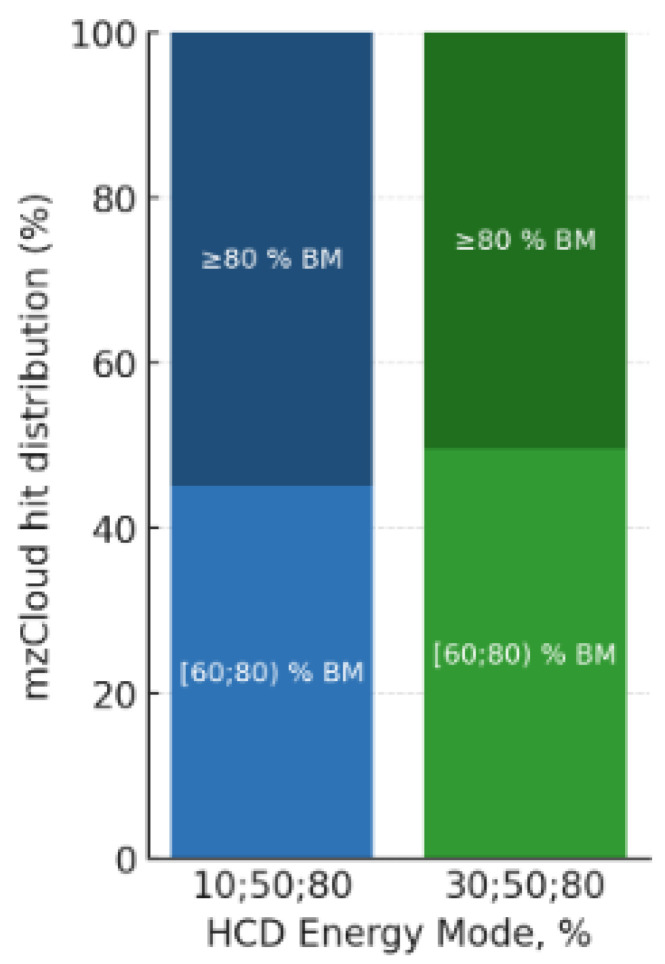
Comparison of lower and standard energy 3-step acquisition in positive mode (ddMS^2^, Top10, NCE 10/50/80% vs. 30/50/80%; *P. lanceolata* leaf extract).

Optimization of fragmentation energies demonstrated that a three-step (10/50/80% NCE) scheme increased the number of MS^1^ fragment ions captured and grouped together by an average of 55% in positive mode (rising from 107–112 to 162–175) and by 16% in negative mode (from 33–34 to 38–42) compared to the standard 30/50/80% NCE regime (SDs 3.5% and 3.0%, respectively). Improved grouping refers to an increased proportion of MS^1^ fragment peaks correctly assigned to their parent compounds across 3 replicates. In contrast, expanding the scheme to four steps (10, 30, 50, 80% NCE) offered no further gain in fragment detection (105–108 ISFs), suggesting diminishing returns when ion populations are split across additional collision energies. The total number of compounds returning a library hit with a score of above a minimum of 60% was almost the same regardless of the acquisition using lower energy 3-step acquisition or the standard 30/50/80% NCE in positive mode. However, with a Cosine spectral match score above 80, the threshold for a “good” hit, was slightly better in both positive (+11%) and negative mode (+25%) for the lower energy 3-step acquisition as indicated by a slight difference of ≥80 BM in Figure 3.

Beyond collision energies, precursor isolation width and number of precursors per cycle (TopN) settings were optimized. The isolation window of 1.2 *m*/*z* outperformed the 2.0 *m*/*z* window with a yield of 4–5% under identical conditions, providing more reliable hits and improving the overall data quality. Among the DDA settings tested, the Top10 precursor selection offered the better balance between coverage and reliability than Top4, and was therefore adopted in subsequent ddMS2 analyses. 

### 3.2. Comparison Between Acquisition Modes

**Figure 4:**
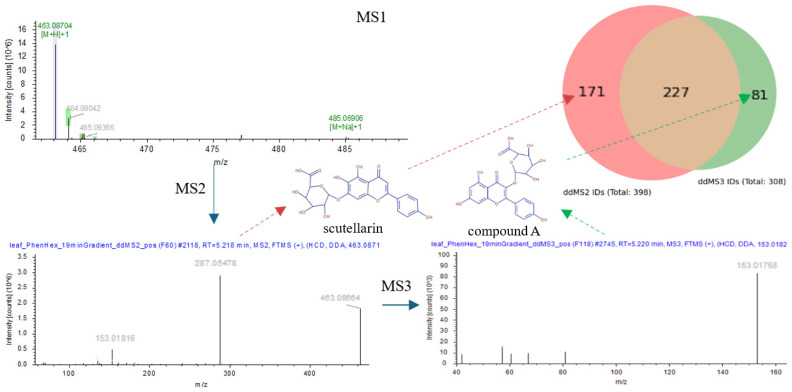
Comparison of ddMS2 (Top10) and ddMS3 (MS^2^ Top4; MS^3^ Top3) methods in analyzing *Plantago lanceolata* leaves in positive mode together with showcase of enhanced structural annotation through MS^3^ fragmentation.

Two analytical methods—ddMS2, ddMS3—were compared analyzing *Plantago lanceolata* leaves with a above 80 mzCloud BM score, revealing significant overlaps and unique identifications. As shown in Figure 4, the Venn diagram on the top right illustrates that the total overlap between the methods is 227 compounds. Compounds unique to each method included 171 identified solely by ddMS2 and 81 by ddMS3. Examples of unique isobaric compounds found in ddMS2 and ddMS3 are scutellarin and a flavonoid glycoside, lacking a confirmed trivial name (Compound A), respectively. As further shown in Figure 4 fragmentation of the protonated molecule at *m*/*z* 463.0871 yields a primary MS^2^ loss of 176 Da, indicative of glucuronic acid cleavage, and produces key ions at *m*/*z* 287.0549 and 153.0182. Given only the MS^2^ spectrum, the spectral search algorithm returned a best match to scutellarin (7-O-glucuronide); however, subsequent MS^3^ of the retro-Diels–Alder (RDA) A-ring fragment at *m*/*z* 153.01758 (A-ring, ^1,3^A/RDA ion; diagnostic A-ring fragment formed by retro-Diels–Alder cleavage of the flavonoid core) reveals lower-mass fragments whose pattern is consistent with the hydroxylation arrangements supporting a 3-O-substituted isomer over an 7-O-substituted alternative. In this case, the ddMS3 data disfavored the MS^2^-based match to scutellarin and instead supported Compound A, which shares the same precursor but exhibits the diagnostic A-ring fragment pattern observed in MS^3^. As a result, the overall mzCloud BM score increased by 7% (from an average of 85% using ddMS2 to 92% using ddMS3) within a 60 to 100 percent BM threshold used in library search. The non-overlapping isomers between ddMS2 and ddMS3, besides flavonoid glycoside discussed, primarily include aromatic aldehydes (e.g., p-tolualdehyde, acetophenone), indole derivatives (e.g., indole-3-acrylic acid), amino acids (e.g., tryptophan), triterpenoids (e.g., 18-β-glycyrrhetinic acid), flavonoids and fatty acid derivatives. 

In addition to the scutellarin case shown in Figure 4, 31 other MS^3^-assisted refinements were observed across different metabolite classes, accounting for approximately 14% of the overlaps of the two acquisition methods. For example, MS^3^ differentiated aromatic phenolics (p-tolualdehyde to acetophenone), resolved positional variation in indole derivatives (indole-3-acrylic acid to trans-3-indoleacrylic acid), and refined flavonoid annotations, where two distinct precursors initially matched to kaempferol and dracocephaloside in MS^2^ were both reassigned to luteolin-based structures (luteolin and luteolin 4′-O-glucoside) after MS^3^ analysis. These representative cases are summarized in Supplementary Table S1.

To address the longer cycle times of ddMS3 while maintaining the same MS scan depth of 3 for reliability and ensuring the detection of medium to low abundant compounds, AcquireX™ deep scan and Real-Time Library Search acquisition techniques were employed. To evaluate the contribution of different acquisition strategies, both ddMS2 and ddMS3 datasets were analyzed with a focus on confident identifications using mzCloud. Only compounds with a BM score of ≥80% were considered for putative identifications (Level 2) ensuring sufficient annotation confidence. Table 1 summarizes the total number of compounds detected across acquisition modes, including MS depth, polarity, and various detection amounts. The table includes the number of compounds with full spectral matches above ≥60% and ≥80% scores. Incorporation of the AcquireX™ deep scan workflow increased the total number of MS^2^ and MS^3^ events by ~25% and identified the largest number of unique compounds, capturing low-abundance ions that were missed in conventional DDA runs, underscoring its effectiveness in detecting low-abundance metabolites through iterative DDA runs. Using the criteria defined in Equation (1), which integrates results across ddMS2, ddMS3, RTLS, and AcquireX™ Deep Scan, a total of 472 compounds in *P. lanceolata* leaves and 233 in *P. ovata* husk have been putatively identified; the complete list of compounds is provided in Supplementary Material Table S2.

To validate the identification workflow, a 143-compound natural product standard mixture was analyzed and Compound Discoverer processing parameters were empirically optimized according to acquired results. Out of 143 compounds, 122 were correctly identified in the neat mixture, corresponding to an 85.3% confirmation rate. When the mixture was analyzed in the presence of the *P. lanceolata* extract matrix, 113 compounds were still recovered, demonstrating that matrix effects did not substantially degrade mzCloud matching performance or the overall reliability of the workflow. To evaluate the dependence of identification yield on the selected BM threshold, alternative acceptance levels were explored. Relaxing the criteria to 70/80/90 increased the number of accepted compounds to approximately 130, while an intermediate setting of 75/85/90 yielded 126 accepted compounds, closely matching the baseline result. In contrast, tightening the criteria to 85/92/97 resulted in a pronounced decrease to approximately 96 confirmed compounds. 

### 3.3. Comparison Between Husk and Leaf Samples

**Figure 5:**
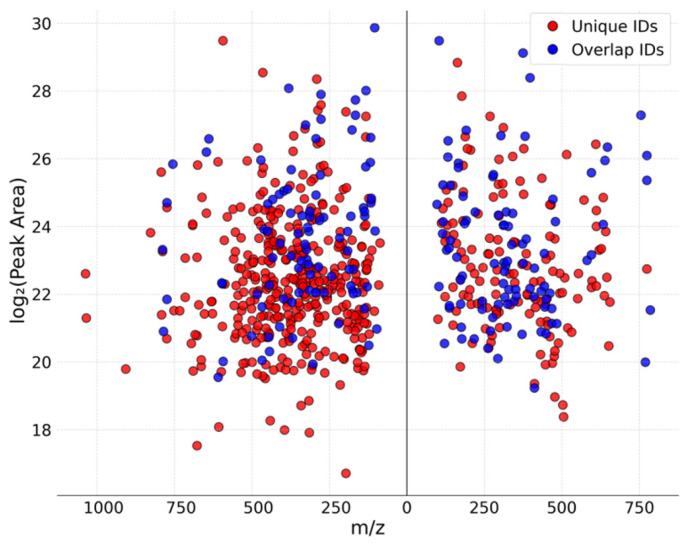
Plot diagram illustrating the overlap of identified compounds between *Plantago lanceolata* leaves (**left**) and *Plantago ovata* husk (**right**). Acquisition mode: combined ddMS^2^, ddMS^3^, RTLS and AcquireX.

Chemical profile similarities between husk and leaf samples were visualized based on shared compound identifications from the spectral library. Figure 5 shows each compound’s log_2_-transformed peak area on a mirror scatter plot, highlighting key differences in *P. lanceolata* leaves and *P. ovata* husk. Of the 611 total unique compounds putatively identified across both sample types, 94 compounds were present in both leaf and husk, while 378 were exclusive to leaves and 139 were exclusive to husk. This shared core—comprising abundant amino acids and organic acids (e.g., L-phenylalanine, L-valine, L-malic acid, suberic acid, trans-aconitic acid)—reflects conserved metabolic functions across tissues. Other overlapping secondary metabolites included flavonoids (e.g., luteolin glycosides, rhamnetin) and lipid-derived molecules (erucamide, monopalmitin), which occurred at comparable levels in both samples.

Leaf-specific profile was dominated by polyphenolic and alkaloid compounds: for instance, flavonoids such as apigenin, diosmetin and the flavonoid glycoside trifolin (all high in leaves) were essentially absent in the husk samples. Likewise, phenolic acids (chlorogenic acid, gentisic acid, neochlorogenic acid) and vitamins or their derivatives (pyridoxine, D-α-tocopherol) were abundant in leaves but below detection in the husk. By contrast, husk tissue contained relatively higher levels of amino-, fatty acids, and oxo-acids. For example, L-glutamic acid, phloionolic acid, sinapine and certain derivatives (e.g., N-(Carboxyacetyl)-L-tryptophan, 8-Hydroxy-9,10-epoxystearic acid) showed much higher peak areas in husk tissue, suggesting that seed-coat metabolism favors accumulation of storage or defense compounds, in contrast to the specialized phenolics of the photosynthetic leaf. 

## 4. Discussion

In electrospray ionization mass spectrometry, ISF occurs during the transfer of ions from the atmospheric-pressure source into the vacuum of the mass spectrometer. As ions transition from the ambient pressure into the intermediate-pressure regions of the instrument, they are accelerated by steep pressure drop. These ions collide with neutral gas molecules in the interface, and each collision can impart enough energy to break apart a labile molecular ion. In this way, fragment ions may be produced up-front—before the analyte reaches the mass analyzer—resulting in additional peaks that appear in the full MS1 spectrum as if they were independent species [33,34,35]. The representative case shown in Figure 2 demonstrates how adding a low-energy collision energy step allows ISFs to be explicitly detected and correctly grouped with their parent precursor. Reducing the number of stepped collision energies from four to three concentrates a greater proportion of the ion population into each HCD event (≈33% vs. ≈25% per step) [36], thereby boosting fragment signal intensity and detectability. The improved performance of the 1.2 *m*/*z* isolation window likely reflects the reduced co-isolation of adjacent ions with a narrower window, which improves MS2 precursor selection, even while possibly cutting overall ion transmission and losing sensitivity [37,38]. Taken together, these optimization results support the use of low-energy fragmentation conditions, optimal chosen amount of the stepped collision energies and a smaller isolation window (1.2 *m*/*z*) for optimizing compound detection and improving the identification of metabolites in complex samples.

Even though ddMS2 showed more identified compounds, ddMS3 had more selective identification in terms of isomeric differentiation. This difference arises from the deeper fragmentation achieved in ddMS3: when only an MS2 spectrum is available, the search algorithm returns the best possible match based on the fragments observed in that spectrum. When MS3 data are incorporated, the additional structural ions supplement the MS2 evidence, allowing the match to be refined when consistent or revised when contradictory, enhancing the annotation process. Thus, by adding the third stage of fragmentation, the sugar attachment site can be more confidently inferred, demonstrating how MS3 data enriches structural annotation beyond what conventional MS2 workflows provide—particularly as spectral libraries grow to include novel compounds with overlapping isobaric masses and fragmentational similarities. These results illustrate the reliability of ddMS3, as it provides more consistent and higher mzCloud BM scores compared to ddMS2, suggesting that ddMS3 acquisition mode can provide more accurate library matches than ddMS2.

The different acquisition workflows provided distinct trade-offs between coverage and spectral match quality. By leveraging iterative inclusion lists in AcquireX™, redundant fragmentation of high-abundance species was minimized, and broader metabolite sampling was achieved. Negative-mode AcquireX™ was performed in a single Deep Scan iteration; additional iterations would be expected to increase coverage, particularly for low-abundance features. Therefore, negative-mode leaf comparisons based on AcquireX should be interpreted as a lower-bound estimate rather than an exhaustive comparison. AcquireX’s strength—deep, iterative sampling of precursors—also brings inherent trade-offs in spectral BM score. This could be caused by sampling many low-abundance ions which generates MS2 or MS3 spectra with weaker fragment peaks and lower signal-to-noise ratios. Furthermore, scoring thousands of spectra rather than only a few hundred, may have inevitably lowered the average match factor by statistical dilution. In parallel, RTLS acquisition strategy triggered MS3 in real time whenever an acquired MS2 spectrum matched a reference spectrum in the mzCloud library above a defined similarity threshold. It is worth noting that the acquisition algorithm used only one-fifth of the auto-processed mzCloud library yet still reduced unnecessary MS3 scans on well-characterized precursors and freed the duty cycle for additional MS2 sampling. Taken together, AcquireX™ gives broader coverage, RTLS increases the proportion of high-confidence identifications, and the addition of MS3 fragmentation further enhances mzCloud BM scores.

In the 143 compound natural product standard mixture validation, the baseline 80 threshold was ultimately retained, as it provided a balanced trade-off between coverage and confidence, and showed a reduction in false positive identifications. It should be not-ed that BM scores provide confidence in spectral matches but cannot eliminate the risk of isomeric misannotation inherent to MSI Level 2 identifications; hence, future orthogonal confirmation, such as retention-index comparison, ion mobility separation, or purification followed by NMR would further strengthen ambiguous cases. However, availability of MS3 data and the limited number of identification reassignments observed between MS2-only and MS3-supported workflows indicate a measurable increase in annotation confidence.

The leaf- and husk-specific profiles are consistent with the physiological roles of each organ: leaf-enriched flavonoids absorb UV and protect the photosynthetic tissues [39], whereas the husk (seed coat) concentrates primary metabolites and lipid-related compounds for seed development. In summary, leaves contained a richer palette of flavonoids, phenolic acids and vitamins, whereas the husk shared basic amino/fatty acids and some shared flavonoids but lacked many of the leaf-specialized phenolics (see Table S2 for the complete list). Plant metabolomics studies employing untargeted LC–MS/MS typically detect thousands of spectral features, of which approximately up to 400 metabolites per experiment have been putatively identified at MSI Level 2 through database or spectral library matching. Reported confidence thresholds vary: some studies do not specify a numerical cutoff, while others apply similarity scores in the range of 65–80%. For example, Contreras-Angulo et al. (2022) used in silico annotation with compound structure identification: FingerID (CSI:FingerID) and required a confidence of small molecule identifications (COSMIC) scoring > 0.65 (~65%) to accept a candidate structure [40]. Fan et al. (2020) reported 364 putative metabolites across multiple *Clausena lansium* tissues, though without declaring a confidence threshold [41], and Yuan et al. (2022) annotated 305 metabolites in *Salvia miltiorrhiza* roots under cadmium stress via database matching [42]. Similarly, Allwood et al. (2019) putatively identified 155 metabolites in *Ribes nigrum* fruit at MSI Level 2 confidence [20]. More recently, Zhang et al. (2022) demonstrated a specialized workflow for comprehensive annotation of plant glycosides, achieving the putative identification of 274 glycosides in maize using untargeted LC–HRMS/MS [43]. Although cosine similarity thresholds reported in the literature vary widely (e.g., COSMIC ≥ 0.65), we adopted a ≥80% mzCloud Best Match threshold to represent high-confidence spectral agreement under Orbitrap HCD conditions. At this level, fragmentation overlap is more stringent and reduces false positives arising from partial spectral similarity or shared neutral losses. Nevertheless, cosine-based scoring remains insensitive to certain diagnostic ions and cannot fully resolve stereochemical or some regioisomeric differences; therefore, ≥80% BM should be interpreted as a conservative spectral-confidence filter rather than definitive structural proof. In the present work, husk and leaf metabolomes were compared using complementary untargeted acquisition strategies and stringent library-matching criteria, enabling broad coverage and high-confidence putative identifications.

## 5. Conclusions

Low-energy fragmentation (10/50/80% NCE) proved valuable for filtering in-source fragments and improving MS^1^ fragment grouping compared with conventional settings. ddMS2 and AcquireX™ Deep Scan delivered the largest number of identifications, with AcquireX particularly effective at capturing low-abundance features through iterative precursor sampling. ddMS3 and Real-Time Library Search provided the most reliable annotations, yielding higher mzCloud best match scores and enabling structural clarification of isomeric compounds. However, library-based matching still leaves some ambiguity (e.g., stereoisomers); therefore, identifications should be interpreted at MSI Level 2 even when MS^3^ improves confidence for positional isomers. Overall, across all workflows, 472 compounds in *Plantago lanceolata* leaves and 233 in *Plantago ovata* husk were putatively identified. These results underscore the value of low-energy fragmentation and MS^3^-based workflows for confident annotation in complex plant matrices. Continued expansion of plant-specific spectral libraries, together with improved computational annotation tools, will be essential to further increase annotation depth and reliability in future metabolomics studies.

## Figures and Tables

**Table 1:** Overview of compound detection metrics across four acquisition workflows for *P. lanceolata* leaves.

Acquisition Method	Polarity	MS Scan Depth	Features with MS^2^	BM ≥ 60 (per Polarity)	BM ≥ 60 (Unique Compounds)	BM ≥ 80 (Unique Compounds)
ddMS2	pos	2	1284	379	620	398
	neg	2	1134	323		
ddMS3	pos	3	563	220	390	308
	neg	3	428	216		
AcquireX™ Deep Scan	pos	3	7827	735	919	453
	neg ^1^	3	1029	233		
Real-Time Library Search	pos	3	795	322	556	406
	neg	3	790	299		

^1^ Negative mode cycled only for 1 time. All values represent results from three technical replicates processed jointly. “BM ≥ 60 (unique compounds)” counts merged non-redundant IDs across polarities.

## Data Availability

All data supporting the findings of this study are fully available within the article. In addition, the Supplementary Material provides further details on method details and putatively identified compound list. Raw data will be made available by the corresponding author upon reasonable request.

## Supplementary Materials

The following supporting information can be downloaded at: https://www.mdpi.com/article/10.3390/metabo16010042/s1, Figure S1: Acquisition method parameters for ddMS^2^ and ddMS^3^ data-dependent acquisition; Figure S2: maxID workflow for untargeted compound detection and identification in Compound Discoverer™ 3.4; Figure S3: Extracted ion chromatograms demonstrating co-elution and misannotation of an in-source fragment; Table S1: Representative MS^1^, MS^2^, and MS^3^ examples illustrating refinement of compound annotations by MS^3^; Table S2: List of compounds with plant part, acquisition method, match score, and retention time.

## Abbreviations

The following abbreviations are used in this manuscript:

AGC	Automatic gain control
BM	Best match
COSMIC	Confidence of small molecule identifications
CSI	Compound structure identification
DDA	Data-dependent acquisition
ddMS2	Data-dependent MS^2^ acquisition
ddMS3	Data-dependent MS^3^ acquisition
ESI	Electrospray ionization
ISF	In-source fragmentation
ISFs	In-source fragments
HCD	Higher-energy collisional dissociation
HRMS	High-resolution mass spectrometry
ISCID	In-source collision-induced dissociation
MSI	Metabolomics standards initiative
NCE	Normalized collision energy
NMR	Nuclear magnetic resonance
OT	Orbitrap
RDA	retro-Diels–Alder
RF	Radio frequency
RTLS	Real-time library search
TopN	Number of precursors per cycle
UHPLC	Ultra-high-performance liquid chromatography
UV	Ultraviolet
