# Supplementary material for: Detecting and Grouping In-Source Fragments with Low-Energy Stepped HCD, Together with MS3, Increases Identification Confidence in Untargeted LC–Orbitrap Metabolomics of Plantago lanceolata Leaves and P. ovata Husk
Source: Metabolites. 2026 Jan 2;16(1):42. doi: 10.3390/metabo16010042 (PMC12843812; doi:10.3390/metabo16010042)
Supplement: Supplementary file 1 [file metabolites-16-00042-s001.zip › metabolites-4014971-supplementary.pdf]

Supplementary Material

Detecting and grouping in-source fragments with low-energy stepped HCD, together with MS^3, increases identification confidence in untargeted LC–Orbitrap metabolomics of *Plantago lanceolata* leaves and *P. ovata* husk

a.

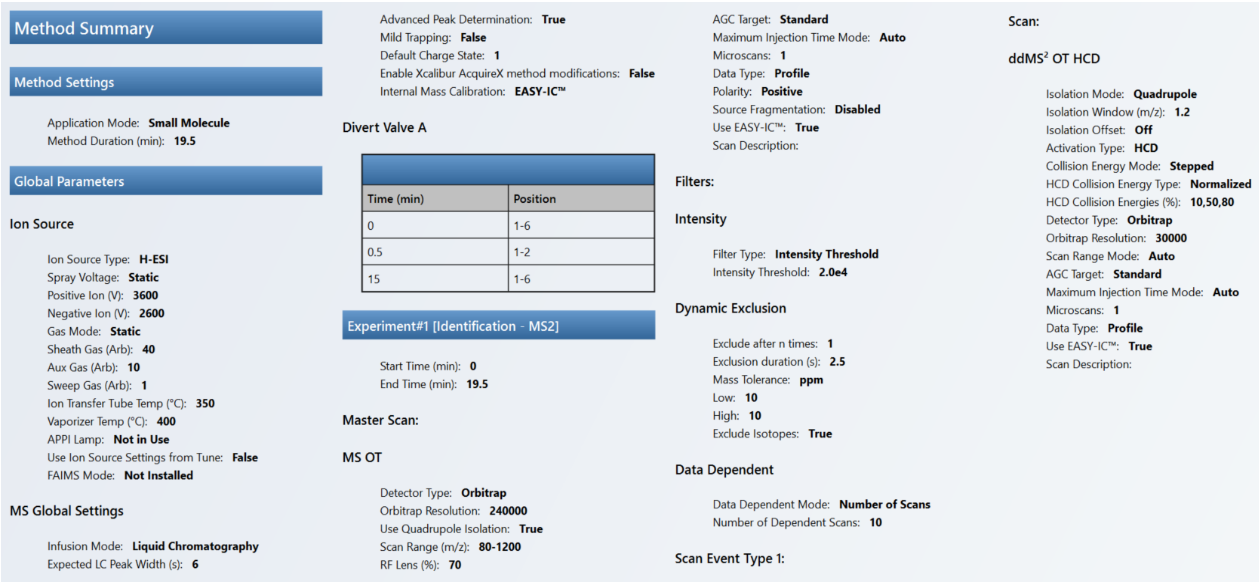

Method Summary

Method Settings

Application Mode: **Small Molecule**
Method Duration (min): **19.5**

Global Parameters

Ion Source

Ion Source Type: **H-ESI**
Spray Voltage: **Static**
Positive Ion (V): **3600**
Negative Ion (V): **2600**
Gas Mode: **Static**
Sheath Gas (Arb): **40**
Aux Gas (Arb): **10**
Sweep Gas (Arb): **1**
Ion Transfer Tube Temp (°C): **350**
Vaporizer Temp (°C): **400**
APPI Lamp: **Not in Use**
Use Ion Source Settings from Tune: **False**
FAIMS Mode: **Not Installed**

MS Global Settings

Infusion Mode: **Liquid Chromatography**
Expected LC Peak Width (s): **6**
Advanced Peak Determination: **True**
Mild Trapping: **False**
Default Charge State: **1**
Enable Xcalibur AcquireX method modifications: **False**
Internal Mass Calibration: **EASY-IC™**

Divert Valve A

Time (min)	Position
0	1-6
0.5	1-2
15	1-6

Experiment#1 [Identification - MS2]

Start Time (min): **0**
End Time (min): **19.5**

Master Scan:

MS OT

Detector Type: **Orbitrap**
Orbitrap Resolution: **240000**
Use Quadrupole Isolation: **True**
Scan Range (m/z): **80-1200**
RF Lens (%): **70**
AGC Target: **Standard**
Maximum Injection Time Mode: **Auto**
Microscans: **1**
Data Type: **Profile**
Polarity: **Positive**
Source Fragmentation: **Disabled**
Use EASY-IC™: **True**
Scan Description:

Filters:

Intensity

Filter Type: **Intensity Threshold**
Intensity Threshold: **2.0e4**

Dynamic Exclusion

Exclude after n times: **1**
Exclusion duration (s): **2.5**
Mass Tolerance: **ppm**
Low: **10**
High: **10**
Exclude Isotopes: **True**

Data Dependent

Data Dependent Mode: **Number of Scans**
Number of Dependent Scans: **10**

Scan Event Type 1:

Scan:

ddMS^2 OT HCD

Isolation Mode: **Quadrupole**
Isolation Window (m/z): **1.2**
Isolation Offset: **Off**
Activation Type: **HCD**
Collision Energy Mode: **Stepped**
HCD Collision Energy Type: **Normalized**
HCD Collision Energies (%): **10,50,80**
Detector Type: **Orbitrap**
Orbitrap Resolution: **30000**
Scan Range Mode: **Auto**
AGC Target: **Standard**
Maximum Injection Time Mode: **Auto**
Microscans: **1**
Data Type: **Profile**
Use EASY-IC™: **True**
Scan Description:

b.

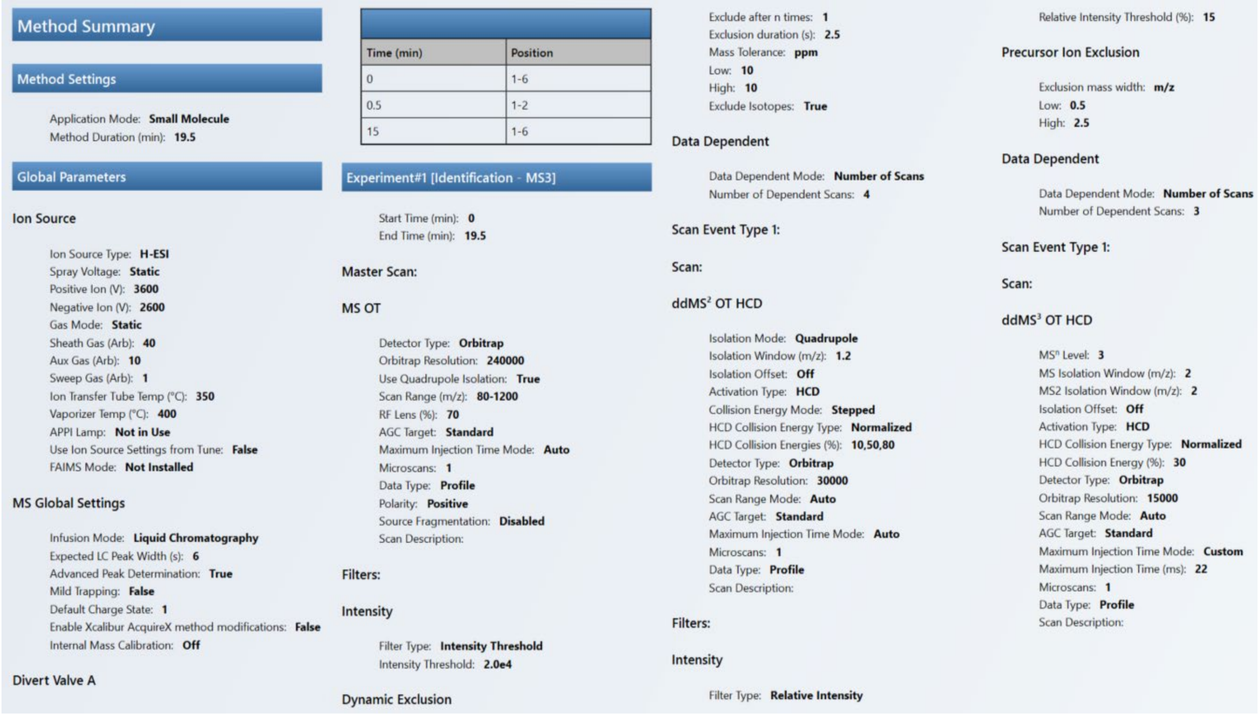

Method Summary

Method Settings

Application Mode: **Small Molecule**
Method Duration (min): **19.5**

Global Parameters

Ion Source

Ion Source Type: **H-ESI**
Spray Voltage: **Static**
Positive Ion (V): **3600**
Negative Ion (V): **2600**
Gas Mode: **Static**
Sheath Gas (Arb): **40**
Aux Gas (Arb): **10**
Sweep Gas (Arb): **1**
Ion Transfer Tube Temp (°C): **350**
Vaporizer Temp (°C): **400**
APPI Lamp: **Not in Use**
Use Ion Source Settings from Tune: **False**
FAIMS Mode: **Not Installed**

MS Global Settings

Infusion Mode: **Liquid Chromatography**
Expected LC Peak Width (s): **6**
Advanced Peak Determination: **True**
Mild Trapping: **False**
Default Charge State: **1**
Enable Xcalibur AcquireX method modifications: **False**
Internal Mass Calibration: **Off**

Divert Valve A

Time (min)	Position
0	1-6
0.5	1-2
15	1-6

Experiment#1 [Identification - MS3]

Start Time (min): **0**
End Time (min): **19.5**

Master Scan:

MS OT

Detector Type: **Orbitrap**
Orbitrap Resolution: **240000**
Use Quadrupole Isolation: **True**
Scan Range (m/z): **80-1200**
RF Lens (%): **70**
AGC Target: **Standard**
Maximum Injection Time Mode: **Auto**
Microscans: **1**
Data Type: **Profile**
Polarity: **Positive**
Source Fragmentation: **Disabled**
Scan Description:

Filters:

Intensity

Filter Type: **Intensity Threshold**
Intensity Threshold: **2.0e4**

Dynamic Exclusion

Exclude after n times: **1**
Exclusion duration (s): **2.5**
Mass Tolerance: **ppm**
Low: **10**
High: **10**
Exclude Isotopes: **True**

Data Dependent

Data Dependent Mode: **Number of Scans**
Number of Dependent Scans: **4**

Scan Event Type 1:

Scan:

ddMS^2 OT HCD

Isolation Mode: **Quadrupole**
Isolation Window (m/z): **1.2**
Isolation Offset: **Off**
Activation Type: **HCD**
Collision Energy Mode: **Stepped**
HCD Collision Energy Type: **Normalized**
HCD Collision Energies (%): **10,50,80**
Detector Type: **Orbitrap**
Orbitrap Resolution: **30000**
Scan Range Mode: **Auto**
AGC Target: **Standard**
Maximum Injection Time Mode: **Auto**
Microscans: **1**
Data Type: **Profile**
Scan Description:

Filters:

Intensity

Filter Type: **Relative Intensity**
Relative Intensity Threshold (%): **15**

Precursor Ion Exclusion

Exclusion mass width: **m/z**
Low: **0.5**
High: **2.5**

Data Dependent

Data Dependent Mode: **Number of Scans**
Number of Dependent Scans: **3**

Scan Event Type 1:

Scan:

ddMS^3 OT HCD

MS^n Level: **3**
MS Isolation Window (m/z): **2**
MS2 Isolation Window (m/z): **2**
Isolation Offset: **Off**
Activation Type: **HCD**
HCD Collision Energy Type: **Normalized**
HCD Collision Energy (%): **30**
Detector Type: **Orbitrap**
Orbitrap Resolution: **15000**
Scan Range Mode: **Auto**
AGC Target: **Standard**
Maximum Injection Time Mode: **Custom**
Maximum Injection Time (ms): **22**
Microscans: **1**
Data Type: **Profile**
Scan Description:

Figure S1 Acquisition Method Parameters for Data-Dependent Acquisition (DDA) MS² (ddMS2) and ddMS3 data. (a) panel illustrates the acquisition method parameters utilized for acquiring ddMS2 data. (b) panel displays the acquisition method parameters for ddMS3 data, which were consistently employed across ddMS3, real-time library search, and AcquireX deep scan methods.

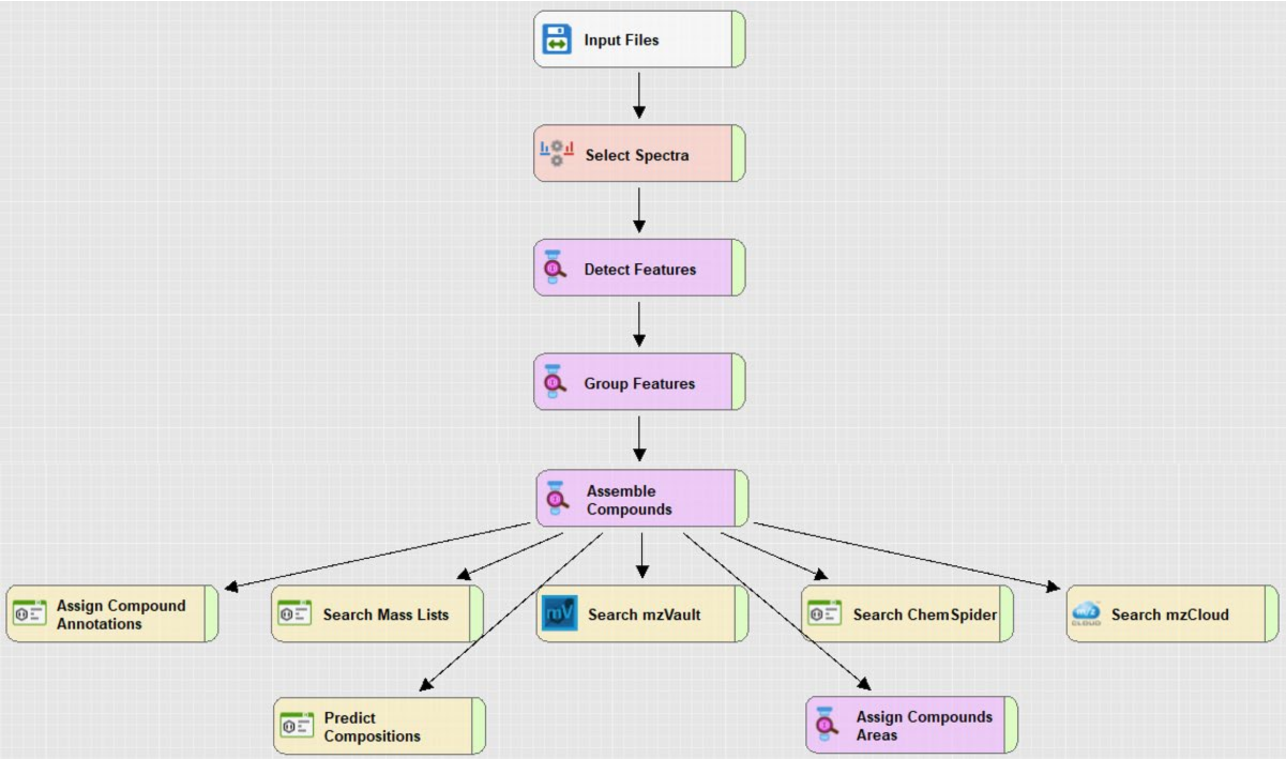

Figure S2 Workflow for Untargeted Compound Detection and Identification (“maxID” Workflow) in Compound Discoverer™ 3.4 is set up to extract and annotate every detectable feature—down to very low abundances—in a single LC–MS run. Raw files are processed through Select Spectra, Detect Features, and Group Features to generate aligned chromatographic features across samples. The Assemble Compounds node then merges related features into unified compound entries using accurate mass and retention time proximity, including adduct and isotope grouping, and assigns in-source fragments as MS^1 fragments when they co-eluted with the parent ion and are supported by MS^2-based relationships. Raw data are peak-picked with minimal intensity thresholds and deconvoluted without filters, then accurate-mass and isotope fitting yield molecular formula candidates. MS^2 spectra are matched against the mzCloud library for high-confidence identifications, while parallel exact-mass searches against in-house lists and ChemSpider provide formula-only hits. A blanket similarity search of all MS^2 scans fills gaps for unmatched spectra. Finally, all precursor and fragment ions are merged into unified compound entries, maximizing coverage at the expense of longer run times.

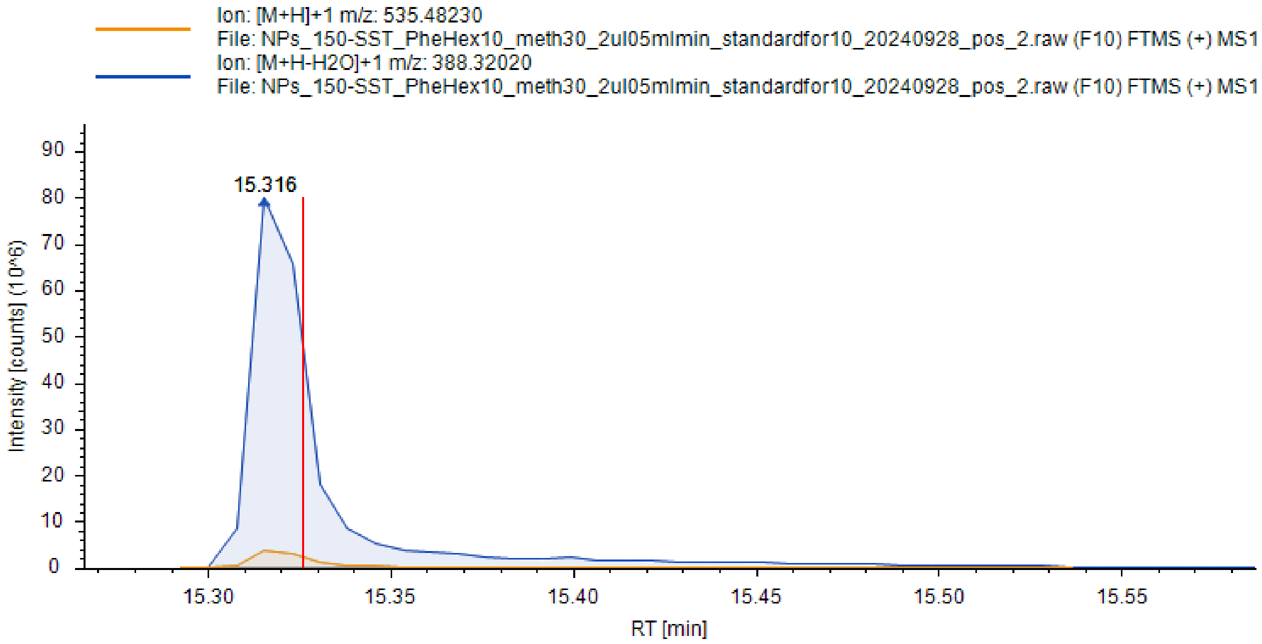

Figure S3 Extracted ion chromatograms (EICs) for m/z 535.4823 and m/z 388.3202 demonstrating co-elution. The signal at m/z 388.3202 is seen as initially misannotated $[M+H-H_20]^+$ ion due to usage of only higher-energy HCD fragmentation data (30/50/80% NCE), which did not reveal the lower-energy MS^1 fragment.

Table S1 Representative MS¹, MS² and MS³ examples illustrating how MS³ refines compound annotations across different compounds.

<table>
<tr><th>MS¹</th><th>MS² (initial annotation)</th><th>MS³ (updated annotation)</th></tr>
<tr>
<td>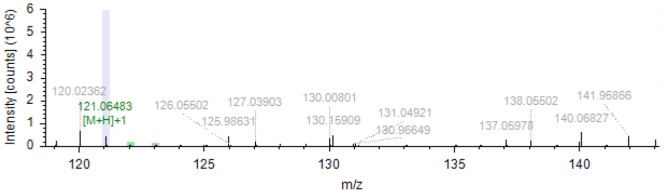</td>
<td>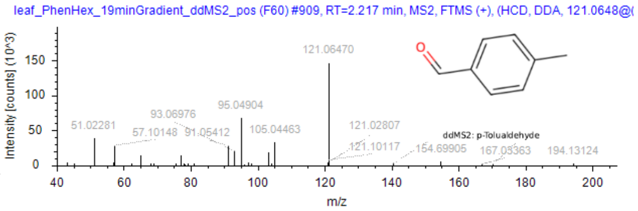</td>
<td>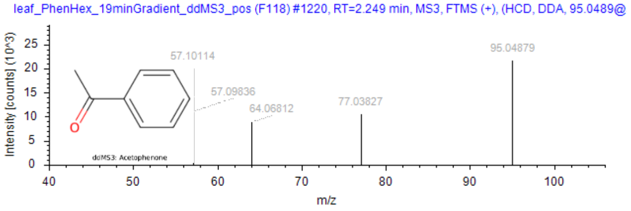</td>
</tr>
<tr>
<td>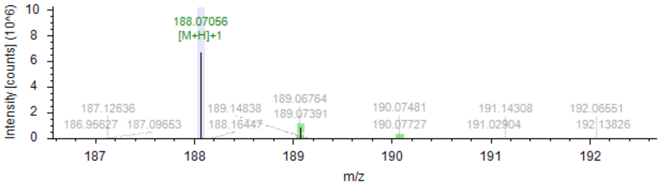</td>
<td>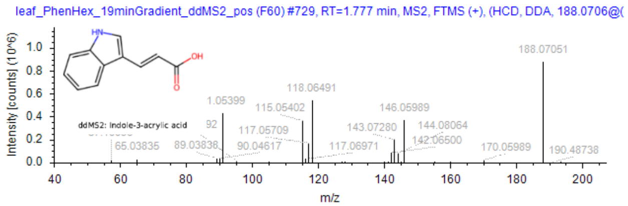</td>
<td>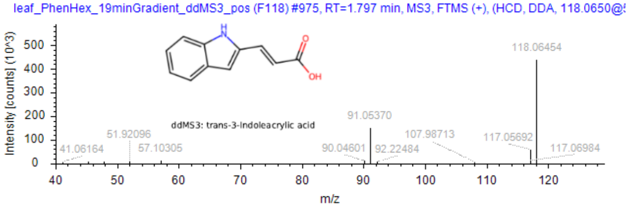</td>
</tr>
<tr>
<td>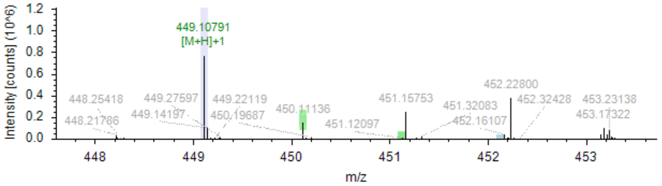</td>
<td>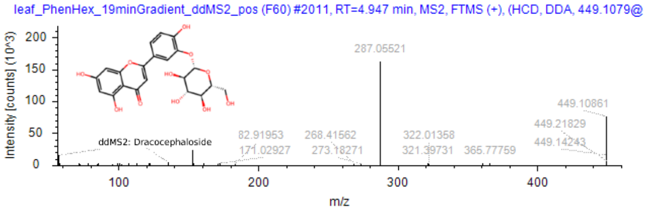</td>
<td>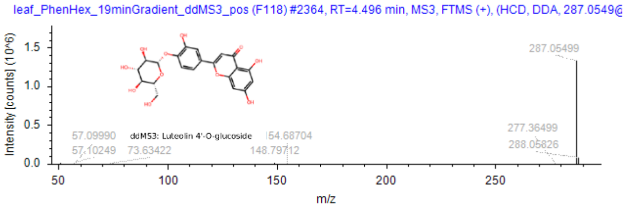</td>
</tr>
<tr>
<td>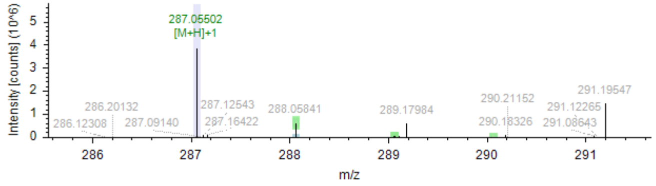</td>
<td>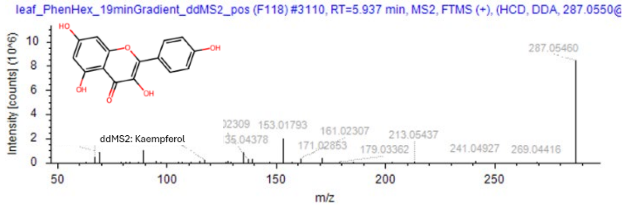</td>
<td>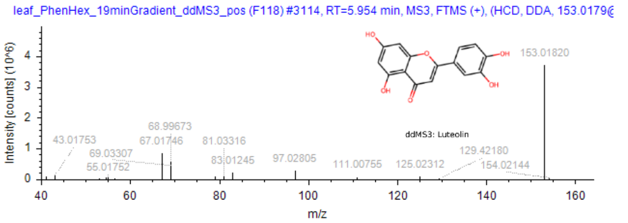</td>
</tr>
</table>

Table S2 List of compounds with plant part, acquisition methods with their according match scores, and retention times.

Compound	Plant Part	Acquisition Method	mzCloud Best Match	Retention time, min
(+/-)9,10-dihydroxy-12Z-octadecenoic acid	P. lanceolata leaf	ddMS3, RTLS, AcquireX Deep Scan, ddMS2	98.3, 98.4, 97.9, 98.3	9.267
(11E,15Z)-9,10,13-Trihydroxy-11,15-octadecadienoic acid	P. lanceolata leaf	ddMS3, RTLS, AcquireX Deep Scan, ddMS2	89.7, 86.7, 93.0, 75.4	6.766
(12Z,15Z)-9,10,11-Trihydroxy-12,15-octadecadienoic acid	P. lanceolata leaf	ddMS3, RTLS, AcquireX Deep Scan, ddMS2	95.5, 98.4, 90.6, 94.7	7.682
(15Z)-9,12,13-Trihydroxy-15-octadecenoic acid	P. lanceolata leaf	ddMS3, RTLS, AcquireX Deep Scan	98.8, 95.1, 88.4	7.15
(1R,9R)-N-(4-Acetylphenyl)-5-{[(4-methoxyphenyl)sulfonyl]amino}-6-oxo-7,11-diazatricyclo[7.3.1.02,7]trideca-2,4-diene-11-carboxamide	P. lanceolata leaf	ddMS3, RTLS, AcquireX Deep Scan, ddMS2	95.5, 90.9, 89.5, 95.1	1.353
(1S,4aS,5S)-1,4a-dimethyl-6-methylidene-5-[(E)-3-oxobut-1-enyl]-3,4,5,7,8,8a-hexahydro-2H-naphthalene-1-carboxylic acid	P. lanceolata leaf	ddMS3, RTLS, AcquireX Deep Scan, ddMS2	76.9, 96.6, 72.7, 95.7	7.764
(2R,3R,4S,5S,6R)-2-(3-methylbutoxy)-6-[[(2S,3R,4S,5R)-3,4,5-trihydroxyoxan-2-yl]oxymethyl]oxane-3,4,5-triol	P. lanceolata leaf	ddMS3, RTLS, AcquireX Deep Scan, ddMS2	90.0, 85.7, 73.2, 87.9	3.519
(2S)-pyrrolidinium-2-carboxylate	Both	ddMS3, RTLS, AcquireX Deep Scan, ddMS2	99.8, 99.8, 99.7, 99.9	0.455
(2S,3R,4S,5R)-2-{[(2R,3R,4S,5S,6R)-4,5-dihydroxy-6-(hydroxymethyl)-2-(2-phenylethoxy)oxan-3-yl]oxy}oxane-3,4,5-triol	P. lanceolata leaf	ddMS3, RTLS, AcquireX Deep Scan, ddMS2	93.1, 92.7, 76.9, 89.1	3.805
(2S,3R,4S,5S,6R)-2-[(6E)-3,10-dihydroxy-2,6,10-trimethyldodeca-6,11-dien-2-yl]oxy-6-(hydroxymethyl)oxane-3,4,5-triol	P. lanceolata leaf	ddMS3, RTLS, AcquireX Deep Scan	99.6, 99.7, 99.6	5.178
(2S,3S,4S,5R,6R)-3,4,5-trihydroxy-6-[2-hydroxy-3-[(9Z,12Z)-octadeca-9,12-dienoyl]oxypropoxy]oxane-2-carboxylic acid	P. lanceolata leaf	ddMS3, RTLS, AcquireX Deep Scan, ddMS2	99.4, 99.5, 98.6, 99.2	8.328
(2S,3S,4S,5R,6S)-3,4,5-trihydroxy-6-[5-hydroxy-2-(2-hydroxyphenyl)-6-methoxy-4-oxochromen-7-yl]oxyoxane-2-carboxylic acid	P. lanceolata leaf	ddMS3, RTLS, AcquireX Deep Scan, ddMS2	87.1, 87.8, 88.7, 87.4	5.099
(2S,5aS,8aR)-6-Benzyl-1-methyl-2-[3-(4-morpholinyl)-3-oxopropyl]octahydropyrrolo[3,2-E][1,4]diazepin-5(2H)-one	P. lanceolata leaf	ddMS3, RTLS, AcquireX Deep Scan, ddMS2	99.6, 95.5, 98.7, 99.2	6.367
(3R,4S,5S,6R)-2-[4-(2-hydroxyethyl)phenoxy]-6-(hydroxymethyl)oxane-3,4,5-triol	Both	ddMS3, RTLS, AcquireX Deep Scan, ddMS2	98.7, 97.2, 99.5, 99.3	2.183
(4E,6E)-2,7-dimethyl-8-[(2R,3R,4S,5S,6R)-3,4,5-trihydroxy-6-(hydroxymethyl)oxan-2-yl]oxyocta-4,6-dienoic acid	Both	ddMS3, RTLS, AcquireX Deep Scan, ddMS2	90.8, 95.0, 74.4, 65.7	4.403

(4aR)-5-hydroxy-6-methoxy-1,1-dimethyl-7-propan-2-yl-2,3,4,9,10,10a-hexahydrophenanthrene-4a-carboxylic acid	P. lanceolata leaf	ddMS3, RTLS, AcquireX Deep Scan, ddMS2	95.1, 94.4, 94.1, 95.3	11.529
(4aS,7aR)-3-(3-Fluorophenyl)-2,4-dioxo-N-propyl-1-(2-thienylmethyl)octahydro-5H-pyrrolo[3,2-d]pyrimidine-5-carboxamide	P. lanceolata leaf	ddMS3, RTLS, AcquireX Deep Scan, ddMS2	99.1, 96.3, 99.0, 98.1	3.784
(5E)-3,4,9-trihydroxy-2-propyl-2,3,4,7,8,9-hexahydrooxecin-10-one	Both	ddMS3, RTLS, AcquireX Deep Scan, ddMS2	98.2, 97.7, 95.9, 97.9	5.536
(5E,9E)-4,8-dihydroxy-5,9,13,14-tetramethyl-1-oxacyclotetradeca-5,9-dien-2-one	P. lanceolata leaf	ddMS3, RTLS, AcquireX Deep Scan, ddMS2	86.4, 88.2, 76.2, 88.9	5.925
(6E,8E)-5,10-dioxooctadeca-6,8-dienoic acid	P. lanceolata leaf	ddMS3, RTLS, AcquireX Deep Scan, ddMS2	82.4, 81.7, 83.7, 80.1	8.022
(9R,10E,12Z,15Z)-9-hydroxyoctadeca-10,12,15-trienoic acid	Both	ddMS3, RTLS, AcquireX Deep Scan	96.2, 96.1, 91.4	10.103
(9Z,12Z)-7,8,16-trihydroxyoctadeca-9,12-dienoic acid	P. lanceolata leaf	ddMS3, RTLS, AcquireX Deep Scan, ddMS2	97.3, 96.6, 95.3, 96.6	9.086
(E)-8,9,10-trihydroxyoctadec-6-enoic acid	Both	ddMS3, RTLS, AcquireX Deep Scan	98.6, 99.0, 85.1	7.143
(R)-Bitalin A	P. lanceolata leaf	ddMS3, RTLS, AcquireX Deep Scan, ddMS2	79.4, 81.4, 70.7, 80.1	4.157
(Rac)-Idroxioleic acid	Both	ddMS3, RTLS, AcquireX Deep Scan, ddMS2	88.2, 86.3, 89.2, 86.9	8.347
1,4:3,6-Dianhydro-2-(benzylamino)-5-[4-(cyclohexylmethyl)-1H-1,2,3-triazol-1-yl]-2,5-dideoxy-L-iditol	P. lanceolata leaf	ddMS3, RTLS, AcquireX Deep Scan, ddMS2	99.5, 98.2, 99.6, 99.5	7.458
1,4:3,6-Dianhydro-2-{[4-(4-biphenylyl)-2-pyrimidinyl]amino}-2,5-dideoxy-5-[(2-thienylcarbonyl)amino]-L-iditol	P. lanceolata leaf	ddMS3, RTLS, AcquireX Deep Scan, ddMS2	92.3, 99.3, 91.8, 92.1	3.561
1-(4-benzylpiperazino)-2-(pyridin-2-ylamino)propan-1-one	P. lanceolata leaf	ddMS3, RTLS, AcquireX Deep Scan, ddMS2	99.4, 76.2, 95.2, 98.2	8.184
1-O-Cinnamoylglucose	Both	ddMS3, RTLS, AcquireX Deep Scan, ddMS2	82.9, 85.6, 63.9, 86.0	0.766
1-Octen-3-yl primeveroside	P. lanceolata leaf	ddMS3, RTLS, AcquireX Deep Scan	87.4, 87.4, 92.1	5.388
11-(2-Hydroxy-3,4-dimethyl-5-oxofuran-2-yl)undecanoic acid	P. lanceolata leaf	ddMS3, RTLS, AcquireX Deep Scan, ddMS2	95.3, 94.7, 85.6, 84.2	8.416
11-(2-Methoxy-3,4-dimethyl-5-oxofuran-2-yl)undecanoic acid	P. lanceolata leaf	ddMS3, RTLS, AcquireX Deep Scan, ddMS2	79.6, 67.2, 81.1, 97.2	8.03
12-Oxo phytodienoic acid	Both	ddMS3, RTLS, AcquireX Deep Scan, ddMS2	97.1, 97.3, 90.3, 96.6	6.773
13(S)-HOTrE	Both	ddMS3, RTLS, AcquireX Deep Scan, ddMS2	99.2, 99.2, 99.0, 99.3	7.143

19-Norandrostenedione	P. lanceolata leaf	ddMS3, RTLS, AcquireX Deep Scan, ddMS2	78.9, 80.3, 78.6, 78.9	8.197
2',5,6',7-tetrahydroxyflavone	P. lanceolata leaf	ddMS3, RTLS, AcquireX Deep Scan, ddMS2	86.4, 96.4, 85.4, 81.5	5.399
2,3,4,5,6-pentahydroxyhexyl (9Z,12Z)-octadeca-9,12-dienoate	P. lanceolata leaf	ddMS3, RTLS, AcquireX Deep Scan, ddMS2	99.8, 99.7, 99.8, 99.8	10.715
2,3,4,9-Tetrahydro-1H-β-carboline-3-carboxylic acid	Both	ddMS3, RTLS, AcquireX Deep Scan, ddMS2	93.5, 94.9, 93.3, 90.9	2.688
2,3-Dinor prostaglandin E1	P. lanceolata leaf	ddMS3, RTLS, AcquireX Deep Scan, ddMS2	82.1, 82.8, 80.5, 76.7	6.531
2,3-Dinor-11β-prostaglandin F2α	P. lanceolata leaf	ddMS3, RTLS, AcquireX Deep Scan	96.0, 85.7, 64.4	7.759
2-(3,4-Dihydroxyphenyl)ethyl 3-O-(6-deoxy-β-L-mannopyranosyl)-6-O-[(2E)-3-(3,4-dihydroxyphenyl)-2-propenoyl]-β-D-glucopyranoside	Both	ddMS3, RTLS, AcquireX Deep Scan, ddMS2	89.2, 91.2, 91.2, 90.4	4.533
2-(hydroxymethyl)-6-[(E)-4-(1,2,4-trihydroxy-2,6,6-trimethylcyclohexyl)but-3-en-2-yl]oxyoxane-3,4,5-triol	P. lanceolata leaf	ddMS3, RTLS, AcquireX Deep Scan, ddMS2	97.5, 96.1, 98.5, 97.2	2.796
2-Amino-1,3,4-octadecanetriol	Both	ddMS3, RTLS, AcquireX Deep Scan	90.5, 91.2, 91.2	7.701
2-Hydroxycaproic acid	P. lanceolata leaf	ddMS3, RTLS, AcquireX Deep Scan, ddMS2	98.5, 93.4, 98.6, 98.6	2.962
2-Hydroxysebacic acid	P. lanceolata leaf	ddMS3, RTLS, AcquireX Deep Scan, ddMS2	92.4, 95.3, 88.6, 93.1	4.411
2-Isopropylmalic acid	Both	ddMS3, RTLS, AcquireX Deep Scan, ddMS2	92.8, 83.0, 94.3, 92.6	2.271
2-Methoxy-N-({(2R,4S,5R)-5-[3-(4-methoxyphenyl)-1-methyl-1H-pyrazol-5-yl]-1-azabicyclo[2.2.2]oct-2-yl}methyl)acetamide	P. lanceolata leaf	ddMS3, RTLS, AcquireX Deep Scan, ddMS2	99.1, 99.1, 98.8, 99.1	6.729
2-[3-Oxo-2-[4-[3,4,5-trihydroxy-6-(hydroxymethyl)oxan-2-yl]oxypentyl]cyclopentyl]acetic acid	P. lanceolata leaf	ddMS3, RTLS, AcquireX Deep Scan, ddMS2	93.9, 94.5, 76.6, 95.5	5.357
2-hydroxy-5-[3,4,5-trihydroxy-6-(hydroxymethyl)oxan-2-yl]oxybenzoic acid	Both	ddMS3, RTLS, AcquireX Deep Scan, ddMS2	97.7, 98.8, 98.1, 96.4	1.171
3-Hexadec-15-en-7-ynyl-4-hydroxy-5-methyloxolan-2-one	P. lanceolata leaf	ddMS3, RTLS, AcquireX Deep Scan	93.9, 92.5, 93.8	14.132
3-Hydroxystigmast-5-en-7-one	P. lanceolata leaf	ddMS3, RTLS, AcquireX Deep Scan, ddMS2	95.6, 87.7, 88.2, 94.5	14.38
3-Methoxy-4-[3,4,5-trihydroxy-6-(hydroxymethyl)oxan-2-yl]oxybenzoic acid	P. lanceolata leaf	ddMS3, RTLS, AcquireX Deep Scan, ddMS2	96.0, 94.6, 98.3, 95.9	1.263
3-Methylidene-7-propan-2-yldodecane-1,2-diol	P. lanceolata leaf	ddMS3, RTLS, AcquireX Deep Scan, ddMS2	98.3, 99.8, 98.6, 99.1	11.182

3-[2-[3,4,5-Trihydroxy-6-(hydroxymethyl)oxan-2-yl]oxyphenyl]prop-2-enoic acid	Both	ddMS3, RTLS, AcquireX Deep Scan, ddMS2	97.3, 92.5, 99.3, 95.8	2.897
3-[3-(beta-D-Glucopyranosyloxy)-2-methoxyphenyl]propanoic acid	P. lanceolata leaf	ddMS3, RTLS, AcquireX Deep Scan, ddMS2	96.2, 98.2, 96.9, 98.0	2.836
3-heptyl-3,6-dihydro-1H-furo[3,4-c]furan-4-one	P. lanceolata leaf	ddMS3, RTLS, AcquireX Deep Scan, ddMS2	80.3, 78.9, 63.3, 77.9	6.064
4-(3-Hydroxybutyl)phenyl β-D-glucopyranoside	P. lanceolata leaf	ddMS3, RTLS, AcquireX Deep Scan, ddMS2	93.4, 93.2, 90.6, 94.8	5.009
4-(beta-D-Glucopyranosyloxy)phenylacetic acid	P. lanceolata leaf	ddMS3, RTLS, AcquireX Deep Scan, ddMS2	90.8, 87.1, 83.4, 82.8	1.506
4-Acetamidobutanoic acid	P. lanceolata leaf	ddMS3, RTLS, AcquireX Deep Scan, ddMS2	83.5, 90.7, 82.4, 86.5	1.59
4-Hydroxybenzaldehyde	Both	ddMS3, RTLS, AcquireX Deep Scan, ddMS2	98.2, 98.5, 61.2, 98.1	3.231
4-Hydroxycinnamic acid	P. lanceolata leaf	ddMS3, RTLS, AcquireX Deep Scan, ddMS2	99.0, 87.2, 77.4, 71.9	3.949
4-Indolecarbaldehyde	P. lanceolata leaf	ddMS3, RTLS, AcquireX Deep Scan, ddMS2	98.8, 80.2, 98.4, 98.7	4.698
4-Methyl-2-(2-methylpropanoyloxy)-3-undecanoyloxypentanoic acid	P. lanceolata leaf	ddMS3, RTLS, AcquireX Deep Scan, ddMS2	99.6, 99.5, 99.6, 99.5	8.153
4-Methylquinoline	P. lanceolata leaf	ddMS3, RTLS, AcquireX Deep Scan, ddMS2	83.0, 91.3, 74.9, 76.4	2.686
4-O-beta-D-glucosyl-4-coumaric acid	P. lanceolata leaf	ddMS3, RTLS, AcquireX Deep Scan, ddMS2	97.4, 97.8, 97.4, 97.7	2.893
4-hydroxy-3-[(2S,3R,4S,5S,6R)-3,4,5-trihydroxy-6-(3-methylbutanoyloxymethyl)oxan-2-yl]oxybenzoic acid	P. lanceolata leaf	ddMS3, RTLS, AcquireX Deep Scan, ddMS2	74.6, 94.3, 81.8, 92.6	4.053
5,5-dimethyl-4-[(E)-3-methyl-7-[(2R,3R,4S,5S,6R)-3,4,5-trihydroxy-6-(hydroxymethyl)oxan-2-yl]oxyoct-3-enyl]oxolan-2-one	P. lanceolata leaf	ddMS3, RTLS, AcquireX Deep Scan, ddMS2	97.0, 97.3, 92.3, 98.1	5.122
5-p-Coumaroylquinic acid, (Z)-	P. lanceolata leaf	ddMS3, RTLS, AcquireX Deep Scan, ddMS2	95.9, 98.0, 86.6, 93.0	3.933
6-hydroxy-4,4,7a-trimethyl-6,7-dihydro-5H-1-benzofuran-2-one	P. lanceolata leaf	ddMS3, RTLS, AcquireX Deep Scan, ddMS2	85.5, 86.1, 85.7, 88.2	4.521
7-[2-(1-hydroxyhexyl)-3,6-dihydro-2H-pyran-6-yl]heptanoic acid	P. lanceolata leaf	ddMS3, RTLS, AcquireX Deep Scan, ddMS2	90.8, 74.6, 90.3, 88.8	8.757
8,9-Dimethoxy-2-(2-phenyldiazenyl)-3-(2-thienyl)-5,6-dihydropyrrolo[2,1-a]isoquinoline-1-carbonitrile	P. lanceolata leaf	ddMS3, RTLS, AcquireX Deep Scan	98.7, 95.9, 64.1	2.722
8-Hydroxyquinoline	P. lanceolata leaf	ddMS3, RTLS, AcquireX Deep Scan, ddMS2	80.5, 93.2, 80.4, 78.9	4.696

9(Z),11(E)-Conjugated linoleic acid	P. lanceolata leaf	ddMS3, RTLS, AcquireX Deep Scan, ddMS2	100.0, 99.8, 99.9, 99.9	10.714
9-(2,3-dihydroxypropoxy)-9-oxononanoic acid	Both	ddMS3, RTLS, AcquireX Deep Scan, ddMS2	94.1, 95.5, 66.3, 94.7	13.58
9-oxooctadeca-10,12-dienoic acid	Both	ddMS3, RTLS, AcquireX Deep Scan, ddMS2	96.9, 97.8, 98.0, 97.2	10.938
ADB-PINACA	P. lanceolata leaf	ddMS3, RTLS, AcquireX Deep Scan, ddMS2	99.4, 99.2, 96.4, 99.5	5.418
AWTYKUNFPBFFHC-UHFFFAOYSA-N	P. lanceolata leaf	ddMS3, RTLS, AcquireX Deep Scan	98.7, 98.6, 98.4	3.931
Adenine	Both	ddMS3, RTLS, AcquireX Deep Scan, ddMS2	99.5, 99.1, 98.2, 98.8	0.47
Agnuside	P. lanceolata leaf	ddMS3, RTLS, AcquireX Deep Scan, ddMS2	99.3, 99.6, 98.5, 99.6	4.127
Ancymidol	P. lanceolata leaf	ddMS3, RTLS, AcquireX Deep Scan, ddMS2	85.0, 86.3, 88.2, 83.3	6.15
Apigenin	P. lanceolata leaf	ddMS3, RTLS, AcquireX Deep Scan, ddMS2	99.5, 99.3, 99.4, 99.4	6.63
Arachidoyl glycine	P. lanceolata leaf	ddMS3, RTLS, AcquireX Deep Scan, ddMS2	84.3, 85.5, 80.1, 84.0	13.26
BMOKZWFNXYQOGE-REKXUBECSA-N	P. lanceolata leaf	ddMS3, RTLS, AcquireX Deep Scan, ddMS2	73.8, 72.5, 99.0, 99.0	5.371
BUKBJIBZOCTYLQ-UQTFRHSQSA-N	P. lanceolata leaf	ddMS3, RTLS, AcquireX Deep Scan, ddMS2	92.0, 94.3, 79.5, 89.8	12.949
Baldaccioside	P. lanceolata leaf	ddMS3, RTLS, AcquireX Deep Scan, ddMS2	97.2, 83.8, 84.0, 89.9	5.628
Betaine	P. lanceolata leaf	ddMS3, RTLS, AcquireX Deep Scan, ddMS2	95.7, 95.0, 94.7, 95.0	12.75
Bioside	Both	ddMS3, RTLS, AcquireX Deep Scan, ddMS2	95.1, 94.8, 93.3, 94.7	1.915
CAIKQNWPWSTGHN-CXNFFOGLSA-N	Both	ddMS3, RTLS, AcquireX Deep Scan, ddMS2	98.3, 97.1, 97.8, 99.9	6.773
CB-25	P. lanceolata leaf	ddMS3, RTLS, AcquireX Deep Scan, ddMS2	90.2, 90.6, 80.8, 91.3	13.327
CBJNLOVRAFQEQH-XTAVSMKVSA-N	P. lanceolata leaf	ddMS3, RTLS, AcquireX Deep Scan, ddMS2	99.8, 99.7, 99.8, 99.8	5.169
CCTTLCTYXPWGOMY-JKUWKVMRSA-N	P. lanceolata leaf	ddMS3, RTLS, AcquireX Deep Scan, ddMS2	99.2, 98.3, 97.3, 99.5	4.409
Caffeic acid	Both	ddMS3, RTLS, AcquireX Deep Scan, ddMS2	94.3, 94.1, 94.3, 94.4	4.767

Carbofuranphenol-3-keto	P. lanceolata leaf	ddMS3, RTLS, AcquireX Deep Scan, ddMS2	77.4, 82.5, 68.8, 74.8	2.082
Cassifolioside	Both	ddMS3, RTLS, AcquireX Deep Scan, ddMS2	98.7, 85.1, 96.4, 98.7	4.646
Chlorogenic acid	P. lanceolata leaf	ddMS3, RTLS, AcquireX Deep Scan, ddMS2	99.1, 95.1, 97.8, 98.9	2.782
Citraconic acid	P. lanceolata leaf	ddMS3, RTLS, AcquireX Deep Scan, ddMS2	95.3, 94.2, 93.6, 86.9	0.585
Corchoionoside C	Both	ddMS3, RTLS, AcquireX Deep Scan, ddMS2	99.5, 99.6, 99.1, 99.5	3.405
Corchorifatty acid F	Both	ddMS3, RTLS, AcquireX Deep Scan, ddMS2	99.1, 99.0, 99.1, 98.7	6.767
Coumarin	P. lanceolata leaf	ddMS3, RTLS, AcquireX Deep Scan, ddMS2	90.9, 91.2, 76.3, 84.1	4.961
D-(-)-Quinic acid	Both	ddMS3, RTLS, AcquireX Deep Scan, ddMS2	91.6, 95.2, 78.0, 87.7	2.781
D-Mannitol	Both	ddMS3, RTLS, AcquireX Deep Scan, ddMS2	99.0, 75.4, 86.3, 94.0	0.447
DL-PHENYLALANINE	Both	ddMS3, RTLS, AcquireX Deep Scan, ddMS2	98.3, 98.2, 97.8, 98.3	0.922
DL-Valine	Both	ddMS3, RTLS, AcquireX Deep Scan, ddMS2	98.0, 98.3, 98.8, 95.4	12.425
Dihydroalbocycline	P. lanceolata leaf	ddMS3, RTLS, AcquireX Deep Scan, ddMS2	99.7, 99.5, 99.1, 99.6	9.075
Diosmetin	P. lanceolata leaf	ddMS3, RTLS, AcquireX Deep Scan, ddMS2	99.4, 98.9, 99.8, 99.4	6.743
Esculetin	P. lanceolata leaf	ddMS3, RTLS, AcquireX Deep Scan, ddMS2	96.9, 97.1, 97.7, 97.6	3.002
Estriol	P. lanceolata leaf	ddMS3, RTLS, AcquireX Deep Scan, ddMS2	82.4, 83.7, 84.1, 82.2	8.609
Eurostoside	P. lanceolata leaf	ddMS3, RTLS, AcquireX Deep Scan, ddMS2	94.2, 96.5, 94.8, 94.9	4.672
Everlastoside D	P. lanceolata leaf	ddMS3, RTLS, AcquireX Deep Scan, ddMS2	89.7, 91.2, 86.1, 93.4	3.925
FEGXTMHRTZJSIG-RDRUSSIHSA-N	P. lanceolata leaf	ddMS3, RTLS, AcquireX Deep Scan, ddMS2	90.7, 85.1, 81.1, 85.1	3.441
FYLMQUDVZFGACW-AGBRFASWSA-N	P. lanceolata leaf	ddMS3, RTLS, AcquireX Deep Scan, ddMS2	91.3, 90.1, 91.4, 91.7	10.135
Ferulic Acid Acyl-b-D-glucoside	P. lanceolata leaf	ddMS3, RTLS, AcquireX Deep Scan	91.9, 98.3, 85.3	5.666

Ferulic acid, Z-	Both	ddMS3, RTLS, AcquireX Deep Scan, ddMS2	95.9, 97.2, 97.5, 97.4	5.667
Forsythoside B	P. lanceolata leaf	ddMS3, RTLS, AcquireX Deep Scan	97.2, 97.5, 99.2	4.288
Geniposidic Acid	Both	ddMS3, RTLS, AcquireX Deep Scan, ddMS2	96.9, 96.6, 95.8, 96.9	1.329
Genistein	Both	ddMS3, RTLS, AcquireX Deep Scan, ddMS2	85.9, 86.4, 84.4, 85.0	6.626
Gentisic acid	P. lanceolata leaf	ddMS3, RTLS, AcquireX Deep Scan, ddMS2	87.2, 87.5, 88.9, 87.9	2.484
Glu-Ser-Arg	Both	ddMS3, RTLS, AcquireX Deep Scan, ddMS2	98.1, 97.4, 98.7, 97.2	3.139
Glu-Trp-Pro	P. lanceolata leaf	ddMS3, RTLS, AcquireX Deep Scan, ddMS2	80.1, 75.2, 68.8, 79.3	5.178
Glucosilsteviol	P. lanceolata leaf	ddMS3, RTLS, AcquireX Deep Scan	89.2, 89.1, 89.2	8.366
Grevillol	P. lanceolata leaf	ddMS3, RTLS, AcquireX Deep Scan, ddMS2	83.9, 92.8, 91.0, 89.4	10.375
Guvacoline	P. lanceolata leaf	ddMS3, RTLS, AcquireX Deep Scan, ddMS2	88.8, 89.2, 91.8, 88.7	1.203
HRTKMOMTMZCGLF-QHHAFSJGSA-N	Both	ddMS3, RTLS, AcquireX Deep Scan, ddMS2	76.2, 85.2, 78.0, 77.0	4.532
HZUURLOSYMEMET-QPJJXVBHSA-N	Both	ddMS3, RTLS, AcquireX Deep Scan, ddMS2	99.3, 99.4, 84.0, 99.1	4.297
Hydroxytyrosol	P. lanceolata leaf	ddMS3, RTLS, AcquireX Deep Scan, ddMS2	66.9, 86.6, 63.5, 89.2	1.516
IKRMNTDCFLRTRS-QIDSOVCESA-N	P. lanceolata leaf	ddMS3, RTLS, AcquireX Deep Scan, ddMS2	99.8, 99.6, 99.5, 99.9	6.369
Icariside F2	P. lanceolata leaf	ddMS3, RTLS, AcquireX Deep Scan	94.0, 94.8, 96.1	3.282
Indole-3-acetic acid	P. lanceolata leaf	ddMS3, RTLS, AcquireX Deep Scan, ddMS2	88.7, 93.0, 79.8, 87.5	4.086
Irinotecan	P. lanceolata leaf	ddMS3, RTLS, AcquireX Deep Scan, ddMS2	83.7, 83.4, 82.0, 81.6	12.558
JMVJEEIMQLTXCI-HZJYTTRNSA-N	P. lanceolata leaf	ddMS3, RTLS, AcquireX Deep Scan, ddMS2	96.0, 97.8, 95.1, 75.9	10.142
JRGYPGLKSRGIHK-NQTNIMIGSA-N	P. lanceolata leaf	ddMS3, RTLS, AcquireX Deep Scan, ddMS2	95.6, 95.1, 92.8, 94.7	12.513
Ketologanic acid	Both	ddMS3, RTLS, AcquireX Deep Scan, ddMS2	99.8, 99.8, 99.6, 99.8	1.322
L-isoleucine	P. lanceolata leaf	ddMS3, RTLS, AcquireX Deep Scan, ddMS2	90.0, 93.0, 99.3, 89.1	0.582

LFKQVVDFNHDYNK-XGAYTTIXSA-N	P. lanceolata leaf	ddMS3, RTLS, AcquireX Deep Scan, ddMS2	93.6, 94.8, 90.5, 89.7	4.281
LPLWWIHUJXWQSS-IMNRLACTSA-N	P. lanceolata leaf	ddMS3, RTLS, AcquireX Deep Scan	99.9, 99.9, 99.5	4.946
Lamiuamplexoside C	Both	ddMS3, RTLS, AcquireX Deep Scan, ddMS2	96.6, 96.8, 95.1, 90.1	4.694
Lariciresinol 4-O-glucoside	P. lanceolata leaf	ddMS3, RTLS, AcquireX Deep Scan, ddMS2	93.9, 95.2, 75.8, 69.9	4.157
Licocoumarone	P. lanceolata leaf	ddMS3, RTLS, AcquireX Deep Scan, ddMS2	88.5, 82.7, 62.3, 86.9	4.954
Linoleoyl ethanolamide	Both	ddMS3, RTLS, AcquireX Deep Scan, ddMS2	96.8, 95.7, 92.6, 97.2	11.373
Linolic acid	P. lanceolata leaf	ddMS3, RTLS, AcquireX Deep Scan, ddMS2	98.4, 95.7, 94.5, 98.9	11.017
Loganic acid	Both	ddMS3, RTLS, AcquireX Deep Scan, ddMS2	97.8, 94.5, 98.2, 97.8	2.093
Lonfuranacid A	P. lanceolata leaf	ddMS3, RTLS, AcquireX Deep Scan, ddMS2	93.4, 77.2, 91.6, 76.9	5.244
Lugrandoside	Both	ddMS3, RTLS, AcquireX Deep Scan, ddMS2	96.9, 96.5, 98.0, 97.3	4.279
Luteolin	P. lanceolata leaf	ddMS3, RTLS, AcquireX Deep Scan, ddMS2	94.5, 86.5, 96.1, 95.7	5.943
Luteolin 4'-O-glucoside	Both	ddMS3, RTLS, AcquireX Deep Scan, ddMS2	97.4, 99.3, 84.6, 88.7	4.462
MECYDNMVWUSMSU-YTLDOUCOSA-N	P. lanceolata leaf	ddMS3, RTLS, AcquireX Deep Scan, ddMS2	70.7, 83.4, 82.7, 83.2	8.421
Maleic acid	P. lanceolata leaf	ddMS3, RTLS, AcquireX Deep Scan, ddMS2	98.3, 88.7, 99.6, 81.5	0.513
Malonic acid	Both	ddMS3, RTLS, AcquireX Deep Scan, ddMS2	98.2, 98.1, 96.1, 97.8	0.541
Manidipine	P. lanceolata leaf	ddMS3, RTLS, AcquireX Deep Scan, ddMS2	61.8, 60.3, 63.3, 80.2	12.79
Matairesinoside	P. lanceolata leaf	ddMS3, RTLS, AcquireX Deep Scan, ddMS2	80.4, 80.2, 78.4, 79.7	4.391
Methyl 2-{[2-O-(6-deoxy-α-L-mannopyranosyl)-β-D-glucopyranosyl]oxy}benzoate	P. lanceolata leaf	ddMS3, RTLS, AcquireX Deep Scan, ddMS2	99.9, 99.9, 99.7, 99.9	3.454
Mussaenosidic acid	P. lanceolata leaf	ddMS3, RTLS, AcquireX Deep Scan, ddMS2	94.0, 97.6, 92.8, 68.5	1.148
Myrciaphenone A	Both	ddMS3, RTLS, AcquireX Deep Scan, ddMS2	70.0, 85.8, 69.5, 92.2	1.258

N,N-Dimethyl-4-{5-[(3S)-1-(phenylsulfonyl)-3-pyrrolidinyl]-1,3,4-oxadiazol-2-yl}aniline	P. lanceolata leaf	ddMS3, RTLS, AcquireX Deep Scan, ddMS2	97.5, 96.9, 98.0, 97.7	3.151
N-({(1S,4S,6S)-6-Isopropyl-3-methyl-4-[2-oxo-2-(1-pyrrolidinyl)ethyl]-2-cyclohexen-1-yl}methyl)-2-pyrazinecarboxamide	P. lanceolata leaf	ddMS3, RTLS, AcquireX Deep Scan, ddMS2	99.5, 99.3, 99.9, 99.1	8.388
N-Ethyl-2-[(14E,16S,17S)-8-(4-morpholinyl)-2-oxo-12-oxa-1,4-diazatricyclo[14.3.1.06,11]icosa-6,8,10,14-tetraen-17-yl]acetamide	P. lanceolata leaf	ddMS3, RTLS, AcquireX Deep Scan, ddMS2	99.8, 99.7, 99.7, 99.6	8.506
N-Isovalerylglycine	P. lanceolata leaf	ddMS3, RTLS, AcquireX Deep Scan, ddMS2	90.8, 98.8, 93.9, 96.9	3.178
N-{(1S,2S,4aS,7S,8S,8aS)-8-Hydroxy-1,4a-dimethyl-7-[(2S)-1-oxo-1-(1-piperidinyl)-2-propanyl]decahydro-2-naphthalenyl}-5-pyrimidinecarboxamide	P. lanceolata leaf	ddMS3, RTLS, AcquireX Deep Scan, ddMS2	99.4, 99.4, 99.2, 99.2	10.248
N1-(4-{3-[5-(trifluoromethyl)-2-pyridyl]-4,5-dihydro-1H-1,2,4-triazol-5-yl}phenyl)acetamide	P. lanceolata leaf	ddMS3, RTLS, AcquireX Deep Scan, ddMS2	95.6, 94.1, 96.5, 91.0	0.914
N1-cyclohexyl-2-[{2-[(4-chlorophenyl)thio]acetyl}(methyl)amino]benzamide	P. lanceolata leaf	ddMS3, RTLS, AcquireX Deep Scan, ddMS2	99.4, 99.4, 98.6, 98.5	4.099
Neochlorogenic acid	P. lanceolata leaf	ddMS3, RTLS, AcquireX Deep Scan, ddMS2	92.9, 93.1, 92.0, 93.0	1.787
Nicotinamide	P. lanceolata leaf	ddMS3, RTLS, AcquireX Deep Scan, ddMS2	96.0, 84.9, 90.9, 94.9	0.528
Norharman	P. lanceolata leaf	ddMS3, RTLS, AcquireX Deep Scan, ddMS2	96.1, 85.7, 97.6, 96.7	3.111
OGAIGVUECHDBJB-SKPUKWKESA-N	P. lanceolata leaf	ddMS3, RTLS, AcquireX Deep Scan, ddMS2	96.7, 97.4, 97.5, 97.2	4.234
OQWOKDQAPBSVGH-UHFFFAOYSA-N	P. lanceolata leaf	ddMS3, RTLS, AcquireX Deep Scan, ddMS2	94.1, 62.6, 91.4, 93.2	9.818
OZHUIFOZCHBIOL-KUDUMFAVSA-N	P. lanceolata leaf	ddMS3, RTLS, AcquireX Deep Scan, ddMS2	90.0, 91.7, 95.3, 89.9	5.545
Octadecenedioic acid	P. lanceolata leaf	ddMS3, RTLS, AcquireX Deep Scan	97.5, 89.4, 95.6	8.921
Oleamide	Both	ddMS3, RTLS, AcquireX Deep Scan, ddMS2	92.4, 93.8, 97.0, 93.9	12.558
Oleanolic acid	P. lanceolata leaf	ddMS3, RTLS, AcquireX Deep Scan, ddMS2	98.8, 95.3, 76.9, 97.4	12.417
Oleic acid alkyne	P. lanceolata leaf	ddMS3, RTLS, AcquireX Deep Scan, ddMS2	99.9, 100.0, 100.0, 100.0	10.25
Oleoyl ethanolamide	P. lanceolata leaf	ddMS3, RTLS, AcquireX Deep Scan, ddMS2	95.4, 97.8, 95.4, 92.5	11.967
PEG Monolaurate n5	P. lanceolata leaf	ddMS3, RTLS, AcquireX Deep Scan, ddMS2	100.0, 99.7, 99.9, 99.9	10.869

Palmitoyl ethanolamide	P. lanceolata leaf	ddMS3, RTLS, AcquireX Deep Scan	97.6, 99.4, 97.1	11.522
Pentoxifylline	P. lanceolata leaf	ddMS3, RTLS, AcquireX Deep Scan, ddMS2	98.4, 99.1, 98.9, 97.9	1.188
Phe-Ile-Gln	P. lanceolata leaf	ddMS3, RTLS, AcquireX Deep Scan, ddMS2	99.7, 99.4, 99.7, 99.7	2.795
Phenethyl sophoroside	Both	ddMS3, RTLS, AcquireX Deep Scan, ddMS2	85.8, 92.0, 69.0, 84.0	3.426
Phenylethyl primeveroside	P. lanceolata leaf	ddMS3, RTLS, AcquireX Deep Scan, ddMS2	97.3, 98.9, 98.9, 94.3	3.019
Phlinoside A	Both	ddMS3, RTLS, AcquireX Deep Scan	81.2, 81.4, 81.4	4.389
Phlomisionoside	Both	ddMS3, RTLS, AcquireX Deep Scan, ddMS2	99.7, 89.6, 97.7, 99.7	3.392
Piliformic acid	P. lanceolata leaf	ddMS3, RTLS, AcquireX Deep Scan, ddMS2	96.8, 91.1, 96.1, 87.9	6.436
Plantainoside C	P. lanceolata leaf	ddMS3, RTLS, AcquireX Deep Scan, ddMS2	99.9, 86.8, 90.6, 99.0	5.046
Protocatehuic acid	P. lanceolata leaf	ddMS3, RTLS, AcquireX Deep Scan, ddMS2	91.4, 83.0, 89.7, 76.3	1.48
QRUIPHBZVJDDTR-KTQHHXNFSA-N	P. lanceolata leaf	ddMS3, RTLS, AcquireX Deep Scan, ddMS2	99.1, 97.2, 87.2, 97.5	11.33
RBTWPFBRVJRSRZ-VLBJUCMXSA-N	P. lanceolata leaf	ddMS3, RTLS, AcquireX Deep Scan	98.0, 96.6, 99.8	4.483
RLGRBYHBNWLGER-RMKNXTFCSA-N	P. lanceolata leaf	ddMS3, RTLS, AcquireX Deep Scan, ddMS2	99.9, 99.8, 99.9, 99.6	5.667
Rhamnetin 3-galactoside	Both	ddMS3, RTLS, AcquireX Deep Scan	78.4, 77.3, 95.0	4.815
SAHCQBPGXQFTRA-MTWZWZNHSA-N	Both	ddMS3, RTLS, AcquireX Deep Scan, ddMS2	96.6, 60.6, 87.5, 88.3	10.234
SUFSOKMJLLAQQX-JYBASQMISA-N	P. lanceolata leaf	ddMS3, RTLS, AcquireX Deep Scan, ddMS2	79.0, 77.6, 93.4, 90.6	6.92
SXLKGCCRNBGMMM-VDFSFMFYSA-N	P. lanceolata leaf	ddMS3, RTLS, AcquireX Deep Scan, ddMS2	99.0, 99.3, 98.1, 99.0	2.921
Salicylic acid	P. lanceolata leaf	ddMS3, RTLS, AcquireX Deep Scan, ddMS2	97.8, 97.9, 83.7, 93.2	4.792
Scutellarin	P. lanceolata leaf	ddMS3, RTLS, AcquireX Deep Scan, ddMS2	91.2, 90.5, 91.5, 91.3	4.518
Sebacic acid	P. lanceolata leaf	ddMS3, RTLS, AcquireX Deep Scan, ddMS2	99.4, 90.5, 98.5, 99.3	5.875
Secologanin	P. lanceolata leaf	ddMS3, RTLS, AcquireX Deep Scan, ddMS2	98.9, 99.4, 91.0, 98.2	2.502

Secosterigmatocystin	Both	ddMS3, RTLS, AcquireX Deep Scan, ddMS2	98.7, 99.0, 74.1, 97.3	0.767
Sparfloxacin	P. lanceolata leaf	ddMS3, RTLS, AcquireX Deep Scan	94.5, 99.5, 85.5	4.029
Spiroxamine	Both	ddMS3, RTLS, AcquireX Deep Scan	97.3, 97.4, 96.8	9.923
Squamocin L	P. lanceolata leaf	ddMS3, RTLS, AcquireX Deep Scan, ddMS2	72.7, 97.6, 72.2, 72.7	13.164
Succinic acid	Both	ddMS3, RTLS, AcquireX Deep Scan, ddMS2	99.7, 99.7, 99.6, 99.7	0.685
Sucrose	P. lanceolata leaf	ddMS3, RTLS, AcquireX Deep Scan, ddMS2	99.6, 99.7, 99.3, 99.5	0.46
Teucardoside	P. lanceolata leaf	ddMS3, RTLS, AcquireX Deep Scan, ddMS2	99.8, 99.9, 99.7, 99.8	3.332
Trans-aconitic acid	Both	ddMS3, RTLS, AcquireX Deep Scan, ddMS2	99.5, 99.0, 99.4, 99.4	0.588
Traumatic acid	P. lanceolata leaf	ddMS3, RTLS, AcquireX Deep Scan, ddMS2	98.3, 96.2, 91.9, 93.5	7.199
Trp-Arg-Arg	P. lanceolata leaf	ddMS3, RTLS, AcquireX Deep Scan, ddMS2	99.7, 94.9, 87.6, 99.6	7.355
Tryptophan	P. lanceolata leaf	ddMS3, RTLS, AcquireX Deep Scan, ddMS2	97.3, 97.7, 97.9, 97.3	1.722
UDHCHDJLZGYDDM-QPJJXVBHSA-N	Both	ddMS3, RTLS, AcquireX Deep Scan, ddMS2	99.0, 98.9, 99.2, 99.3	4.443
UZPUBNINOVQPMB-AZGBBHLQSA-N	P. lanceolata leaf	ddMS3, RTLS, AcquireX Deep Scan, ddMS2	83.2, 84.6, 83.9, 84.1	5.418
Undecanedionic acid	P. lanceolata leaf	ddMS3, RTLS, AcquireX Deep Scan, ddMS2	99.8, 99.7, 97.6, 98.8	6.691
VOGZHCQKYXJSRT-UHFFFAOYSA-N	P. lanceolata leaf	ddMS3, RTLS, AcquireX Deep Scan, ddMS2	98.1, 93.3, 98.8, 98.1	9.688
VOYZLKWKVLYJHD-PSNLIWPNSA-N	P. lanceolata leaf	ddMS3, RTLS, AcquireX Deep Scan, ddMS2	93.9, 94.8, 94.9, 93.8	11.963
VQWGPODDCMJNCY-VOTSOKGWSA-N	Both	ddMS3, RTLS, AcquireX Deep Scan, ddMS2	94.7, 95.5, 69.5, 98.5	3.383
VSZGUTBXIPJUAN-UHFFFAOYSA-N	P. lanceolata leaf	ddMS3, RTLS, AcquireX Deep Scan, ddMS2	90.4, 92.7, 93.7, 83.8	6.901
Vanillic acid	P. lanceolata leaf	ddMS3, RTLS, AcquireX Deep Scan, ddMS2	91.5, 96.2, 65.5, 74.9	1.241
WCGUUGGRBIKTOS-POPPTPIPSA-N	P. lanceolata leaf	ddMS3, RTLS, AcquireX Deep Scan, ddMS2	95.5, 93.1, 94.3, 94.4	12.219
XBWNIQFQKJRWAM-ARNLHZKISA-N	P. lanceolata leaf	ddMS3, RTLS, AcquireX Deep Scan, ddMS2	87.1, 88.4, 82.1, 85.8	10.403

XWRHBGVVCOSNKO-OTCYKTEZSA-N	P. lanceolata leaf	ddMS3, RTLS, AcquireX Deep Scan, ddMS2	98.6, 98.5, 88.3, 98.5	3.546
ZUXLIBRTWRBKLQ-UTFMUXIPSA-N	P. lanceolata leaf	ddMS3, RTLS, AcquireX Deep Scan, ddMS2	99.9, 99.7, 99.9, 99.9	3.751
[(2R,3S,4S,5S)-3,4-dihydroxy-5-(hydroxymethyl)-5-[(2R,3R,4S,5S,6R)-3,4,5-trihydroxy-6-(hydroxymethyl)oxan-2-yl]oxyoxolan-2-yl]methyl (E)-3-phenylprop-2-enoate	P. lanceolata leaf	ddMS3, RTLS, AcquireX Deep Scan, ddMS2	99.2, 99.0, 98.4, 99.4	4.084
[3-[2,3-Dihydroxypropoxy(hydroxy)phosphoryl]oxy-2-hydroxypropyl] 14-methylpentadecanoate	Both	ddMS3, RTLS, AcquireX Deep Scan, ddMS2	97.5, 96.9, 96.3, 97.9	11.121
ethyl 3-amino-1-[6-(4-methylpiperidino)pyridazin-3-yl]-1H-pyrazole-4-carboxylate	P. lanceolata leaf	ddMS3, RTLS, AcquireX Deep Scan, ddMS2	99.0, 99.2, 98.8, 99.0	8.205
p-Tolualdehyde	P. lanceolata leaf	ddMS3, RTLS, AcquireX Deep Scan, ddMS2	85.9, 88.9, 87.5, 88.8	0.765
{(1R,2R)-2-[(2Z)-5-(Hexopyranosyloxy)-2-penten-1-yl]-3-oxocyclopentyl}acetic acid	P. lanceolata leaf	ddMS3, RTLS, AcquireX Deep Scan, ddMS2	91.5, 91.9, 88.8, 90.9	3.208
α-Linolenic acid	Both	ddMS3, RTLS, AcquireX Deep Scan, ddMS2	98.0, 99.2, 98.2, 98.3	12.417
α-Linolenoyl ethanolamide	P. lanceolata leaf	ddMS3, RTLS, AcquireX Deep Scan, ddMS2	79.1, 82.2, 62.6, 81.5	10.842
(+/-)13-HODE	Both	RTLS, AcquireX Deep Scan, ddMS2	75.5, 96.8, 92.2	8.815
(+/-)9(10)-EpOME	P. lanceolata leaf	RTLS, AcquireX Deep Scan, ddMS2	96.4, 87.5, 79.9	8.925
(+/-)9-HpODE	Both	ddMS3, AcquireX Deep Scan, ddMS2	69.9, 95.6, 82.9	8.962
(-)-Myrtenal	P. lanceolata leaf	ddMS3, AcquireX Deep Scan	93.3, 92.1	4.688
(14E,16S,17S)-17-{2-[(2R)-2-(Methoxymethyl)-1-pyrrolidinyl]-2-oxoethyl}-8-(1-piperazinyl)-12-oxa-1,4-diazatricyclo[14.3.1.06,11]icosa-6,8,10,14-tetraen-2-one	P. lanceolata leaf	RTLS, AcquireX Deep Scan, ddMS2	82.9, 86.4, 83.0	9.114
(1R,8S,9S)-3,4,8-trihydroxy-11,11-dimethyl-5-propan-2-yl-16-oxatetracyclo[7.5.2.01,10.02,7]hexadeca-2,4,6-trien-15-one	P. lanceolata leaf	ddMS3, RTLS, ddMS2	94.2, 95.6, 95.2	7.888
(2R,3R,4S,5S,6R)-2-[(3E)-1-hydroxy-3,7-dimethylocta-3,6-dien-2-yl]oxy-6-(hydroxymethyl)oxane-3,4,5-triol	P. lanceolata leaf	ddMS3, AcquireX Deep Scan	83.8, 75.1	3.87
(2R,3R,4S,5S,6R)-2-[[(1S,5R,7R,8S,8aS)-5,7-dihydroxy-8-(hydroxymethyl)-4,4,7,8a-tetramethyl-2,3,4a,5,6,8-hexahydro-1H-naphthalen-1-yl]oxy]-6-(hydroxymethyl)oxane-3,4,5-triol	P. lanceolata leaf	ddMS3, AcquireX Deep Scan, ddMS2	90.0, 96.5, 87.5	4.408

(2R,3S,4S,5R,6S)-2-(hydroxymethyl)-6-(3-methoxy-4-prop-2-enylphenoxy)oxane-3,4,5-triol	P. lanceolata leaf	RTLS, AcquireX Deep Scan, ddMS2	89.7, 88.7, 91.8	5.154
(2S)-2-Hydroxy-3-[(hydroxy{[(1S,2R,3R,4S,5S,6R)-2,3,4,5,6-pentahydroxycyclohexyl]oxy}phosphoryl)oxy]propyl (12Z)-12-octadecenoate	Both	ddMS3, AcquireX Deep Scan, ddMS2	87.6, 87.5, 87.2	10.347
(3R,5R)-3,4,5-trihydroxy-1-[(E)-3-(4-hydroxy-3-methoxyphenyl)prop-2-enoyl]oxycyclohexane-1-carboxylic acid	P. lanceolata leaf	RTLS, AcquireX Deep Scan, ddMS2	93.6, 97.0, 95.5	3.857
(3S)-3-{[(3-Methoxyphenyl)carbamoyl]amino}-1-[(methylsulfanyl)acetyl]-L-prolyl-N5-[(methylsulfanyl)acetyl]-L-ornithinamide	P. lanceolata leaf	RTLS, AcquireX Deep Scan, ddMS2	90.4, 94.4, 90.9	4.361
(5Z)-3-(?-D-Glucopyranosyloxy)-5-octenoic acid	P. lanceolata leaf	ddMS3, AcquireX Deep Scan, ddMS2	97.7, 97.2, 98.4	3.265
(9Z)-4,12,12-trimethyl-5-oxatricyclo[9.1.0.04,6]dodec-9-ene-9-carboxylic acid	P. lanceolata leaf	RTLS, AcquireX Deep Scan, ddMS2	73.7, 79.6, 81.5	5.382
(Z)-2-oct-7-enylpent-2-enedioic acid	P. lanceolata leaf	RTLS, AcquireX Deep Scan	76.5, 82.5	7.684
(e)-4-Hydroxy-dodec-2-enedioic acid	Both	ddMS3, AcquireX Deep Scan, ddMS2	95.9, 98.4, 97.7	5.538
1,4:3,6-Dianhydro-2-[(cyclohexylcarbonyl)amino]-5-[(4-cyclopentyl-2-pyrimidinyl)amino]-2,5-dideoxy-L-iditol	P. lanceolata leaf	RTLS, AcquireX Deep Scan, ddMS2	95.5, 80.1, 79.6	6.373
1-Linoleoyl glycerol	P. lanceolata leaf	RTLS, AcquireX Deep Scan, ddMS2	68.5, 92.0, 93.3	11.287
1-O-[(3β,5ξ,9ξ,18ξ)-3,19,24-Trihydroxy-24,28-dioxours-12-en-28-yl]hexopyranose	P. lanceolata leaf	RTLS, AcquireX Deep Scan, ddMS2	90.1, 79.6, 89.4	6.91
1-{(2R,4S,5R)-5-[3-(3,4-Dimethoxyphenyl)-1-methyl-1H-pyrazol-5-yl]-1-azabicyclo[2.2.2]oct-2-yl}-N-(4-methoxybenzyl)methanamine	P. lanceolata leaf	RTLS, AcquireX Deep Scan	97.1, 71.0	8.82
1-{4-[(3S)-3-(5-Methyl-1H-benzimidazol-2-yl)-1-pyrrolidinyl]-1-piperidinyl}ethanone	P. lanceolata leaf	RTLS, AcquireX Deep Scan	98.5, 98.3	6.87
11(13)-Dehydroivaxillin	P. lanceolata leaf	ddMS3, AcquireX Deep Scan, ddMS2	85.2, 82.9, 87.4	5.832
11(Z),14(Z),17(Z)-Eicosatrienoic acid	P. lanceolata leaf	RTLS, AcquireX Deep Scan, ddMS2	92.9, 79.4, 93.6	11.146
11(Z),14(Z)-Eicosadienoic acid	P. lanceolata leaf	RTLS, AcquireX Deep Scan, ddMS2	97.6, 93.5, 95.1	11.61
16-Hydroxyhexadecanoic acid	P. lanceolata leaf	ddMS3, RTLS, ddMS2	99.0, 96.3, 93.9	11.881
18-β-Glycyrrhetinic acid	P. lanceolata leaf	ddMS3, RTLS, ddMS2	97.9, 98.2, 98.5	10.521
2,4-Dihydroxybenzoic acid	P. lanceolata leaf	RTLS, AcquireX Deep Scan, ddMS2	88.5, 73.2, 82.3	3.613

2,4-Dimethylphenol	P. lanceolata leaf	ddMS3, AcquireX Deep Scan	96.9, 97.9	1.214
2,5-Dihydroxybenzaldehyde	Both	RTLS, AcquireX Deep Scan, ddMS2	94.4, 95.2, 95.5	2.469
2-(4-Hydroxyphenyl)ethyl 6-O-[(2R,3R,4R)-3,4-dihydroxy-4-(hydroxymethyl)tetrahydro-2-furanyl]-beta-D-glucopyranoside	P. lanceolata leaf	ddMS3, RTLS, ddMS2	90.7, 87.6, 87.2	2.759
2-(tert-Butyl)-6-[(4-chlorophenyl)sulfonyl]pyrazolo[1,5-a]pyrimidin-7-amine	P. lanceolata leaf	RTLS, AcquireX Deep Scan, ddMS2	95.3, 65.8, 81.9	2.247
2-Hexenyl-beta-glucopyranoside	P. lanceolata leaf	ddMS3, RTLS, ddMS2	99.6, 98.4, 99.1	4.719
2-Hydroxy-3-[2-hydroxy-4-methoxy-5-(2-methylbut-3-en-2-yl)phenyl]-1-(4-hydroxyphenyl)-3-methoxypropan-1-one	P. lanceolata leaf	RTLS, AcquireX Deep Scan	99.8, 67.7	4.902
2-[(1S,4S,5S)-4-(Hydroxymethyl)-5-isopropyl-2-methyl-2-cyclohexen-1-yl]-1-(4-methyl-1-piperidinyl)ethanone	P. lanceolata leaf	RTLS, AcquireX Deep Scan, ddMS2	82.9, 77.2, 81.0	4.942
2-[3,8-Dihydroxy-8-(hydroxymethyl)-3-methyl-2-oxodecahydro-5-azulenyl]-2-propanyl hexopyranoside	P. lanceolata leaf	ddMS3, RTLS	86.2, 60.9	6.013
3,5-di-tert-Butyl-4-hydroxybenzyl alcohol	P. lanceolata leaf	RTLS, AcquireX Deep Scan, ddMS2	95.8, 65.8, 96.2	10.109
3-Hydroxy-3-methylglutaric acid	Both	ddMS3, AcquireX Deep Scan	90.7, 87.6	0.737
3-Methoxysalicylic acid	P. lanceolata leaf	RTLS, AcquireX Deep Scan, ddMS2	94.6, 73.6, 92.4	1.401
3-Methyl-5-(5,5,8a-trimethyl-2-methylene-7-oxodecahydro-1-naphthalenyl)pentyl acetate	P. lanceolata leaf	RTLS, AcquireX Deep Scan, ddMS2	82.3, 67.5, 78.8	15.369
3-[(1E,3E)-hepta-1,3-dienyl]pentanedioic acid	P. lanceolata leaf	RTLS, AcquireX Deep Scan	91.5, 89.2	3.208
3-[(6-O-alpha-L-Arabinopyranosyl-beta-D-glucopyranosyl)oxy]-1-octene	Both	ddMS3, RTLS, ddMS2	96.2, 94.3, 94.8	5.395
3-{4-[(1,3-Dihydroxy-2-propanyl)oxy]-3-methoxyphenyl}propyl ?-D-glucopyranoside	P. lanceolata leaf	ddMS3, AcquireX Deep Scan, ddMS2	90.7, 78.2, 64.7	4.923
4-Anisic acid	P. lanceolata leaf	RTLS, AcquireX Deep Scan, ddMS2	88.6, 65.1, 77.3	2.089
4-Hydroxy-4-(3-hydroxy-1-butenyl)-3,5,5-trimethyl-2-cyclohexen-1-one	P. lanceolata leaf	ddMS3, RTLS, ddMS2	93.3, 91.9, 91.7	3.406
4-Phenylbutyric acid	P. lanceolata leaf	ddMS3, AcquireX Deep Scan	90.0, 84.0	15.603
5,7-Dihydroxy-2-(4-hydroxyphenyl)-6,8-bis[3,4,5-trihydroxy-6-(hydroxymethyl)tetrahydro-2H-pyran-2-yl]-4H-chromen-4-one	Both	RTLS, AcquireX Deep Scan, ddMS2	85.4, 75.6, 68.3	3.491
5-(Carboxymethyl)-5,6,8a-trimethyl-3,4,4a,6,7,8-hexahydronaphthalene-1-carboxylic acid	P. lanceolata leaf	RTLS, AcquireX Deep Scan, ddMS2	84.3, 81.2, 77.5	7.785
5-Hydroxy-2',3',4',5',7-pentamethoxyflavone	P. lanceolata leaf	RTLS, AcquireX Deep Scan, ddMS2	87.2, 85.9, 86.2	9.012

5-[(3Z)-5-Hydroxy-3-methyl-3-penten-1-yl]-1,4a-dimethyl-6-methylenedecahydro-1-naphthalenecarboxylic acid	P. lanceolata leaf	RTLS, AcquireX Deep Scan, ddMS2	98.9, 89.3, 92.0	10.381
7,9-Dihydroxy-3,6-dimethyl-10-methylidene-3,3a,4,5,6,7,8,9,11,11a-decahydrocyclodeca[b]furan-2-one	P. lanceolata leaf	RTLS, AcquireX Deep Scan, ddMS2	80.7, 80.2, 82.8	6.714
7-Hydroxy-3-[4-hydroxy-3-(3-methyl-2-buten-1-yl)phenyl]-8-(3-methyl-2-buten-1-yl)-4H-chromen-4-one	P. lanceolata leaf	ddMS3, AcquireX Deep Scan, ddMS2	98.6, 67.7, 99.0	12.49
8(S)-Hydroxy-(5Z,9E,11Z,14Z)-eicosatetraenoic acid	P. lanceolata leaf	ddMS3, RTLS, ddMS2	97.5, 94.6, 94.7	10.374
8-Oxo-9-(3,4,5-trimethoxyphenyl)-5,5a,6,8,8a,9-hexahydrofuro[3',4':6,7]naphtho[2,3-d][1,3]dioxol-5-yl hexopyranoside	P. lanceolata leaf	RTLS, AcquireX Deep Scan	60.6, 80.9	5.103
9-Octadecynoic acid	P. lanceolata leaf	RTLS, AcquireX Deep Scan	84.3, 84.8	8.789
Abscisic acid	P. lanceolata leaf	ddMS3, AcquireX Deep Scan, ddMS2	92.9, 75.4, 95.8	5.88
Acetylleucine	P. lanceolata leaf	RTLS, AcquireX Deep Scan, ddMS2	95.0, 88.9, 86.5	3.343
Actinopyrone A	P. lanceolata leaf	RTLS, AcquireX Deep Scan, ddMS2	83.6, 70.9, 79.6	11.156
Ala-Phe-Gln	P. lanceolata leaf	RTLS, AcquireX Deep Scan, ddMS2	96.1, 88.2, 97.4	3.039
Arachidonic acid	P. lanceolata leaf	RTLS, AcquireX Deep Scan	82.5, 63.0	9.792
Arecoline	P. lanceolata leaf	RTLS, AcquireX Deep Scan	83.8, 86.1	1.47
Asn-Asp-Tyr	P. lanceolata leaf	RTLS, AcquireX Deep Scan	80.2, 76.1	1.062
Asp-Trp-Pro	P. lanceolata leaf	RTLS, AcquireX Deep Scan, ddMS2	84.7, 73.9, 85.2	3.968
Asperuloside	P. lanceolata leaf	RTLS, AcquireX Deep Scan, ddMS2	93.0, nan, nan	3.299
Asperulosidic acid	P. lanceolata leaf	RTLS, AcquireX Deep Scan, ddMS2	89.7, 88.7, 91.1	2.54
Atropine	P. lanceolata leaf	RTLS, AcquireX Deep Scan, ddMS2	87.0, 96.3, 96.5	3.508
BVXGSXUEENMHSG-NAMAEXMOSA-N	P. lanceolata leaf	ddMS3, AcquireX Deep Scan, ddMS2	98.6, 97.4, 98.6	11.097
Beta-Sitosterol	Both	ddMS3, AcquireX Deep Scan, ddMS2	96.3, 94.2, 94.7	13.216
Bisisomahanine	P. lanceolata leaf	ddMS3, AcquireX Deep Scan	88.3, 89.3	7.712
CQYWFDMYHSTSJB-ODPDPSAISA-N	Both	ddMS3, RTLS, ddMS2	89.0, 87.6, 90.5	3.124
CTHCZGUSHSEEMA-UHFFFAOYSA-N	P. lanceolata leaf	RTLS, AcquireX Deep Scan	68.6, 92.4	2.455
Cabergoline	P. lanceolata leaf	RTLS, AcquireX Deep Scan, ddMS2	66.9, 88.4, 86.3	4.384
Carboprost	P. lanceolata leaf	RTLS, AcquireX Deep Scan, ddMS2	98.6, 98.8, 98.1	9.452
Choline	Both	RTLS, AcquireX Deep Scan	99.3, 99.6	0.444

Citric Acid	Both	ddMS3, RTLS	97.7, 99.2	0.559
Crotonic acid	P. lanceolata leaf	RTLS, AcquireX Deep Scan	94.2, 94.1	0.46
D-Pantothenic acid	Both	RTLS, AcquireX Deep Scan, ddMS2	87.0, 90.8, 92.0	1.141
D-Tagatose	P. lanceolata leaf	ddMS3, AcquireX Deep Scan	96.6, 82.1	0.455
D-α-Tocopherol	P. lanceolata leaf	ddMS3, AcquireX Deep Scan	87.2, 80.3	15.6
DL-Tryptophan	P. lanceolata leaf	RTLS, AcquireX Deep Scan, ddMS2	88.0, 63.7, 64.1	1.099
DOXUPTYBRZGKMG-OLALOJMBSA-N	P. lanceolata leaf	RTLS, AcquireX Deep Scan, ddMS2	61.3, 92.3, 82.1	1.883
DVJBCXWGDIOZRH-DPABJOKRSA-N	P. lanceolata leaf	RTLS, AcquireX Deep Scan	93.3, 69.8	1.986
Dalpiciclib	P. lanceolata leaf	ddMS3, AcquireX Deep Scan, ddMS2	87.1, 88.9, 88.3	10.896
Deferiprone	P. lanceolata leaf	RTLS, AcquireX Deep Scan, ddMS2	77.8, 76.4, 86.0	2.473
Delamanid	P. lanceolata leaf	RTLS, AcquireX Deep Scan	93.3, 62.8	3.731
Dimethylcaffeic acid	P. lanceolata leaf	ddMS3, AcquireX Deep Scan	88.8, 74.6	3.999
Dracocephaloside	P. lanceolata leaf	RTLS, AcquireX Deep Scan, ddMS2	96.8, 84.8, 94.8	4.958
Echinocystic aicd	P. lanceolata leaf	RTLS, AcquireX Deep Scan, ddMS2	98.2, 90.7, 96.7	9.993
Epidihydrophaseic acid	P. lanceolata leaf	ddMS3, RTLS, ddMS2	90.4, 94.7, 71.4	2.602
Esculin	P. lanceolata leaf	RTLS, AcquireX Deep Scan, ddMS2	97.5, 77.3, 87.7	2.444
FBZGBPCYCJJRJK-JGIGNMFISA-N	P. lanceolata leaf	RTLS, AcquireX Deep Scan	86.0, 83.0	6.795
FNTJVYCFNVUBOL-YUWSMODRSA-N	P. lanceolata leaf	RTLS, AcquireX Deep Scan	91.2, 93.3	4.554
Fumaric acid	Both	ddMS3, RTLS, ddMS2	99.7, 99.5, 99.5	0.612
Furfural	Both	ddMS3, AcquireX Deep Scan, ddMS2	94.8, 89.9, 93.1	4.91
GLUTARIC ACID	P. lanceolata leaf	ddMS3, AcquireX Deep Scan, ddMS2	99.1, 85.4, 98.4	0.53
Genistein 4'-O-glucuronide	P. lanceolata leaf	ddMS3, RTLS	96.7, 96.3	5.024
Gibepyrone D	P. lanceolata leaf	RTLS, AcquireX Deep Scan	80.7, 82.9	1.324
Gluconic acid	P. lanceolata leaf	ddMS3, RTLS	97.3, 98.1	0.464
Glychionide A	P. lanceolata leaf	ddMS3, RTLS	87.9, 92.7	5.018
Hypoxanthine	P. lanceolata leaf	ddMS3, AcquireX Deep Scan, ddMS2	81.6, 86.3, 87.4	0.547
IEDTVFQRYOEXGI-RQUWEGHFSA-N	P. lanceolata leaf	ddMS3, RTLS, ddMS2	99.7, 99.6, 99.6	11.006
ISQNOTIPEBTHRS-KJGZCIDPSA-N	P. lanceolata leaf	RTLS, AcquireX Deep Scan, ddMS2	91.3, 88.7, 91.5	11.149
ISXANBIHUVHPID-GVQNEMQFSA-N	Both	ddMS3, RTLS, ddMS2	83.4, 97.8, 91.1	3.691

Isorhamnetin	P. lanceolata leaf	RTLS, AcquireX Deep Scan, ddMS2	99.2, 92.7, 82.8	4.817
Isovanillic acid	P. lanceolata leaf	RTLS, AcquireX Deep Scan, ddMS2	90.4, 94.8, 75.6	1.334
KHTIKCLSZWGZRQ-UHFFFAOYSA-N	P. lanceolata leaf	RTLS, AcquireX Deep Scan, ddMS2	86.3, 87.3, 86.0	8.285
L-Norleucine	Both	ddMS3, RTLS, ddMS2	99.1, 98.6, 98.6	0.578
L-phenylalanine	Both	ddMS3, RTLS, ddMS2	98.3, 97.7, 97.1	0.916
L-valine	Both	ddMS3, RTLS, ddMS2	96.3, 98.4, 92.5	0.498
LCIUOVOXWPIXOR-AMAMRJOVSA-N	P. lanceolata leaf	RTLS, AcquireX Deep Scan, ddMS2	99.9, 99.7, 90.8	10.049
LFAZJMJJLQUOPK-UHFFFAOYSA-N	P. lanceolata leaf	RTLS, AcquireX Deep Scan, ddMS2	88.4, 94.2, 94.9	5.804
LOPCJHUBECHTEA-BJGSYIFTSA-N	P. lanceolata leaf	RTLS, AcquireX Deep Scan, ddMS2	86.5, 81.8, 83.6	3.303
Malic acid	P. lanceolata leaf	ddMS3, RTLS	98.4, 98.1	0.512
Maltol	P. lanceolata leaf	ddMS3, RTLS, ddMS2	98.6, 97.5, 98.4	1.618
Matairesinol	P. lanceolata leaf	ddMS3, RTLS, ddMS2	82.7, 64.1, 81.7	5.461
Menthofuran	P. lanceolata leaf	RTLS, AcquireX Deep Scan, ddMS2	84.8, 85.3, 84.4	4.679
Mesaconic acid	P. lanceolata leaf	RTLS, AcquireX Deep Scan	94.4, 94.4	0.668
Methyl 1-{3-[(2S,5aS,8aR)-6-(4-fluorobenzyl)-1-methyl-5-oxodecahydropyrrolo[3,2-E][1,4]diazepin-2-yl]propanoyl}-4-piperidinecarboxylate	P. lanceolata leaf	RTLS, AcquireX Deep Scan, ddMS2	99.7, 98.7, 99.1	8.384
Metoprolol	P. lanceolata leaf	ddMS3, RTLS	89.0, 90.2	2.763
Monopalmitin	Both	ddMS3, AcquireX Deep Scan, ddMS2	85.3, 93.1, 93.2	10.037
N-(4-{[(1R,9S)-11-(4-Fluorobenzyl)-6-oxo-7,11-diazatricyclo[7.3.1.02,7]trideca-2,4-dien-5-yl]sulfamoyl}phenyl)acetamide	P. lanceolata leaf	ddMS3, AcquireX Deep Scan, ddMS2	99.3, 85.6, 90.1	4.113
N-({(2R,3S,4R,5S)-3,4-Dihydroxy-5-[2-(isopropylamino)-2-oxoethyl]tetrahydro-2-furanyl}methyl)-4-methoxybenzamide	P. lanceolata leaf	RTLS, AcquireX Deep Scan	97.1, 82.4	6.8
N-({(2R,4S,5R)-5-[1-Methyl-3-(2-naphthyl)-1H-pyrazol-5-yl]-1-azabicyclo[2.2.2]oct-2-yl}methyl)methanesulfonamide	P. lanceolata leaf	ddMS3, RTLS	83.6, 91.6	3.568
N-Acetyl-DL-norvaline	P. lanceolata leaf	RTLS, AcquireX Deep Scan, ddMS2	93.4, 64.3, 82.7	2.068
N-Acetyl-L-phenylalanine	P. lanceolata leaf	ddMS3, AcquireX Deep Scan	93.7, 67.1	0.548
N-Acetylsphingosine	P. lanceolata leaf	RTLS, AcquireX Deep Scan, ddMS2	82.8, 84.3, 79.2	11.83
NXJOCELNFPGKIV-UCYSXKDZSA-N	P. lanceolata leaf	RTLS, AcquireX Deep Scan, ddMS2	97.7, 98.0, 98.3	9.3

O-Benzoyl-L-malic acid	P. lanceolata leaf	ddMS3, RTLS, ddMS2	99.0, 98.2, 98.4	4.599
OLIGOMYCIN C	P. lanceolata leaf	ddMS3, RTLS	63.9, 99.1	15.287
ONFWNHLCKGVKGW-JCFRVBNTSA-N	P. lanceolata leaf	ddMS3, RTLS, ddMS2	89.2, 89.6, 87.7	10.069
Octanedioic acid	P. lanceolata leaf	ddMS3, RTLS, ddMS2	98.3, 98.6, 98.6	4.021
Oleic acid	P. lanceolata leaf	ddMS3, AcquireX Deep Scan, ddMS2	99.8, 72.4, 98.9	15.833
Oxohongdenafil	P. lanceolata leaf	ddMS3, AcquireX Deep Scan	92.5, 88.4	10.247
PRZVXHGUJJPSME-YLOFSNTESA-N	P. lanceolata leaf	RTLS, AcquireX Deep Scan	97.8, 96.5	2.102
Palmitoleic acid	P. lanceolata leaf	ddMS3, AcquireX Deep Scan, ddMS2	96.9, 85.9, 97.9	10.676
Phendimetrazine	P. lanceolata leaf	RTLS, AcquireX Deep Scan, ddMS2	80.2, 78.6, 77.1	2.013
Phytolaccosideb	P. lanceolata leaf	RTLS, AcquireX Deep Scan, ddMS2	94.8, 80.1, 70.9	5.8
Pimelic acid	P. lanceolata leaf	RTLS, AcquireX Deep Scan, ddMS2	92.2, 61.0, 76.3	3.049
QPRQJOHKNJIMGN-XXUZYDRNSA-N	P. lanceolata leaf	ddMS3, AcquireX Deep Scan	99.2, 99.1	12.403
Quercetin	Both	RTLS, AcquireX Deep Scan, ddMS2	82.8, 83.6, 75.1	4.342
RLGYLKXDNQBDCU-WPJUKMLSSA-N	P. lanceolata leaf	RTLS, AcquireX Deep Scan, ddMS2	99.8, 99.3, 99.5	5.048
RNPMSPOQHSSJPQ-UHFFFAOYSA-N	P. lanceolata leaf	RTLS, AcquireX Deep Scan, ddMS2	94.3, 96.1, 96.6	8.884
SB236057A	P. lanceolata leaf	ddMS3, AcquireX Deep Scan, ddMS2	86.7, 85.4, 83.6	12.739
SWYRVCGNMNAFEK-FIVCSZPPSA-N	P. lanceolata leaf	ddMS3, AcquireX Deep Scan, ddMS2	96.1, 94.4, 97.7	3.452
Salicylamide	P. lanceolata leaf	RTLS, AcquireX Deep Scan, ddMS2	80.0, 72.2, 73.1	2.314
Schisphenthin A	P. lanceolata leaf	ddMS3, AcquireX Deep Scan	80.2, 75.7	13.567
Scropheanoside I	P. lanceolata leaf	ddMS3, RTLS, ddMS2	99.8, 99.8, 99.8	5.369
Sorbitol	P. lanceolata leaf	RTLS, AcquireX Deep Scan	90.3, 87.9	0.45
Stearic acid	P. lanceolata leaf	ddMS3, AcquireX Deep Scan, ddMS2	99.7, 91.4, 99.0	11.845
Surfactin	P. lanceolata leaf	RTLS, AcquireX Deep Scan, ddMS2	88.1, 87.0, 89.2	14.121
Tazarotenic acid	P. lanceolata leaf	RTLS, AcquireX Deep Scan, ddMS2	88.5, 89.5, 87.8	2.992
Thr-Phe-Gln	P. lanceolata leaf	RTLS, AcquireX Deep Scan	98.0, 85.3	3.815
Trp-Ile-Asp	P. lanceolata leaf	RTLS, AcquireX Deep Scan, ddMS2	97.7, 70.2, 63.3	6.054
UHHVHDDICOEBTQ-AHBXVSLLSA-N	Both	RTLS, AcquireX Deep Scan	96.3, 80.2	2.67
Vidarabine	P. lanceolata leaf	ddMS3, RTLS, ddMS2	97.7, 99.5, 98.4	0.546
Villosolside	P. lanceolata leaf	RTLS, AcquireX Deep Scan, ddMS2	81.3, 75.3, 64.7	2.537

WYUMOABRAAEBAO-NOAJPBGCSA-N	P. lanceolata leaf	RTLS, AcquireX Deep Scan, ddMS2	99.8, 99.3, 99.8	4.789
XFCCRRLCYIVVJM-YNUKNDKESA-N	P. lanceolata leaf	RTLS, AcquireX Deep Scan, ddMS2	90.0, 76.3, 89.9	4.193
ZORMCMFEBIUIRM-UHFFFAOYSA-N	P. lanceolata leaf	ddMS3, AcquireX Deep Scan, ddMS2	81.8, 79.7, 82.8	14.19
[(2R,3R,4S,5S,6R)-2-(acetyloxymethyl)-3,5-dihydroxy-6-[(2S,3R)-2,3,4-trihydroxybutoxy]oxan-4-yl] hexanoate	P. lanceolata leaf	RTLS, AcquireX Deep Scan, ddMS2	99.5, 96.5, 95.1	3.478
[(2R,3S,4S,5R,6S)-3,4,5-trihydroxy-6-(4-hydroxyphenoxy)oxan-2-yl]methyl (2E)-3-(3,4-dihydroxyphenyl)prop-2-enoate	P. lanceolata leaf	ddMS3, AcquireX Deep Scan, ddMS2	84.6, 67.5, 86.7	4.533
[(2S,3R,4S,5S,6R)-2-(5,6-dimethoxy-6'-oxospiro[1,2-dihydroindene-3,3'-cyclohexa-1,4-diene]-4-yl)oxy-4,5-dihydroxy-6-(hydroxymethyl)oxan-3-yl] acetate	P. lanceolata leaf	RTLS, AcquireX Deep Scan, ddMS2	92.8, 92.7, 98.8	5.821
[(3R,4S)-1-(4-Morpholinylcarbonyl)-3-(2-{4-[3-(trifluoromethyl)phenyl]-1-piperazinyl}ethyl)-4-piperidinyl]acetic acid	P. lanceolata leaf	ddMS3, AcquireX Deep Scan, ddMS2	98.2, 96.2, 96.5	5.935
[3,4,5-Trihydroxy-6-[(5-hydroxy-4a-methyl-8-methylidene-2-propan-2-yl-1,2,3,4,5,6,7,8a-octahydronaphthalen-1-yl)oxy]oxan-2-yl]methyl acetate	P. lanceolata leaf	RTLS, AcquireX Deep Scan, ddMS2	80.1, 64.8, 63.1	8.432
[4-[(2S,3R,4S,5S,6R)-3,4,5-trihydroxy-6-(hydroxymethyl)oxan-2-yl]oxyphenyl]methyl 2-[(E)-3-(4-hydroxyphenyl)prop-2-enoyl]oxy-3-methyl-3-[(2S,3R,4S,5S,6R)-3,4,5-trihydroxy-6-(hydroxymethyl)oxan-2-yl]oxybutanoate	P. lanceolata leaf	ddMS3, AcquireX Deep Scan	98.9, 87.2	5.615
cis-5-Dodecene dioic acid	P. lanceolata leaf	RTLS, AcquireX Deep Scan, ddMS2	75.6, 80.0, 77.3	7.778
{[2-({[(2R,4S,5R)-5-(2-Methyl-6-phenyl-4-pyrimidinyl)-1-azabicyclo[2.2.2]oct-2-yl]methyl}amino)-2-oxoethyl]sulfanyl}acetic acid	P. lanceolata leaf	RTLS, AcquireX Deep Scan, ddMS2	95.2, 81.6, 84.8	1.085
(+/-)12(13)-DiHOME	Both	RTLS, ddMS2	96.1, 88.6	10.586
(10Z,13Z)-15,16-dihydroxyoctadeca-10,13-dienoic acid	P. lanceolata leaf	AcquireX Deep Scan, ddMS2	63.4, 98.7	9.538
(1R,9R)-N-(4-Methoxyphenyl)-6-oxo-3-[(phenylsulfonyl)amino]-7,11-diazatricyclo[7.3.1.02,7]trideca-2,4-diene-11-carboxamide	P. lanceolata leaf	RTLS, ddMS2	69.3, 95.6	2.289
(1R,9S)-5-(4-Chlorophenyl)-11-(4-fluorobenzoyl)-7,11-diazatricyclo[7.3.1.02,7]trideca-2,4-dien-6-one	P. lanceolata leaf	AcquireX Deep Scan, ddMS2	86.9, 95.9	4.056

(2,3,5-Trihydroxy-5-methoxycarbonylcyclohexyl) 3,4,5-trihydroxybenzoate	Both	RTLS	99.9	0.45
(2R)-7-Methoxy-3-oxo-3,4-dihydro-2H-1,4-benzoxazin-2-yl β-D-glucopyranoside	P. lanceolata leaf	RTLS	99.6	0.48
(2R,3S,4S,5R,6R)-2-(hydroxymethyl)-6-[[4-(2-hydroxypropan-2-yl)cyclohexen-1-yl]methoxy]oxane-3,4,5-triol	P. lanceolata leaf	AcquireX Deep Scan, ddMS2	99.8, 99.1	4.217
(2R,4aR,6aS,6bR,10S,12aR)-10-hydroxy-2-(hydroxymethyl)-2,6a,6b,9,9,12a-hexamethyl-1,3,4,5,6,6a,7,8,8a,10,11,12,13,14b-tetradecahydropicene-4a-carboxylic acid	P. lanceolata leaf	ddMS3	97.5	9.978
(3R)-8-hydroxy-3-methyl-5-[(2S,3R,4S,5S,6R)-3,4,5-trihydroxy-6-(hydroxymethyl)oxan-2-yl]oxy-3,4-dihydroisochromen-1-one	P. lanceolata leaf	ddMS3, ddMS2	97.1, 94.8	3.344
(5alpha,9alpha,10beta)-3-Oxokaurane-16,17,19-triol 17,19-diacetate	P. lanceolata leaf	AcquireX Deep Scan	95.0	6.729
(9E,11Z)-8-hydroxyoctadeca-9,11-dienoic acid	P. lanceolata leaf	RTLS	99.0	10.585
(R)-octopamine	P. lanceolata leaf	AcquireX Deep Scan	95.3	2.946
(Z)-6,9,10-trihydroxyoctadec-7-enoic acid	P. lanceolata leaf	RTLS	96.1	8.148
1-benzyl-3-(tert-butyl)-N-[4-(6-methyl-1,3-benzothiazol-2-yl)phenyl]-1H-pyrazole-5-carboxamide	P. lanceolata leaf	AcquireX Deep Scan, ddMS2	93.0, 95.1	5.609
2-Methylhippuric acid	P. lanceolata leaf	RTLS, ddMS2	95.3, 81.8	3.848
2-[(2R,4aS,8S,8aS)-8-{2-[(4aS,7R,8aR)-7-(1-Carboxyvinyl)-1-hydroxy-4a-methyl-2-oxo-1,2,4a,5,6,7,8,8a-octahydro-1-naphthalenyl]ethyl}-4a-methyl-7-oxo-1,2,3,4,4a,7,8,8a-octahydro-2-naphthalenyl]acrylic acid	P. lanceolata leaf	AcquireX Deep Scan, ddMS2	95.3, 96.0	7.799
3-Oxo-alpha-ionol glucoside	P. lanceolata leaf	ddMS3	97.8	4.684
4-Hydroxybenzoic acid	P. lanceolata leaf	ddMS3	95.5	2.325
4-Oxoproline	Both	RTLS, ddMS2	99.3, 99.6	0.562
6-Acetamidohexanoic acid	P. lanceolata leaf	AcquireX Deep Scan	99.2	2.769
9-Hydroxy-10,12-octadecadienoic acid	P. lanceolata leaf	RTLS, ddMS2	99.9, 82.6	14.442
9-Hydroxy-20-(hydroxymethyl)-1,2,8,9,15-pentamethyl-17-oxo-18-oxapentacyclo[12.9.0.02,11.05,10.015,21]tricos-11-ene-5,20-dicarboxylic acid	P. lanceolata leaf	RTLS	99.0	12.261
BARAUATXLYPTGX-RYXRMNFISA-N	P. lanceolata leaf	AcquireX Deep Scan, ddMS2	97.1, 96.3	6.512

BPSJMBKZSUTYNF-GXCUHXBUSA-N	P. lanceolata leaf	AcquireX Deep Scan	95.5	1.761
Baicalin	P. lanceolata leaf	AcquireX Deep Scan, ddMS2	97.1, 96.3	5.068
Benzyl alcohol	P. lanceolata leaf	AcquireX Deep Scan, ddMS2	96.2, 98.4	0.819
Capsianoside V methyl ester	P. lanceolata leaf	AcquireX Deep Scan	95.6	8.409
Cernuoside	Both	ddMS3, ddMS2	96.9, 96.2	5.175
Cyclocarioside B	P. lanceolata leaf	AcquireX Deep Scan	95.6	10.889
Cynaroside	P. lanceolata leaf	ddMS3	95.3	4.462
D-Fructose	P. lanceolata leaf	RTLS, ddMS2	95.7, 92.4	0.457
D-Maltose	P. lanceolata leaf	RTLS	99.7	0.452
D-Panthenol	Both	AcquireX Deep Scan	95.8	3.802
D-Phenylalanine	P. lanceolata leaf	AcquireX Deep Scan	96.4	0.978
DL-Glutamine	P. lanceolata leaf	RTLS	95.8	0.516
ETHYL PALMITATE	P. lanceolata leaf	RTLS	95.0	14.629
Erucamide	Both	ddMS3	97.0	14.486
Ferulic acid	P. lanceolata leaf	ddMS3, ddMS2	96.2, 96.7	6.365
GSOMKADVURIZLA-UHFFFAOYSA-N	P. lanceolata leaf	AcquireX Deep Scan, ddMS2	80.0, 98.5	4.161
GYFFKZTYYAFCTR-UCEFVAKBSA-N	P. lanceolata leaf	RTLS	99.5	3.049
Gastrodin	P. lanceolata leaf	AcquireX Deep Scan, ddMS2	84.5, 95.5	1.408
Globularin	P. lanceolata leaf	AcquireX Deep Scan	96.3	5.224
HZQSTMDYOHETCT-DRFAHUGVSA-N	P. lanceolata leaf	RTLS	96.3	5.861
Hexadecanamide	P. lanceolata leaf	ddMS3	95.6	12.135
Hydroxyacetildenafil	P. lanceolata leaf	RTLS	99.9	12.52
Isoferulic acid	P. lanceolata leaf	AcquireX Deep Scan	96.6	5.667
JVIDANAJZDBKRL-YABCALNSSA-N	P. lanceolata leaf	AcquireX Deep Scan	98.5	4.274
Kakisaponin A	P. lanceolata leaf	ddMS3, ddMS2	98.5, 98.7	5.73
Keracyanin	P. lanceolata leaf	AcquireX Deep Scan	96.6	4.502
L-(-)-Malic acid	Both	AcquireX Deep Scan, ddMS2	98.1, 98.2	0.526
L-DOPA methyl ester	P. lanceolata leaf	ddMS3	96.6	3.266
Lactitol	P. lanceolata leaf	RTLS, ddMS2	95.7, 87.3	0.426
Lys-Tyr-Pro	P. lanceolata leaf	ddMS3	97.4	2.228

Menisdaurin D	P. lanceolata leaf	AcquireX Deep Scan	95.3	0.554
Methyl-Hesperidin	P. lanceolata leaf	AcquireX Deep Scan	99.3	4.827
N-({(2R,3S,4R,5S)-3,4-Dihydroxy-5-[2-(4-methyl-1-piperidinyl)-2-oxoethyl]tetrahydro-2-furanyl}methyl)cyclopropanecarboxamide	Both	RTLS, ddMS2	98.1, 98.2	10.579
N2-(3-chlorophenyl)-3-amino-4-(3-pyridyl)-6,7-dihydro-5H-cyclopenta[b]thieno[3,2-e]pyridine-2-carboxamide	P. lanceolata leaf	RTLS	96.9	0.802
Neosaxitoxin	P. lanceolata leaf	AcquireX Deep Scan, ddMS2	97.6, 97.5	0.653
Nitrobenzene	P. lanceolata leaf	RTLS, ddMS2	96.4, 92.5	1.156
OLAMGHNQGZIWHZ-UWJZAGIXSA-N	P. lanceolata leaf	AcquireX Deep Scan	96.6	10.739
P-hydroxybenzoic acid	Both	AcquireX Deep Scan, ddMS2	98.2, 95.4	2.651
PPG Acrylate n7	P. lanceolata leaf	AcquireX Deep Scan	97.0	7.817
Palmitic acid	P. lanceolata leaf	AcquireX Deep Scan	99.9	7.608
Poliumoside	P. lanceolata leaf	RTLS, ddMS2	90.2, 95.8	4.541
Proscillaridin A	P. lanceolata leaf	AcquireX Deep Scan	96.4	10.624
Pyridoxine	P. lanceolata leaf	AcquireX Deep Scan, ddMS2	96.5, 78.7	0.517
Quercilicoside A	P. lanceolata leaf	RTLS, ddMS2	96.5, 95.7	5.489
RYVGCUJETSKZDU-YQHZJLNZSA-N	P. lanceolata leaf	AcquireX Deep Scan	96.7	4.01
Rutin	Both	RTLS, ddMS2	97.2, 83.2	4.192
Suberic acid	Both	AcquireX Deep Scan	97.2	2.114
Surfactin C2	P. lanceolata leaf	AcquireX Deep Scan, ddMS2	98.2, 98.3	14.118
TYDBFNAOFZIICW-UHFFFAOYSA-N	Both	ddMS3, ddMS2	90.0, 98.6	11.716
Tributylphosphine oxide	P. lanceolata leaf	AcquireX Deep Scan	97.3	7.886
Trifolin	P. lanceolata leaf	RTLS	95.6	4.631
UHHVHDDICOEBTQ-ANHSACEGSA-N	Both	RTLS, ddMS2	95.6, 68.2	2.599
UWKRNCNWJVCHGZ-SWKRQXMGSA-N	P. lanceolata leaf	RTLS	97.1	4.112
Uracil	P. lanceolata leaf	AcquireX Deep Scan	95.1	0.547
VHNBSWKVDGABEV-UHFFFAOYSA-N	P. lanceolata leaf	AcquireX Deep Scan, ddMS2	98.8, 98.4	4.992
Vindoline	P. lanceolata leaf	ddMS3, ddMS2	96.8, 92.6	11.292
XJONDDZJLUVAPY-UHFFFAOYSA-N	P. lanceolata leaf	AcquireX Deep Scan	98.6	4.985

XPLMUADTACCMDJ-OUHALVONSA-N	Both	RTLS	99.4	4.572
cis-7-Hexadecenoic acid	P. lanceolata leaf	RTLS, ddMS2	96.4, 95.1	10.295
trans-Petroselinic acid	P. lanceolata leaf	RTLS	99.7	13.502
(Z)-7,10-dihydroxyoctadec-8-enoic acid	P. ovata husk	ddMS3, RTLS, AcquireX Deep Scan, ddMS2	77.2, 92.7, 67.1, 97.2	7.486
4-Pyridoxic acid	P. ovata husk	ddMS3, RTLS, AcquireX Deep Scan, ddMS2	89.4, 79.5, 81.1, 75.2	0.716
5'-S-Methyl-5'-thioadenosine	P. ovata husk	ddMS3, RTLS, AcquireX Deep Scan, ddMS2	99.4, 97.9, 74.3, 98.0	1.569
8-Hydroxy-9,10-epoxystearic acid	P. ovata husk	ddMS3, RTLS, AcquireX Deep Scan, ddMS2	96.7, 96.7, 65.6, 96.8	11.386
Bazedoxifene	P. ovata husk	ddMS3, RTLS, AcquireX Deep Scan, ddMS2	92.7, 85.8, 72.3, 92.3	1.761
Glu-Pro-Cys	P. ovata husk	ddMS3, RTLS, AcquireX Deep Scan, ddMS2	99.1, 68.8, 60.4, 69.6	4.181
HQEBQNGGKFIFBW-UHFFFAOYSA-N	P. ovata husk	ddMS3, RTLS, AcquireX Deep Scan	86.9, 80.9, 66.6	0.753
N-(Carboxyacetyl)-L-tryptophan	P. ovata husk	ddMS3, RTLS, AcquireX Deep Scan, ddMS2	95.6, 74.8, 62.0, 95.5	4.414
N-Acetyl-DL-tryptophan	P. ovata husk	ddMS3, RTLS, AcquireX Deep Scan, ddMS2	94.5, 95.4, 72.9, 94.7	4.415
Phloionolic acid	P. ovata husk	ddMS3, RTLS, AcquireX Deep Scan, ddMS2	97.1, 97.2, 64.1, 94.5	7.482
Pyrogallol	P. ovata husk	ddMS3, RTLS, AcquireX Deep Scan, ddMS2	87.7, 90.1, 70.0, 89.3	0.479
Vitamin P	P. ovata husk	ddMS3, RTLS, AcquireX Deep Scan, ddMS2	99.6, 98.6, 61.3, 99.4	4.186
(8aR,12S,12aR)-12-Hydroxy-4-methyl-4,5,6,7,8,8a,12,12a-octahydro-2H-3-benzoxecine-2,9(1H)-dione	P. ovata husk	ddMS3, RTLS, ddMS2	66.4, 82.2, 74.3	4.896
(E)-3,10-Dihydroxy-4,9-dimethyldodec-6-enedioic acid	P. ovata husk	ddMS3, AcquireX Deep Scan, ddMS2	86.3, 85.1, 87.4	4.443
(E)-9,10-dihydroxytetradec-6-enedioic acid	P. ovata husk	ddMS3, RTLS, ddMS2	87.4, 92.9, 90.9	4.901
1,4-Anhydro-5-[(cyclopropylcarbonyl)amino]-2,5-dideoxy-2-(dimethylamino)-D-arabinitol	P. ovata husk	ddMS3, RTLS	90.5, 89.5	0.535
10-Nitrooleate	P. ovata husk	ddMS3, RTLS	91.5, 86.6	6.746
11-hydroxy-9-octadecenoic acid	P. ovata husk	ddMS3, RTLS	96.5, 86.3	10.837
12,16,18-Trihydroxy-7-(1-hydroxy-2,3-dimethylbutyl)-6,13-dimethylpentacyclo[10.8.0.02,9.05,9.013,18]icosa-1,19-dien-11-one	P. ovata husk	RTLS, AcquireX Deep Scan, ddMS2	78.7, 80.7, 66.5	8.362

2-(3,4-Dihydroxyphenyl)-5,7-dihydroxy-4-oxo-4H-chromen-3-yl 6-O-β-D-xylopyranosyl-β-D-glucopyranoside	P. ovata husk	ddMS3, RTLS, ddMS2	93.0, 77.4, 82.2	4.062
2-(3,4-Dihydroxyphenyl)ethyl pentopyranosyl-(1->2)-6-deoxyhexopyranosyl-(1->3)-4-O-[(2E)-3-(3,4-dihydroxyphenyl)-2-propenoyl]hexopyranoside	P. ovata husk	ddMS3, RTLS	95.2, 98.1	4.562
2-(5-Carboxypentanoylamino)benzoic acid	P. ovata husk	ddMS3, RTLS, ddMS2	82.5, 81.7, 73.8	2.501
2-Aminoadipic acid	P. ovata husk	ddMS3, RTLS, ddMS2	84.1, 86.2, 86.0	0.56
2-Furoic acid	P. ovata husk	ddMS3, RTLS, ddMS2	92.4, 87.1, 87.5	0.559
2-Octoxy-6-[(3,4,5-trihydroxyoxan-2-yl)oxymethyl]oxane-3,4,5-triol	P. ovata husk	ddMS3, RTLS, ddMS2	84.0, 91.0, 74.4	5.654
3,4-Dimethylbenzoic acid	P. ovata husk	ddMS3, RTLS	86.5, 89.1	1.322
3-Hydroxy-6-methylheptyl 2-O-?-D-glucopyranosyl-?-D-glucopyranoside	P. ovata husk	ddMS3, RTLS, ddMS2	99.6, 96.5, 97.3	3.33
3-Hydroxybenzyl alcohol	P. ovata husk	ddMS3, RTLS	84.6, 88.0	1.789
3-Methyladipic acid	P. ovata husk	ddMS3, RTLS, ddMS2	83.9, 94.7, 91.3	2.847
3-hydroxy-7-methoxyisobenzofuran-1(3H)-one	P. ovata husk	ddMS3, RTLS, ddMS2	93.5, 98.2, 65.0	4.54
4-Hydroxybenzoic acid glucoside	P. ovata husk	ddMS3, AcquireX Deep Scan, ddMS2	98.3, 72.5, 98.2	0.874
4-Hydroxycoumarin	P. ovata husk	ddMS3, RTLS	94.0, 64.4	4.539
4-[1-[(2R,3R,4S,5S,6R)-6-[[(2R,3R,4R)-3,4-dihydroxy-4-(hydroxymethyl)oxolan-2-yl]oxymethyl]-3,4,5-trihydroxyoxan-2-yl]oxyethyl]-5,5-dimethyloxolan-2-one	P. ovata husk	RTLS, AcquireX Deep Scan	96.4, 60.5	2.693
5-(5,7-Dihydroxy-3-methoxy-4-oxo-4H-chromen-2-yl)-2-hydroxyphenyl beta-D-xylopyranoside	P. ovata husk	ddMS3, RTLS, ddMS2	90.8, 82.4, 81.4	5.601
Adenosine	P. ovata husk	ddMS3, RTLS	99.7, 99.5	0.554
Avicularin	P. ovata husk	ddMS3, RTLS	99.2, 77.5	4.063
Benzyl 6-O-beta-D-glucopyranosyl-beta-D-glucopyranoside	P. ovata husk	ddMS3, RTLS, ddMS2	88.2, 90.4, 91.4	2.806
CEZKIFXYWPTANH-WLEOHLSHSA-N	P. ovata husk	ddMS3, RTLS, ddMS2	99.0, 70.5, 70.7	4.224
CJHYKSSBQRABTM-GEFUDKIDSA-N	P. ovata husk	ddMS3, RTLS, ddMS2	99.4, 96.3, 96.7	2.468
CLLRXUUNPKRYEF-UHFFFAOYSA-N	P. ovata husk	ddMS3, AcquireX Deep Scan	84.5, 60.6	3.412
D-(+)-Tryptophan	P. ovata husk	ddMS3, RTLS	98.4, 97.5	1.729
DL-2-(acetylamino)-3-phenylpropanoic acid	P. ovata husk	ddMS3, RTLS, ddMS2	97.6, 92.8, 97.9	3.886
Dodecyltrimethylammonium	P. ovata husk	ddMS3, RTLS, ddMS2	95.3, 94.2, 97.0	8.878
GODSXGJMZIRXOG-QAYRTFGPSA-N	P. ovata husk	ddMS3, RTLS, ddMS2	84.4, 83.3, 83.9	6.007

Glu-Leu	P. ovata husk	ddMS3, RTLS, ddMS2	75.7, 75.5, 82.4	1.638
Glu-Trp	P. ovata husk	ddMS3, RTLS, ddMS2	86.9, 73.9, 69.1	3.202
Glu-Tyr	P. ovata husk	ddMS3, RTLS, ddMS2	97.6, 72.5, 73.5	1.048
Gly-Ser-Trp	P. ovata husk	ddMS3, RTLS, ddMS2	95.5, 97.6, 97.1	0.693
Glycerophospho-N-palmitoyl ethanolamine	P. ovata husk	ddMS3, RTLS, ddMS2	88.2, 82.0, 84.5	9.937
Hydrocinnamic acid	P. ovata husk	ddMS3, AcquireX Deep Scan, ddMS2	63.5, 64.5, 85.6	1.786
Ile-Glu	P. ovata husk	ddMS3, RTLS	86.1, 84.8	1.634
Inosine	P. ovata husk	RTLS, AcquireX Deep Scan, ddMS2	88.1, 83.6, 83.4	0.555
Jasminoside N	P. ovata husk	RTLS, AcquireX Deep Scan	82.1, 65.9	6.006
KBTSKABIWIUJAS-GLVOFWRWSA-N	P. ovata husk	ddMS3, RTLS, ddMS2	84.6, 85.3, 79.9	4.924
KHCREUNEQGORAM-UHFFFAOYSA-N	P. ovata husk	ddMS3, RTLS, ddMS2	95.5, 88.7, 89.5	4.676
Kaempferol	P. ovata husk	ddMS2, RTLS	83.3, 94.5	5.953
L-Glutamic acid	P. ovata husk	RTLS, AcquireX Deep Scan, ddMS2	98.0, 95.3, 97.2	0.52
L-Iditol	P. ovata husk	ddMS3, RTLS	97.6, 97.4	0.612
L-Tyrosine	P. ovata husk	ddMS3, RTLS	99.2, 83.4	0.556
Lactose	P. ovata husk	ddMS3, RTLS	99.6, 98.1	0.456
Luteolin 8-glucoside	P. ovata husk	ddMS3, RTLS	87.1, 89.3	3.783
Malonylgenistin	P. ovata husk	ddMS3, RTLS, ddMS2	97.6, 96.4, 95.1	5.556
Multinoside A	P. ovata husk	ddMS3, RTLS	99.5, 90.5	4.182
N1-phenethylbenzene-1-carbothioamide	P. ovata husk	ddMS3, RTLS, ddMS2	89.3, 84.3, 89.9	1.141
NBQPHANHNTWDML-QRDUDCRASA-N	P. ovata husk	ddMS3, RTLS, ddMS2	97.5, 92.0, 90.1	4.658
NKFZLEYLWAFYEH-WQYJSJMCSA-N	P. ovata husk	ddMS3, RTLS	94.6, 95.1	4.287
Nepitrin	P. ovata husk	ddMS3, RTLS, ddMS2	94.5, 60.5, 85.1	4.852
Nipecotic acid	P. ovata husk	ddMS3, RTLS	73.8, 93.8	0.509
PNUZTGLWURTQTO-UHFFFAOYSA-N	P. ovata husk	ddMS3, RTLS, ddMS2	91.7, 93.2, 92.7	5.204
PPVPALXLGVOWHV-DUXPYHPUSA-N	P. ovata husk	ddMS3, RTLS, ddMS2	90.3, 89.3, 87.8	1.385
Pantothenic acid	P. ovata husk	ddMS3, RTLS	91.2, 93.1	1.146
Pelargonidin	P. ovata husk	ddMS3, RTLS	94.2, 96.6	6.631
Phloretic acid	P. ovata husk	ddMS3, RTLS	88.1, 73.6	1.796
Phloroglucinol	P. ovata husk	ddMS3, RTLS	97.4, 96.9	1.619
Qercetin3'-glucoside	P. ovata husk	ddMS3, RTLS	99.2, 99.4	4.194
Revefenacin	P. ovata husk	ddMS3, RTLS, ddMS2	89.6, 78.7, 89.5	7.04
Sinapine	P. ovata husk	ddMS3, RTLS, ddMS2	98.2, 96.0, 98.0	3.161
Sophoricoside	P. ovata husk	ddMS3, RTLS	98.7, 98.0	4.953
Tyr-Glu	P. ovata husk	ddMS3, RTLS	81.3, 83.0	1.054

UDWUZPSSUIWBKB-BALKYSKWSA-N	P. ovata husk	ddMS3, RTLS	86.0, 67.6	4.492
UTECWQIXBMWRRR-UHFFFAOYSA-N	P. ovata husk	ddMS3, RTLS, ddMS2	99.2, 93.7, 95.9	3.69
Umbelliferone 7-O-Rutinoside	P. ovata husk	ddMS3, RTLS, ddMS2	79.5, 82.7, 66.7	4.3
WYVSACLCCJHHGJ-YVIDGVALSA-N	P. ovata husk	ddMS3, RTLS, ddMS2	81.1, 77.9, 91.7	4.206
ZTBNLEYFQBESKP-DRRMYKHWSA-N	P. ovata husk	ddMS3, RTLS, ddMS2	97.1, 99.3, 98.9	1.611
p-hydroxyphenylpropylene glycol	P. ovata husk	ddMS3, RTLS, ddMS2	80.8, 81.5, 73.7	1.388
trans-3-Indoleacrylic acid	P. ovata husk	ddMS3, RTLS, ddMS2	98.0, 84.5, 84.1	1.729
α-Eleostearic acid	P. ovata husk	ddMS3, RTLS	92.9, 86.1	9.936
(1R,9R)-5-[(Benzylsulfonyl)amino]-N-(4-methoxyphenyl)-6-oxo-7,11-diazatricyclo[7.3.1.02,7]trideca-2,4-diene-11-carboxamide	P. ovata husk	AcquireX Deep Scan, ddMS2	91.7, 95.1	0.785
(1S)-1,5-Anhydro-2,3,6-trideoxy-6-(4-piperidinylamino)-1-{[5-(2-pyridinyl)-1,2-oxazol-3-yl]methyl}-D-erythro-hexitol	P. ovata husk	RTLS, ddMS2	99.4, 97.1	5.213
(1ξ)-1,5-Anhydro-1-[(2R,3R)-3,5,7-trihydroxy-2-(4-hydroxyphenyl)-4-oxo-3,4-dihydro-2H-chromen-6-yl]-D-glucitol	P. ovata husk	RTLS	98.7	5.139
1-Deoxypebrolide	P. ovata husk	ddMS3	98.8	9.534
2,6-Diaminotoluene	P. ovata husk	AcquireX Deep Scan	95.9	15.879
2-(3,4-Dihydroxyphenyl)-5-hydroxy-7-methoxy-4-oxo-4H-chromen-3-yl 2-O-β-D-xylopyranosyl-β-D-glucopyranoside	P. ovata husk	ddMS3, ddMS2	99.3, 61.9	5.184
3'-Adenosine monophosphate (3'-AMP)	P. ovata husk	AcquireX Deep Scan	97.5	0.519
3,4,5-Trimethoxyphenyl 6-O-pentopyranosyl-β-D-glucopyranoside	P. ovata husk	RTLS, ddMS2	93.6, 95.4	1.473
3-[(2S,5aS,8aR)-1-Methyl-5-oxo-6-(2-thienylmethyl)decahydropyrrolo[3,2-E][1,4]diazepin-2-yl]-N-(2-thienylmethyl)propanamide	P. ovata husk	ddMS3	97.2	2.818
4-Hydroxy-2-(2-hydroxypropan-2-yl)-7-methylfuro[3,2-g]chromen-5-one	P. ovata husk	AcquireX Deep Scan, ddMS2	80.4, 95.2	1.381
6-O-Acetylastragalin	P. ovata husk	RTLS	95.5	5.235
Apigetrin	P. ovata husk	RTLS, ddMS2	90.3, 96.0	5.221
Asp-Trp-Ser	P. ovata husk	ddMS3	97.1	4.052
Barrelin	P. ovata husk	ddMS3	97.2	2.602
Bis(2-ethylhexyl) amine	P. ovata husk	ddMS3	96.0	9.04
Bis(4-ethylbenzylidene)sorbitol	P. ovata husk	RTLS	98.3	9.962
CNLUHMGCIFZWFM-IKCJRXJDSA-N	P. ovata husk	ddMS3	95.9	4.21
D-(+)-Maltose	P. ovata husk	RTLS	99.1	0.542

DUXQKCCELUKXOE-UHFFFAOYSA-N	P. ovata husk	AcquireX Deep Scan, ddMS2	97.2, 90.2	4.537
Dehydrologanin	P. ovata husk	RTLS	95.2	3.097
Dihexylamine	P. ovata husk	ddMS3	96.7	4.941
Gomisin M1	P. ovata husk	ddMS3, ddMS2	96.9, 96.8	9.443
Guanosine	P. ovata husk	RTLS, ddMS2	98.5, 99.0	0.721
Haloperidol	P. ovata husk	AcquireX Deep Scan	97.5	6.298
Hexanoylglycine	P. ovata husk	ddMS3	95.6	2.688
Hymecromone	P. ovata husk	ddMS3, ddMS2	99.2, 71.9	1.325
Itopride	P. ovata husk	RTLS, ddMS2	99.0, 70.9	12.139
KZIIWRHNDHNGOM-FOIOJJRYSA-N	P. ovata husk	RTLS	97.0	5.797
Kaempferol 3-b-laminaribioside	P. ovata husk	RTLS	95.6	4.285
Kaempferol-7-O-glucoside	P. ovata husk	RTLS	99.5	4.644
L-Arginine	P. ovata husk	RTLS	99.5	0.442
L-Asparagine	P. ovata husk	RTLS	96.4	0.516
Loperamide	P. ovata husk	RTLS, ddMS2	96.7, 92.2	5.755
Lys-Glu-Tyr	P. ovata husk	ddMS3, ddMS2	99.5, 99.5	4.331
Methyl (6S)-3-benzyl-5-{[5-(dimethylamino)-1-naphthyl]sulfonyl}-4,5,6,7-tetrahydro-3H-imidazo[4,5-c]pyridine-6-carboxylate	P. ovata husk	AcquireX Deep Scan, ddMS2	88.0, 96.5	1.554
N-Acetyl-D-alloisoleucine	P. ovata husk	RTLS	97.1	3.758
N-Methyl-2-pyrrolidone	P. ovata husk	RTLS	96.0	1.175
N-Octyl-2-pyrrolidone	P. ovata husk	RTLS	96.8	9.928
N1-Benzyl-2-[(2-oxo-3-piperidyl)carbonyl]hydrazine-1-carbothioamide	P. ovata husk	AcquireX Deep Scan	96.8	0.504
Naphazoline	P. ovata husk	AcquireX Deep Scan	95.6	3.766
Phe-Glu	P. ovata husk	ddMS3	97.4	2.47
Procyclidine	P. ovata husk	AcquireX Deep Scan	97.4	6.32
Proline	P. ovata husk	RTLS, ddMS2	95.0, 95.1	0.629
Pyrene	P. ovata husk	AcquireX Deep Scan	96.0	1.869
QZPMJUUYIRZJKD-UHFFFAOYSA-N	P. ovata husk	AcquireX Deep Scan, ddMS2	61.1, 97.0	1.369
Quercetin 3-O-rhamnoside-7-O-glucoside	P. ovata husk	RTLS	99.3	4.42
SIDQWGBLTYBENT-UHFFFAOYSA-N	P. ovata husk	ddMS3	96.9	1.797
Telmisartan	P. ovata husk	AcquireX Deep Scan	99.5	6.773
Trp-Asp-Ser	P. ovata husk	ddMS3	97.3	3.956
VRHJVSQWZHBYED-KLLQSXCASA-N	P. ovata husk	RTLS	98.7	4.836
WYMOHLOJGOGUOX-WPTNZZQCSA-N	P. ovata husk	RTLS, ddMS2	98.2, 98.2	11.13
α,α-Trehalose	P. ovata husk	RTLS	97.1	0.537
(12Z,15Z)-9,10,11-Trihydroxy-12,15-octadecadienoic acid	P. lanceolata leaf	ddMS3, RTLS, AcquireX Deep Scan, ddMS2	95.5, 98.4, 90.6, 94.7	7.682
(15Z)-9,12,13-Trihydroxy-15-octadecenoic acid	P. lanceolata leaf	ddMS3, RTLS, AcquireX Deep Scan	98.8, 95.1, 88.4	7.15
(1R,9R)-N-(4-Acetylphenyl)-5-{[(4-methoxyphenyl)sulfonyl]amino}-	P. lanceolata leaf	ddMS3, RTLS, AcquireX Deep Scan, ddMS2	95.5, 90.9, 89.5, 95.1	1.353

6-oxo-7,11-diazatricyclo[7.3.1.02,7]trideca-2,4-diene-11-carboxamide				
(1S,4aS,5S)-1,4a-dimethyl-6-methylidene-5-[(E)-3-oxobut-1-enyl]-3,4,5,7,8,8a-hexahydro-2H-naphthalene-1-carboxylic acid	P. lanceolata leaf	ddMS3, RTLS, AcquireX Deep Scan, ddMS2	76.9, 96.6, 72.7, 95.7	7.764
(2R,3R,4S,5S,6R)-2-(3-methylbutoxy)-6-[[(2S,3R,4S,5R)-3,4,5-trihydroxyoxan-2-yl]oxymethyl]oxane-3,4,5-triol	P. lanceolata leaf	ddMS3, RTLS, AcquireX Deep Scan, ddMS2	90.0, 85.7, 73.2, 87.9	3.519
(2S)-pyrrolidinium-2-carboxylate	Both	ddMS3, RTLS, AcquireX Deep Scan, ddMS2	99.8, 99.8, 99.7, 99.9	0.455
(2S,3R,4S,5R)-2-{[(2R,3R,4S,5S,6R)-4,5-dihydroxy-6-(hydroxymethyl)-2-(2-phenylethoxy)oxan-3-yl]oxy}oxane-3,4,5-triol	P. lanceolata leaf	ddMS3, RTLS, AcquireX Deep Scan, ddMS2	93.1, 92.7, 76.9, 89.1	3.805
(2S,3R,4S,5S,6R)-2-[(6E)-3,10-dihydroxy-2,6,10-trimethyldodeca-6,11-dien-2-yl]oxy-6-(hydroxymethyl)oxane-3,4,5-triol	P. lanceolata leaf	ddMS3, RTLS, AcquireX Deep Scan	99.6, 99.7, 99.6	5.178
(2S,3S,4S,5R,6R)-3,4,5-trihydroxy-6-[2-hydroxy-3-[(9Z,12Z)-octadeca-9,12-dienoyl]oxypropoxy]oxane-2-carboxylic acid	P. lanceolata leaf	ddMS3, RTLS, AcquireX Deep Scan, ddMS2	99.4, 99.5, 98.6, 99.2	8.328
(2S,3S,4S,5R,6S)-3,4,5-trihydroxy-6-[5-hydroxy-2-(2-hydroxyphenyl)-6-methoxy-4-oxochromen-7-yl]oxyoxane-2-carboxylic acid	P. lanceolata leaf	ddMS3, RTLS, AcquireX Deep Scan, ddMS2	87.1, 87.8, 88.7, 87.4	5.099
(2S,5aS,8aR)-6-Benzyl-1-methyl-2-[3-(4-morpholinyl)-3-oxopropyl]octahydropyrrolo[3,2-E][1,4]diazepin-5(2H)-one	P. lanceolata leaf	ddMS3, RTLS, AcquireX Deep Scan, ddMS2	99.6, 95.5, 98.7, 99.2	6.367
(3R,4S,5S,6R)-2-[4-(2-hydroxyethyl)phenoxy]-6-(hydroxymethyl)oxane-3,4,5-triol	Both	ddMS3, RTLS, AcquireX Deep Scan, ddMS2	98.7, 97.2, 99.5, 99.3	2.183
(4E,6E)-2,7-dimethyl-8-[(2R,3R,4S,5S,6R)-3,4,5-trihydroxy-6-(hydroxymethyl)oxan-2-yl]oxyocta-4,6-dienoic acid	Both	ddMS3, RTLS, AcquireX Deep Scan, ddMS2	90.8, 95.0, 74.4, 65.7	4.403
(4aR)-5-hydroxy-6-methoxy-1,1-dimethyl-7-propan-2-yl-2,3,4,9,10,10a-hexahydrophenanthrene-4a-carboxylic acid	P. lanceolata leaf	ddMS3, RTLS, AcquireX Deep Scan, ddMS2	95.1, 94.4, 94.1, 95.3	11.529
(4aS,7aR)-3-(3-Fluorophenyl)-2,4-dioxo-N-propyl-1-(2-thienylmethyl)octahydro-5H-pyrrolo[3,2-d]pyrimidine-5-carboxamide	P. lanceolata leaf	ddMS3, RTLS, AcquireX Deep Scan, ddMS2	99.1, 96.3, 99.0, 98.1	3.784
(5E)-3,4,9-trihydroxy-2-propyl-2,3,4,7,8,9-hexahydrooxecin-10-one	Both	ddMS3, RTLS, AcquireX Deep Scan, ddMS2	98.2, 97.7, 95.9, 97.9	5.536
(5E,9E)-4,8-dihydroxy-5,9,13,14-tetramethyl-1-oxacyclotetradeca-5,9-dien-2-one	P. lanceolata leaf	ddMS3, RTLS, AcquireX Deep Scan, ddMS2	86.4, 88.2, 76.2, 88.9	5.925

(6E,8E)-5,10-dioxooctadeca-6,8-dienoic acid	P. lanceolata leaf	ddMS3, RTLS, AcquireX Deep Scan, ddMS2	82.4, 81.7, 83.7, 80.1	8.022
(9R,10E,12Z,15Z)-9-hydroxyoctadeca-10,12,15-trienoic acid	Both	ddMS3, RTLS, AcquireX Deep Scan	96.2, 96.1, 91.4	10.103
(9Z,12Z)-7,8,16-trihydroxyoctadeca-9,12-dienoic acid	P. lanceolata leaf	ddMS3, RTLS, AcquireX Deep Scan, ddMS2	97.3, 96.6, 95.3, 96.6	9.086
(E)-8,9,10-trihydroxyoctadec-6-enoic acid	Both	ddMS3, RTLS, AcquireX Deep Scan	98.6, 99.0, 85.1	7.143
(R)-Bitalin A	P. lanceolata leaf	ddMS3, RTLS, AcquireX Deep Scan, ddMS2	79.4, 81.4, 70.7, 80.1	4.157
(Rac)-Idroxioleic acid	Both	ddMS3, RTLS, AcquireX Deep Scan, ddMS2	88.2, 86.3, 89.2, 86.9	8.347
1,4:3,6-Dianhydro-2-(benzylamino)-5-[4-(cyclohexylmethyl)-1H-1,2,3-triazol-1-yl]-2,5-dideoxy-L-iditol	P. lanceolata leaf	ddMS3, RTLS, AcquireX Deep Scan, ddMS2	99.5, 98.2, 99.6, 99.5	7.458
1,4:3,6-Dianhydro-2-{[4-(4-biphenylyl)-2-pyrimidinyl]amino}-2,5-dideoxy-5-[(2-thienylcarbonyl)amino]-L-iditol	P. lanceolata leaf	ddMS3, RTLS, AcquireX Deep Scan, ddMS2	92.3, 99.3, 91.8, 92.1	3.561
1-(4-benzylpiperazino)-2-(pyridin-2-ylamino)propan-1-one	P. lanceolata leaf	ddMS3, RTLS, AcquireX Deep Scan, ddMS2	99.4, 76.2, 95.2, 98.2	8.184
1-O-Cinnamoylglucose	Both	ddMS3, RTLS, AcquireX Deep Scan, ddMS2	82.9, 85.6, 63.9, 86.0	0.766
1-Octen-3-yl primeveroside	P. lanceolata leaf	ddMS3, RTLS, AcquireX Deep Scan	87.4, 87.4, 92.1	5.388
11-(2-Hydroxy-3,4-dimethyl-5-oxofuran-2-yl)undecanoic acid	P. lanceolata leaf	ddMS3, RTLS, AcquireX Deep Scan, ddMS2	95.3, 94.7, 85.6, 84.2	8.416
11-(2-Methoxy-3,4-dimethyl-5-oxofuran-2-yl)undecanoic acid	P. lanceolata leaf	ddMS3, RTLS, AcquireX Deep Scan, ddMS2	79.6, 67.2, 81.1, 97.2	8.03
12-Oxo phytodienoic acid	Both	ddMS3, RTLS, AcquireX Deep Scan, ddMS2	97.1, 97.3, 90.3, 96.6	6.773
13(S)-HOTrE	Both	ddMS3, RTLS, AcquireX Deep Scan, ddMS2	99.2, 99.2, 99.0, 99.3	7.143
19-Norandrostenedione	P. lanceolata leaf	ddMS3, RTLS, AcquireX Deep Scan, ddMS2	78.9, 80.3, 78.6, 78.9	8.197
2',5,6',7-tetrahydroxyflavone	P. lanceolata leaf	ddMS3, RTLS, AcquireX Deep Scan, ddMS2	86.4, 96.4, 85.4, 81.5	5.399
2,3,4,5,6-pentahydroxyhexyl (9Z,12Z)-octadeca-9,12-dienoate	P. lanceolata leaf	ddMS3, RTLS, AcquireX Deep Scan, ddMS2	99.8, 99.7, 99.8, 99.8	10.715
2,3,4,9-Tetrahydro-1H-β-carboline-3-carboxylic acid	Both	ddMS3, RTLS, AcquireX Deep Scan, ddMS2	93.5, 94.9, 93.3, 90.9	2.688
2,3-Dinor prostaglandin E1	P. lanceolata leaf	ddMS3, RTLS, AcquireX Deep Scan, ddMS2	82.1, 82.8, 80.5, 76.7	6.531

2,3-Dinor-11β-prostaglandin F2α	P. lanceolata leaf	ddMS3, RTLS, AcquireX Deep Scan	96.0, 85.7, 64.4	7.759
2-(3,4-Dihydroxyphenyl)ethyl 3-O-(6-deoxy-β-L-mannopyranosyl)-6-O-[(2E)-3-(3,4-dihydroxyphenyl)-2-propenoyl]-β-D-glucopyranoside	Both	ddMS3, RTLS, AcquireX Deep Scan, ddMS2	89.2, 91.2, 91.2, 90.4	4.533
2-(hydroxymethyl)-6-[(E)-4-(1,2,4-trihydroxy-2,6,6-trimethylcyclohexyl)but-3-en-2-yl]oxyoxane-3,4,5-triol	P. lanceolata leaf	ddMS3, RTLS, AcquireX Deep Scan, ddMS2	97.5, 96.1, 98.5, 97.2	2.796
2-Amino-1,3,4-octadecanetriol	Both	ddMS3, RTLS, AcquireX Deep Scan	90.5, 91.2, 91.2	7.701
2-Hydroxycaproic acid	P. lanceolata leaf	ddMS3, RTLS, AcquireX Deep Scan, ddMS2	98.5, 93.4, 98.6, 98.6	2.962
2-Hydroxysebacic acid	P. lanceolata leaf	ddMS3, RTLS, AcquireX Deep Scan, ddMS2	92.4, 95.3, 88.6, 93.1	4.411
2-Isopropylmalic acid	Both	ddMS3, RTLS, AcquireX Deep Scan, ddMS2	92.8, 83.0, 94.3, 92.6	2.271
2-Methoxy-N-({(2R,4S,5R)-5-[3-(4-methoxyphenyl)-1-methyl-1H-pyrazol-5-yl]-1-azabicyclo[2.2.2]oct-2-yl}methyl)acetamide	P. lanceolata leaf	ddMS3, RTLS, AcquireX Deep Scan, ddMS2	99.1, 99.1, 98.8, 99.1	6.729
2-[3-Oxo-2-[4-[3,4,5-trihydroxy-6-(hydroxymethyl)oxan-2-yl]oxypentyl]cyclopentyl]acetic acid	P. lanceolata leaf	ddMS3, RTLS, AcquireX Deep Scan, ddMS2	93.9, 94.5, 76.6, 95.5	5.357
2-hydroxy-5-[3,4,5-trihydroxy-6-(hydroxymethyl)oxan-2-yl]oxybenzoic acid	Both	ddMS3, RTLS, AcquireX Deep Scan, ddMS2	97.7, 98.8, 98.1, 96.4	1.171
3-Hexadec-15-en-7-ynyl-4-hydroxy-5-methyloxolan-2-one	P. lanceolata leaf	ddMS3, RTLS, AcquireX Deep Scan	93.9, 92.5, 93.8	14.132
3-Hydroxystigmast-5-en-7-one	P. lanceolata leaf	ddMS3, RTLS, AcquireX Deep Scan, ddMS2	95.6, 87.7, 88.2, 94.5	14.38
3-Methoxy-4-[3,4,5-trihydroxy-6-(hydroxymethyl)oxan-2-yl]oxybenzoic acid	P. lanceolata leaf	ddMS3, RTLS, AcquireX Deep Scan, ddMS2	96.0, 94.6, 98.3, 95.9	1.263
3-Methylidene-7-propan-2-yldodecane-1,2-diol	P. lanceolata leaf	ddMS3, RTLS, AcquireX Deep Scan, ddMS2	98.3, 99.8, 98.6, 99.1	11.182
3-[2-[3,4,5-Trihydroxy-6-(hydroxymethyl)oxan-2-yl]oxyphenyl]prop-2-enoic acid	Both	ddMS3, RTLS, AcquireX Deep Scan, ddMS2	97.3, 92.5, 99.3, 95.8	2.897
3-[3-(beta-D-Glucopyranosyloxy)-2-methoxyphenyl]propanoic acid	P. lanceolata leaf	ddMS3, RTLS, AcquireX Deep Scan, ddMS2	96.2, 98.2, 96.9, 98.0	2.836
3-heptyl-3,6-dihydro-1H-furo[3,4-c]furan-4-one	P. lanceolata leaf	ddMS3, RTLS, AcquireX Deep Scan, ddMS2	80.3, 78.9, 63.3, 77.9	6.064
4-(3-Hydroxybutyl)phenyl β-D-glucopyranoside	P. lanceolata leaf	ddMS3, RTLS, AcquireX Deep Scan, ddMS2	93.4, 93.2, 90.6, 94.8	5.009
4-(beta-D-Glucopyranosyloxy)phenylacetic acid	P. lanceolata leaf	ddMS3, RTLS, AcquireX Deep Scan, ddMS2	90.8, 87.1, 83.4, 82.8	1.506

4-Acetamidobutanoic acid	P. lanceolata leaf	ddMS3, RTLS, AcquireX Deep Scan, ddMS2	83.5, 90.7, 82.4, 86.5	1.59
4-Hydroxybenzaldehyde	Both	ddMS3, RTLS, AcquireX Deep Scan, ddMS2	98.2, 98.5, 61.2, 98.1	3.231
4-Hydroxycinnamic acid	P. lanceolata leaf	ddMS3, RTLS, AcquireX Deep Scan, ddMS2	99.0, 87.2, 77.4, 71.9	3.949
4-Indolecarbaldehyde	P. lanceolata leaf	ddMS3, RTLS, AcquireX Deep Scan, ddMS2	98.8, 80.2, 98.4, 98.7	4.698
4-Methyl-2-(2-methylpropanoyloxy)-3-undecanoyloxypentanoic acid	P. lanceolata leaf	ddMS3, RTLS, AcquireX Deep Scan, ddMS2	99.6, 99.5, 99.6, 99.5	8.153
4-Methylquinoline	P. lanceolata leaf	ddMS3, RTLS, AcquireX Deep Scan, ddMS2	83.0, 91.3, 74.9, 76.4	2.686
4-O-beta-D-glucosyl-4-coumaric acid	P. lanceolata leaf	ddMS3, RTLS, AcquireX Deep Scan, ddMS2	97.4, 97.8, 97.4, 97.7	2.893
4-hydroxy-3-[(2S,3R,4S,5S,6R)-3,4,5-trihydroxy-6-(3-methylbutanoyloxymethyl)oxan-2-yl]oxybenzoic acid	P. lanceolata leaf	ddMS3, RTLS, AcquireX Deep Scan, ddMS2	74.6, 94.3, 81.8, 92.6	4.053
5,5-dimethyl-4-[(E)-3-methyl-7-[(2R,3R,4S,5S,6R)-3,4,5-trihydroxy-6-(hydroxymethyl)oxan-2-yl]oxyoct-3-enyl]oxolan-2-one	P. lanceolata leaf	ddMS3, RTLS, AcquireX Deep Scan, ddMS2	97.0, 97.3, 92.3, 98.1	5.122
5-p-Coumaroylquinic acid, (Z)-	P. lanceolata leaf	ddMS3, RTLS, AcquireX Deep Scan, ddMS2	95.9, 98.0, 86.6, 93.0	3.933
6-hydroxy-4,4,7a-trimethyl-6,7-dihydro-5H-1-benzofuran-2-one	P. lanceolata leaf	ddMS3, RTLS, AcquireX Deep Scan, ddMS2	85.5, 86.1, 85.7, 88.2	4.521
7-[2-(1-hydroxyhexyl)-3,6-dihydro-2H-pyran-6-yl]heptanoic acid	P. lanceolata leaf	ddMS3, RTLS, AcquireX Deep Scan, ddMS2	90.8, 74.6, 90.3, 88.8	8.757
8,9-Dimethoxy-2-(2-phenyldiazenyl)-3-(2-thienyl)-5,6-dihydropyrrolo[2,1-a]isoquinoline-1-carbonitrile	P. lanceolata leaf	ddMS3, RTLS, AcquireX Deep Scan	98.7, 95.9, 64.1	2.722
8-Hydroxyquinoline	P. lanceolata leaf	ddMS3, RTLS, AcquireX Deep Scan, ddMS2	80.5, 93.2, 80.4, 78.9	4.696
9(Z),11(E)-Conjugated linoleic acid	P. lanceolata leaf	ddMS3, RTLS, AcquireX Deep Scan, ddMS2	100.0, 99.8, 99.9, 99.9	10.714
9-(2,3-dihydroxypropoxy)-9-oxononanoic acid	Both	ddMS3, RTLS, AcquireX Deep Scan, ddMS2	94.1, 95.5, 66.3, 94.7	13.58
9-oxooctadeca-10,12-dienoic acid	Both	ddMS3, RTLS, AcquireX Deep Scan, ddMS2	96.9, 97.8, 98.0, 97.2	10.938
ADB-PINACA	P. lanceolata leaf	ddMS3, RTLS, AcquireX Deep Scan, ddMS2	99.4, 99.2, 96.4, 99.5	5.418
AWTYKUNFPBFFHC-UHFFFAOYSA-N	P. lanceolata leaf	ddMS3, RTLS, AcquireX Deep Scan	98.7, 98.6, 98.4	3.931

Adenine	Both	ddMS3, RTLS, AcquireX Deep Scan, ddMS2	99.5, 99.1, 98.2, 98.8	0.47
Agnuside	P. lanceolata leaf	ddMS3, RTLS, AcquireX Deep Scan, ddMS2	99.3, 99.6, 98.5, 99.6	4.127
Ancymidol	P. lanceolata leaf	ddMS3, RTLS, AcquireX Deep Scan, ddMS2	85.0, 86.3, 88.2, 83.3	6.15
Apigenin	P. lanceolata leaf	ddMS3, RTLS, AcquireX Deep Scan, ddMS2	99.5, 99.3, 99.4, 99.4	6.63
Arachidoyl glycine	P. lanceolata leaf	ddMS3, RTLS, AcquireX Deep Scan, ddMS2	84.3, 85.5, 80.1, 84.0	13.26
BMOKZWFNXYQOGE-REKXUBECSA-N	P. lanceolata leaf	ddMS3, RTLS, AcquireX Deep Scan, ddMS2	73.8, 72.5, 99.0, 99.0	5.371
BUKBJIBZOCTYLQ-UQTFRHSQSA-N	P. lanceolata leaf	ddMS3, RTLS, AcquireX Deep Scan, ddMS2	92.0, 94.3, 79.5, 89.8	12.949
Baldaccioside	P. lanceolata leaf	ddMS3, RTLS, AcquireX Deep Scan, ddMS2	97.2, 83.8, 84.0, 89.9	5.628
Betaine	P. lanceolata leaf	ddMS3, RTLS, AcquireX Deep Scan, ddMS2	95.7, 95.0, 94.7, 95.0	12.75
Bioside	Both	ddMS3, RTLS, AcquireX Deep Scan, ddMS2	95.1, 94.8, 93.3, 94.7	1.915
CAIKQNWPWSTGHN-CXNFFOGLSA-N	Both	ddMS3, RTLS, AcquireX Deep Scan, ddMS2	98.3, 97.1, 97.8, 99.9	6.773
CB-25	P. lanceolata leaf	ddMS3, RTLS, AcquireX Deep Scan, ddMS2	90.2, 90.6, 80.8, 91.3	13.327
CBJNLOVRAFQEQH-XTAVSMKVSA-N	P. lanceolata leaf	ddMS3, RTLS, AcquireX Deep Scan, ddMS2	99.8, 99.7, 99.8, 99.8	5.169
CCTTLCTYXPWGOMY-JKUWKVMRSA-N	P. lanceolata leaf	ddMS3, RTLS, AcquireX Deep Scan, ddMS2	99.2, 98.3, 97.3, 99.5	4.409
Caffeic acid	Both	ddMS3, RTLS, AcquireX Deep Scan, ddMS2	94.3, 94.1, 94.3, 94.4	4.767
Carbofuranphenol-3-keto	P. lanceolata leaf	ddMS3, RTLS, AcquireX Deep Scan, ddMS2	77.4, 82.5, 68.8, 74.8	2.082
Cassifolioside	Both	ddMS3, RTLS, AcquireX Deep Scan, ddMS2	98.7, 85.1, 96.4, 98.7	4.646
Chlorogenic acid	P. lanceolata leaf	ddMS3, RTLS, AcquireX Deep Scan, ddMS2	99.1, 95.1, 97.8, 98.9	2.782
Citraconic acid	P. lanceolata leaf	ddMS3, RTLS, AcquireX Deep Scan, ddMS2	95.3, 94.2, 93.6, 86.9	0.585
Corchoionoside C	Both	ddMS3, RTLS, AcquireX Deep Scan, ddMS2	99.5, 99.6, 99.1, 99.5	3.405

Corchorifatty acid F	Both	ddMS3, RTLS, AcquireX Deep Scan, ddMS2	99.1, 99.0, 99.1, 98.7	6.767
Coumarin	P. lanceolata leaf	ddMS3, RTLS, AcquireX Deep Scan, ddMS2	90.9, 91.2, 76.3, 84.1	4.961
D-(-)-Quinic acid	Both	ddMS3, RTLS, AcquireX Deep Scan, ddMS2	91.6, 95.2, 78.0, 87.7	2.781
D-Mannitol	Both	ddMS3, RTLS, AcquireX Deep Scan, ddMS2	99.0, 75.4, 86.3, 94.0	0.447
DL-PHENYLALANINE	Both	ddMS3, RTLS, AcquireX Deep Scan, ddMS2	98.3, 98.2, 97.8, 98.3	0.922
DL-Valine	Both	ddMS3, RTLS, AcquireX Deep Scan, ddMS2	98.0, 98.3, 98.8, 95.4	12.425
Dihydroalbocycline	P. lanceolata leaf	ddMS3, RTLS, AcquireX Deep Scan, ddMS2	99.7, 99.5, 99.1, 99.6	9.075
Diosmetin	P. lanceolata leaf	ddMS3, RTLS, AcquireX Deep Scan, ddMS2	99.4, 98.9, 99.8, 99.4	6.743
Esculetin	P. lanceolata leaf	ddMS3, RTLS, AcquireX Deep Scan, ddMS2	96.9, 97.1, 97.7, 97.6	3.002
Estriol	P. lanceolata leaf	ddMS3, RTLS, AcquireX Deep Scan, ddMS2	82.4, 83.7, 84.1, 82.2	8.609
Eurostoside	P. lanceolata leaf	ddMS3, RTLS, AcquireX Deep Scan, ddMS2	94.2, 96.5, 94.8, 94.9	4.672
Everlastoside D	P. lanceolata leaf	ddMS3, RTLS, AcquireX Deep Scan, ddMS2	89.7, 91.2, 86.1, 93.4	3.925
FEGXTMHRTZJSIG-RDRUSSIHSA-N	P. lanceolata leaf	ddMS3, RTLS, AcquireX Deep Scan, ddMS2	90.7, 85.1, 81.1, 85.1	3.441
FYLMQUDVZFGACW-AGBRFASWSA-N	P. lanceolata leaf	ddMS3, RTLS, AcquireX Deep Scan, ddMS2	91.3, 90.1, 91.4, 91.7	10.135
Ferulic Acid Acyl-b-D-glucoside	P. lanceolata leaf	ddMS3, RTLS, AcquireX Deep Scan	91.9, 98.3, 85.3	5.666
Ferulic acid, Z-	Both	ddMS3, RTLS, AcquireX Deep Scan, ddMS2	95.9, 97.2, 97.5, 97.4	5.667
Forsythoside B	P. lanceolata leaf	ddMS3, RTLS, AcquireX Deep Scan	97.2, 97.5, 99.2	4.288
Geniposidic Acid	Both	ddMS3, RTLS, AcquireX Deep Scan, ddMS2	96.9, 96.6, 95.8, 96.9	1.329
Genistein	Both	ddMS3, RTLS, AcquireX Deep Scan, ddMS2	85.9, 86.4, 84.4, 85.0	6.626
Gentisic acid	P. lanceolata leaf	ddMS3, RTLS, AcquireX Deep Scan, ddMS2	87.2, 87.5, 88.9, 87.9	2.484
Glu-Ser-Arg	Both	ddMS3, RTLS, AcquireX Deep Scan, ddMS2	98.1, 97.4, 98.7, 97.2	3.139

Glu-Trp-Pro	P. lanceolata leaf	ddMS3, RTLS, AcquireX Deep Scan, ddMS2	80.1, 75.2, 68.8, 79.3	5.178
Glucosilsteviol	P. lanceolata leaf	ddMS3, RTLS, AcquireX Deep Scan	89.2, 89.1, 89.2	8.366
Grevillol	P. lanceolata leaf	ddMS3, RTLS, AcquireX Deep Scan, ddMS2	83.9, 92.8, 91.0, 89.4	10.375
Guvacoline	P. lanceolata leaf	ddMS3, RTLS, AcquireX Deep Scan, ddMS2	88.8, 89.2, 91.8, 88.7	1.203
HRTKMOMTMZCGLF-QHHAFSJGSA-N	Both	ddMS3, RTLS, AcquireX Deep Scan, ddMS2	76.2, 85.2, 78.0, 77.0	4.532
HZUURLOSYMEMET-QPJJXVBHSA-N	Both	ddMS3, RTLS, AcquireX Deep Scan, ddMS2	99.3, 99.4, 84.0, 99.1	4.297
Hydroxytyrosol	P. lanceolata leaf	ddMS3, RTLS, AcquireX Deep Scan, ddMS2	66.9, 86.6, 63.5, 89.2	1.516
IKRMNTDCFLRTRS-QIDSOVCESA-N	P. lanceolata leaf	ddMS3, RTLS, AcquireX Deep Scan, ddMS2	99.8, 99.6, 99.5, 99.9	6.369
Icariside F2	P. lanceolata leaf	ddMS3, RTLS, AcquireX Deep Scan	94.0, 94.8, 96.1	3.282
Indole-3-acetic acid	P. lanceolata leaf	ddMS3, RTLS, AcquireX Deep Scan, ddMS2	88.7, 93.0, 79.8, 87.5	4.086
Irinotecan	P. lanceolata leaf	ddMS3, RTLS, AcquireX Deep Scan, ddMS2	83.7, 83.4, 82.0, 81.6	12.558
JMVJEEIMQLTXCI-HZJYTTRNSA-N	P. lanceolata leaf	ddMS3, RTLS, AcquireX Deep Scan, ddMS2	96.0, 97.8, 95.1, 75.9	10.142
JRGYPGLKSRGIHK-NQTNIMIGSA-N	P. lanceolata leaf	ddMS3, RTLS, AcquireX Deep Scan, ddMS2	95.6, 95.1, 92.8, 94.7	12.513
Ketologanic acid	Both	ddMS3, RTLS, AcquireX Deep Scan, ddMS2	99.8, 99.8, 99.6, 99.8	1.322
L-isoleucine	P. lanceolata leaf	ddMS3, RTLS, AcquireX Deep Scan, ddMS2	90.0, 93.0, 99.3, 89.1	0.582
LFKQVVDFNHDYNK-XGAYTTIXSA-N	P. lanceolata leaf	ddMS3, RTLS, AcquireX Deep Scan, ddMS2	93.6, 94.8, 90.5, 89.7	4.281
LPLWWIHUJXWQSS-IMNRLACTSA-N	P. lanceolata leaf	ddMS3, RTLS, AcquireX Deep Scan	99.9, 99.9, 99.5	4.946
Lamiuamplexoside C	Both	ddMS3, RTLS, AcquireX Deep Scan, ddMS2	96.6, 96.8, 95.1, 90.1	4.694
Lariciresinol 4-O-glucoside	P. lanceolata leaf	ddMS3, RTLS, AcquireX Deep Scan, ddMS2	93.9, 95.2, 75.8, 69.9	4.157
Licocoumarone	P. lanceolata leaf	ddMS3, RTLS, AcquireX Deep Scan, ddMS2	88.5, 82.7, 62.3, 86.9	4.954
Linoleoyl ethanolamide	Both	ddMS3, RTLS, AcquireX Deep Scan, ddMS2	96.8, 95.7, 92.6, 97.2	11.373

Linolic acid	P. lanceolata leaf	ddMS3, RTLS, AcquireX Deep Scan, ddMS2	98.4, 95.7, 94.5, 98.9	11.017
Loganic acid	Both	ddMS3, RTLS, AcquireX Deep Scan, ddMS2	97.8, 94.5, 98.2, 97.8	2.093
Lonfuranacid A	P. lanceolata leaf	ddMS3, RTLS, AcquireX Deep Scan, ddMS2	93.4, 77.2, 91.6, 76.9	5.244
Lugrandoside	Both	ddMS3, RTLS, AcquireX Deep Scan, ddMS2	96.9, 96.5, 98.0, 97.3	4.279
Luteolin	P. lanceolata leaf	ddMS3, RTLS, AcquireX Deep Scan, ddMS2	94.5, 86.5, 96.1, 95.7	5.943
Luteolin 4'-O-glucoside	Both	ddMS3, RTLS, AcquireX Deep Scan, ddMS2	97.4, 99.3, 84.6, 88.7	4.462
MECYDNMVWUSMSU-YTLDOUCOSA-N	P. lanceolata leaf	ddMS3, RTLS, AcquireX Deep Scan, ddMS2	70.7, 83.4, 82.7, 83.2	8.421
Maleic acid	P. lanceolata leaf	ddMS3, RTLS, AcquireX Deep Scan, ddMS2	98.3, 88.7, 99.6, 81.5	0.513
Malonic acid	Both	ddMS3, RTLS, AcquireX Deep Scan, ddMS2	98.2, 98.1, 96.1, 97.8	0.541
Manidipine	P. lanceolata leaf	ddMS3, RTLS, AcquireX Deep Scan, ddMS2	61.8, 60.3, 63.3, 80.2	12.79
Matairesinoside	P. lanceolata leaf	ddMS3, RTLS, AcquireX Deep Scan, ddMS2	80.4, 80.2, 78.4, 79.7	4.391
Methyl 2-{[2-O-(6-deoxy-α-L-mannopyranosyl)-β-D-glucopyranosyl]oxy}benzoate	P. lanceolata leaf	ddMS3, RTLS, AcquireX Deep Scan, ddMS2	99.9, 99.9, 99.7, 99.9	3.454
Mussaenosidic acid	P. lanceolata leaf	ddMS3, RTLS, AcquireX Deep Scan, ddMS2	94.0, 97.6, 92.8, 68.5	1.148
Myrciaphenone A	Both	ddMS3, RTLS, AcquireX Deep Scan, ddMS2	70.0, 85.8, 69.5, 92.2	1.258
N,N-Dimethyl-4-{5-[(3S)-1-(phenylsulfonyl)-3-pyrrolidinyl]-1,3,4-oxadiazol-2-yl}aniline	P. lanceolata leaf	ddMS3, RTLS, AcquireX Deep Scan, ddMS2	97.5, 96.9, 98.0, 97.7	3.151
N-({(1S,4S,6S)-6-Isopropyl-3-methyl-4-[2-oxo-2-(1-pyrrolidinyl)ethyl]-2-cyclohexen-1-yl}methyl)-2-pyrazinecarboxamide	P. lanceolata leaf	ddMS3, RTLS, AcquireX Deep Scan, ddMS2	99.5, 99.3, 99.9, 99.1	8.388
N-Ethyl-2-[(14E,16S,17S)-8-(4-morpholinyl)-2-oxo-12-oxa-1,4-diazatricyclo[14.3.1.06,11]icosa-6,8,10,14-tetraen-17-yl]acetamide	P. lanceolata leaf	ddMS3, RTLS, AcquireX Deep Scan, ddMS2	99.8, 99.7, 99.7, 99.6	8.506
N-Isovalerylglycine	P. lanceolata leaf	ddMS3, RTLS, AcquireX Deep Scan, ddMS2	90.8, 98.8, 93.9, 96.9	3.178
N-{(1S,2S,4aS,7S,8S,8aS)-8-Hydroxy-1,4a-dimethyl-7-[(2S)-1-oxo-1-(1-piperidinyl)-2-propanyl]decahydro-2-	P. lanceolata leaf	ddMS3, RTLS, AcquireX Deep Scan, ddMS2	99.4, 99.4, 99.2, 99.2	10.248

naphthalenyl}-5-pyrimidinecarboxamide				
N1-(4-{3-[5-(trifluoromethyl)-2-pyridyl]-4,5-dihydro-1H-1,2,4-triazol-5-yl}phenyl)acetamide	P. lanceolata leaf	ddMS3, RTLS, AcquireX Deep Scan, ddMS2	95.6, 94.1, 96.5, 91.0	0.914
N1-cyclohexyl-2-[{2-[(4-chlorophenyl)thio]acetyl}(methyl)amino]benzamide	P. lanceolata leaf	ddMS3, RTLS, AcquireX Deep Scan, ddMS2	99.4, 99.4, 98.6, 98.5	4.099
Neochlorogenic acid	P. lanceolata leaf	ddMS3, RTLS, AcquireX Deep Scan, ddMS2	92.9, 93.1, 92.0, 93.0	1.787
Nicotinamide	P. lanceolata leaf	ddMS3, RTLS, AcquireX Deep Scan, ddMS2	96.0, 84.9, 90.9, 94.9	0.528
Norharman	P. lanceolata leaf	ddMS3, RTLS, AcquireX Deep Scan, ddMS2	96.1, 85.7, 97.6, 96.7	3.111
OGAIGVUECHDBJB-SKPUKWKESA-N	P. lanceolata leaf	ddMS3, RTLS, AcquireX Deep Scan, ddMS2	96.7, 97.4, 97.5, 97.2	4.234
OQWOKDQAPBSVGH-UHFFFAOYSA-N	P. lanceolata leaf	ddMS3, RTLS, AcquireX Deep Scan, ddMS2	94.1, 62.6, 91.4, 93.2	9.818
OZHUIFOZCHBIOL-KUDUMFAVSA-N	P. lanceolata leaf	ddMS3, RTLS, AcquireX Deep Scan, ddMS2	90.0, 91.7, 95.3, 89.9	5.545
Octadecenedioic acid	P. lanceolata leaf	ddMS3, RTLS, AcquireX Deep Scan	97.5, 89.4, 95.6	8.921
Oleamide	Both	ddMS3, RTLS, AcquireX Deep Scan, ddMS2	92.4, 93.8, 97.0, 93.9	12.558
Oleanolic acid	P. lanceolata leaf	ddMS3, RTLS, AcquireX Deep Scan, ddMS2	98.8, 95.3, 76.9, 97.4	12.417
Oleic acid alkyne	P. lanceolata leaf	ddMS3, RTLS, AcquireX Deep Scan, ddMS2	99.9, 100.0, 100.0, 100.0	10.25
Oleoyl ethanolamide	P. lanceolata leaf	ddMS3, RTLS, AcquireX Deep Scan, ddMS2	95.4, 97.8, 95.4, 92.5	11.967
PEG Monolaurate n5	P. lanceolata leaf	ddMS3, RTLS, AcquireX Deep Scan, ddMS2	100.0, 99.7, 99.9, 99.9	10.869
Palmitoyl ethanolamide	P. lanceolata leaf	ddMS3, RTLS, AcquireX Deep Scan	97.6, 99.4, 97.1	11.522
Pentoxifylline	P. lanceolata leaf	ddMS3, RTLS, AcquireX Deep Scan, ddMS2	98.4, 99.1, 98.9, 97.9	1.188
Phe-Ile-Gln	P. lanceolata leaf	ddMS3, RTLS, AcquireX Deep Scan, ddMS2	99.7, 99.4, 99.7, 99.7	2.795
Phenethyl sophoroside	Both	ddMS3, RTLS, AcquireX Deep Scan, ddMS2	85.8, 92.0, 69.0, 84.0	3.426
Phenylethyl primeveroside	P. lanceolata leaf	ddMS3, RTLS, AcquireX Deep Scan, ddMS2	97.3, 98.9, 98.9, 94.3	3.019
Phlinoside A	Both	ddMS3, RTLS, AcquireX Deep Scan	81.2, 81.4, 81.4	4.389

Phlomisionoside	Both	ddMS3, RTLS, AcquireX Deep Scan, ddMS2	99.7, 89.6, 97.7, 99.7	3.392
Piliformic acid	P. lanceolata leaf	ddMS3, RTLS, AcquireX Deep Scan, ddMS2	96.8, 91.1, 96.1, 87.9	6.436
Plantainoside C	P. lanceolata leaf	ddMS3, RTLS, AcquireX Deep Scan, ddMS2	99.9, 86.8, 90.6, 99.0	5.046
Protocatehuic acid	P. lanceolata leaf	ddMS3, RTLS, AcquireX Deep Scan, ddMS2	91.4, 83.0, 89.7, 76.3	1.48
QRUIPHBZVJDDTR-KTQHHXNFSA-N	P. lanceolata leaf	ddMS3, RTLS, AcquireX Deep Scan, ddMS2	99.1, 97.2, 87.2, 97.5	11.33
RBTWPFBRVJRSRZ-VLBJUCMXSA-N	P. lanceolata leaf	ddMS3, RTLS, AcquireX Deep Scan	98.0, 96.6, 99.8	4.483
RLGRBYHBNWLGER-RMKNXTFCSA-N	P. lanceolata leaf	ddMS3, RTLS, AcquireX Deep Scan, ddMS2	99.9, 99.8, 99.9, 99.6	5.667
Rhamnetin 3-galactoside	Both	ddMS3, RTLS, AcquireX Deep Scan	78.4, 77.3, 95.0	4.815
SAHCQBPGXQFTRA-MTWZWZNHSA-N	Both	ddMS3, RTLS, AcquireX Deep Scan, ddMS2	96.6, 60.6, 87.5, 88.3	10.234
SUFSOKMJLLAQQX-JYBASQMISA-N	P. lanceolata leaf	ddMS3, RTLS, AcquireX Deep Scan, ddMS2	79.0, 77.6, 93.4, 90.6	6.92
SXLKGCCRNBGMMM-VDFSFMFYSA-N	P. lanceolata leaf	ddMS3, RTLS, AcquireX Deep Scan, ddMS2	99.0, 99.3, 98.1, 99.0	2.921
Salicylic acid	P. lanceolata leaf	ddMS3, RTLS, AcquireX Deep Scan, ddMS2	97.8, 97.9, 83.7, 93.2	4.792
Scutellarin	P. lanceolata leaf	ddMS3, RTLS, AcquireX Deep Scan, ddMS2	91.2, 90.5, 91.5, 91.3	4.518
Sebacic acid	P. lanceolata leaf	ddMS3, RTLS, AcquireX Deep Scan, ddMS2	99.4, 90.5, 98.5, 99.3	5.875
Secologanin	P. lanceolata leaf	ddMS3, RTLS, AcquireX Deep Scan, ddMS2	98.9, 99.4, 91.0, 98.2	2.502
Secosterigmatocystin	Both	ddMS3, RTLS, AcquireX Deep Scan, ddMS2	98.7, 99.0, 74.1, 97.3	0.767
Sparfloxacin	P. lanceolata leaf	ddMS3, RTLS, AcquireX Deep Scan	94.5, 99.5, 85.5	4.029
Spiroxamine	Both	ddMS3, RTLS, AcquireX Deep Scan	97.3, 97.4, 96.8	9.923
Squamocin L	P. lanceolata leaf	ddMS3, RTLS, AcquireX Deep Scan, ddMS2	72.7, 97.6, 72.2, 72.7	13.164
Succinic acid	Both	ddMS3, RTLS, AcquireX Deep Scan, ddMS2	99.7, 99.7, 99.6, 99.7	0.685
Sucrose	P. lanceolata leaf	ddMS3, RTLS, AcquireX Deep Scan, ddMS2	99.6, 99.7, 99.3, 99.5	0.46

Teucardoside	P. lanceolata leaf	ddMS3, RTLS, AcquireX Deep Scan, ddMS2	99.8, 99.9, 99.7, 99.8	3.332
Trans-aconitic acid	Both	ddMS3, RTLS, AcquireX Deep Scan, ddMS2	99.5, 99.0, 99.4, 99.4	0.588
Traumatic acid	P. lanceolata leaf	ddMS3, RTLS, AcquireX Deep Scan, ddMS2	98.3, 96.2, 91.9, 93.5	7.199
